# Next-Generation Sequencing Data-Based Association Testing of a Group of Genetic Markers for Complex Responses Using a Generalized Linear Model Framework

**DOI:** 10.3390/math11112560

**Published:** 2023-06-02

**Authors:** Zheng Xu, Song Yan, Cong Wu, Qing Duan, Sixia Chen, Yun Li

**Affiliations:** 1 Department of Mathematics and Statistics, Wright State University, Dayton, Ohio, 45324, USA;; 2 Department of Biostatistics, University of North Carolina at Chapel Hill, Chapel Hill, NC 27599, USA;; 3 Department of Genetics, University of North Carolina at Chapel Hill, Chapel Hill, NC 27599, USA;; 4 Department of Computer Science, University of North Carolina at Chapel Hill, Chapel Hill, NC 27599, USA;; 5 Department of Computer Science and Engineering, University of Nebraska-Lincoln, Lincoln, NE 68508, USA;; 6 Cincinnati Children’s Hospital Medical Center, Cincinnati, OH 45229, USA;; 7 Department of Biostatistics and Epidemiology, University of Oklahoma Health Sciences Center, Oklahoma City, OK 73104, USA

**Keywords:** Association study, Genotype calling, Next-generation sequencing, Group testing, Rare variant, Score test, Joint significance test, Variable collapse test, Generalized linear model, 62P10, 92B15, 62J05

## Abstract

Association testing has been widely used to study the relationship between genetic variants and phenotypes. Most association testing methods are genotype-based, i.e. first estimate genotype and then regress phenotype on estimated genotype and other variables. Directly testing methods based on next generation sequencing (NGS) data without genotype calling have been proposed and shown advantage over genotype-based methods in the scenarios when genotype calling is not accurate. NGS data-based single-variant testing have been proposed including our previously proposed single-variant testing method, i.e. UNC combo method [1]. NGS data-based group testing methods for continuous phenotype have also been proposed by us using a linear model framework which can handle continuous responses [2]. In this paper, we extend our linear model-based framework to a generalized linear model-based framework so that the methods can handle other types of responses especially binary responses which is commonly-faced in association studies. We have conducted extensive simulation studies to evaluate the performance of different estimators and compare our estimators with their corresponding genotype-based methods. We found that all methods have Type I errors controlled, and our NGS data-based testing methods have better performance than their corresponding genotype-based methods in the literature for other types of responses including binary responses (logistic regression) and count responses (Poisson regression especially when sequencing depth is low. In conclusion, we have extended our previous linear model (LM) framework to a generalized linear model (GLM) framework and derived NGS data-based testing methods for a group of genetic variants. Compared with our previously proposed LM-based methods [2], the new GLM-based methods can handle more complex responses (for example, binary responses and count responses) in addition to continuous responses. Our methods have filled the literature gap and shown advantage over their corresponding genotype-based methods in the literature.

## 1. Introduction

With recent advances in sequencing technology, next generation sequencing (NGS) has become more popular in genetic association studies. compared with traditional technologies such as Sanger sequencing [3], NGS technologies have higher sequencing throughput and lower costs. enormous sequencing data have been generated for genetic studies from NGS platforms (for example: Solid and Solexa) [4,5].

Next generation sequencing (NGS) data in the format of raw sequencing reads are generated and collected using NGS platforms [6–8]. There are no genotypes provided by these platforms. To obtain genotype data, multi-step bioinformatic data-processing pipelines have to be conducted including quality control (QC), sequence alignment/mapping, genetic variant calling and genotype calling (GC) [7,9,10]. Pipelines to obtain genotypes based on NGS data have been proposed [11–13]. After obtaining estimated genotypes, researchers conduct regression analysis to study how genotypes and other variables (environmental variables, clinical variables, etc) are associated with phenotypes [14–16].

Regression models have been used to develop association testing approaches. Phenotypes are responses. Genotypes and other variables including environmental variables and behavior variables are explanatory variables/predictors in regression analysis. Depending on different types of responses, different regression models can be adopted. For example, bio-statisticians typically conduct linear regression for continuous responses, and logistics regression for binary responses. If the response is an integer/count type, a Poisson regression can be adopted. To handle complex responses (various types), the framework of a generalized linear model (GLM) can be adopted, which is better than a linear model (LM) framework dealing with only continuous responses as we proposed before. This motivates us to extend our previous linear model framework [2] to a general linear model framework in this article.

To test for association between genetic variables and the phenotype, different strategies are recommended depending on whether genetic variables are *common variants* or *rare variants*. A genetic marker can be classified as common variant or rare variant depending on whether its minor allele frequency (MAF) is higher or lower than a threshold value c, which is between 0.01 and 0.05 [17,18]. For common markers, association studies (AS) are typically single-variant testing assuming a genetic dominant model or recessive model or additive model, and genome-wide association studies (GWAS) refer to repeating the single-variant testing to all common genetic variants genomewide [14,19]. For common variants, joint significance of a group of markers can also be tested using Chi-square test or F tests. Group testing can be gene-based, pathway-based or range-based. For example, gene-based group testing treat all variants within a gene as a group and conduct group testing. All genetic markers in the same group are tested simultaneously using a group test. Genome-wide group testing refers to repeating the individual group testing for all groups genome-wide.

Rare variants are characteristics of low variations in genotypes since their minor allele frequencies (MAF) are less than a pre-specified threshold value, typically 0.05. Thus, there may not be enough power for association testing of a single rare variant [17,20]. Therefore, testing of rare variants is usually group-based instead of individual-variant based. A range of rare-variant testing methods have been proposed. Among these tests, two big categories of rare-variant testing methods are widely used. The first category refers to variable collapsing (VC) testing methods, which first combine multiple genetic variables into one variable, or calculate a value based on multiple genetic variables, and then conduct association studies between the phenotype and the merged/collapsed/calculated variable [21,22]. (2) The second category refers to variants of Sequence Kernel Association testing (SKAT) methods including SKAT, MK-SKAT, BESKAT and SKAT-O [17,21,23,24].

The burden test and the SKAT test are *respectively* the representative methods of the two categories of rare-variant testing methods [22,24]. The two tests have been widely used in association studies of a group of rare variants with phenotypes. Both tests are genotype-based tests in that they *first conduct genotype calling* to obtain or estimate genotypes, and then conduct association testing based on phenotypes and the *estimated genotypes*. Burden test first calculates the sum of rare alleles and then conducts regression of the phenotype on the number of rare alleles and other covariates [22,25]. SKAT adopts a linear-model framework (linear regression) to deal with continuous phenotypes, and a logistics-model framework (logistics regression to deal with binary phenotypes [24]. Continuous type and binary type are two mostly-used types of phenotypes in association studies, thus our focus in this article are the two types.

However, there are estimation errors in obtaining genotypes by genotype calling. There are multiple factors influencing genotyping accuracy such as mapping accuracy, sequencing errors and sequencing depth. In the scenario of low sequencing depth, genotype calling can be very imprecise, which can influence downstream genotype-based association studies [26,27]. To improve the performance of association testing, NGS-based methods without genotype calling have been proposed. Directly modeling next generation sequencing (NGS) data instead of genotypes considers uncertainty and estimation errors in genotype calling and improves statistical performances in association testing [1,28].

Researchers have proposed a range of NGS data-based single-variant association testing methods without genotype calling [1,28,29] including our previously proposed UNC combo method [1]. These NGS data-based single-variant testing methods can achieve better performance under the scenario of low sequencing depth, heterogeneous sequencing depths, and imprecise genotype calls [1,28,29]. However, there are no NGS data-based group testing methods in the literature except our previously proposed linear model (LM)-based group testing methods [2]. Being linear model-based, our previously proposed method can only handle continuous phenotypes. However, in the fields of bio-statistics and bio-informatics especially association studies, other types of phenotypes especially binary phenotypes (such as disease status and case/control association studies) are widely encountered. It is greatly desired and necessary to extend our methods to enable handling of other types of phenotypes especially binary phenotypes. Thus, we extend our method from a linear model (LM)-based framework to a generalized linear model (GLM)-based NGS data-based group testing method so that our proposed methods can handle complex responses including continuous responses (linear regression), binary responses (logistics regression) and count/integer responses (Poisson regression). The proposed NGS-based group testing methods are expected to have better performance corresponding to their corresponding genotype-based methods especially under the scenario of low sequencing depth where genotype calling/estimation is not accurate.

Corresponding to genotype-based group-testing methods for common variants (joint significance test) and rare variants (variable collapse test) [21,24,30], we fill the literature gap by proposing their corresponding NGS data-based methods without genotype calling, i.e. the *joint significance test (JS)* for a group of *common variants* and the *variable collapse test (VC)* for a group of *rare variants*. Compared with our previous work (Xu, 2023), the major contribution of this work is that it can handle a range of phenotypes including continuous, binary and count/integer phenotypes using the generalized linear model framework whereas our previous work [2] can only handle continuous phenotypes using the linear model framework.

The rest of the article is organized as follows. Section 2 states the methodology including sequence data-based Joint Significance Test and variance collapse test. Section 3 shows the results of our simulation studies. Section 4 makes discussion and Section 5 draws conclusions.

## 2. Methodology

Suppose there are N individuals. For individual i, we have $\left(y_{i},g_{i},x_{i}\right)$, where $y_{i}$ is the phenotype, $g_{i}$ represents the genotypes, and $x_{i}$ represents additional covariats such as age, gender, and environmental variables. Assume the genetic variants under consideration are bi-allelic so that the genotypes can only take values of 0, 1, and 2.

Suppose we consider a group of $d_{g}$ genetic variables in the test, so that the genotypes of individual i are represented as a row vector $g_{i}=\left(g_{i1},g_{i2},\ldots ,g_{i_{d_{g}}}\right)$, where $g_{ij}$ is the genotype value at the j-th genetic variant for individual i. We allow our test to include $d_{x}$ additional covarites, and let the row vector $x_{i}=\left(1,x_{i1},x_{i2},\ldots ,x_{id_{x}}\right)$ represent the intercept and $d_{x}$ additional covariates, where $x_{ij}$ is the value of the j-th additional covariate for individual i.

For N individuals under consideration, we have the genotype matrix of size $N\times d_{g}$ as $g=\left(g_{1},g_{2},\ldots ,g_{N}\right)$, response vector of length n as $y=\left(y_{1},y_{2},\ldots ,y_{N}\right)$, and additional covariates matrix of size $N\times \left(d_{x}+1\right)$ as $x=\left(x_{1},x_{2},\ldots ,x_{N}\right)$.

### 2.1. Model Complex Phenotypes Using a Generalized Linear Model Framework

Both the linear model for a quantitative phenotype and the logistic regression model for a binary phenotype conform the framework of general linear model [31]. This motivates us to model complex phenotypes by a generalized linear model (GLM) framework. The GLM model-based derivation is a direct extension of our previous linear model (LM)-based framework [2], which is a laborious extension of Skotte *et al*. (2012)’s work on NGS-based single-variant testing [28].

We model a complex phenotype using a generalized linear model [31,32]. For individual i, the probability of observing phenotype $y_{i}$ is modelled to be

(1) $$p\left(y_{i}|x_{i},g_{i}\right)=p_{\alpha ,\beta ,\phi }\left(y_{i}|x_{i},g_{i}\right)=exp\left(\frac{y_{i}\eta_{i}-b\left(\eta_{i}\right)}{a(\phi )}+c\left(y_{i},\phi \right)\right),$$

where the row vector $\alpha \in {ℛ}^{d_{x}+1}$, the row vector $\beta \in {ℛ}^{d_{g}}$, and the linear predictor $\eta_{i}=\eta_{\alpha ,\beta }\left(x_{i},g_{i}\right)=\alpha x_{i}^{T}+\beta g_{i}^{T}$, with appropriate choice of the function a(.),b(.) and c(.,.), depending on the type of the phenotype under consideration, including

- the continuous phenotype, corresponding to a linear regression;
- the binary phenotype, corresponding to a logistics regression;
- the count (integer) phenotype, corresponding to a Poisson regression.

To be more specific, we show how the modeling of the three types of phenotype belongs to our generalised linear model (GLM) framework. First, consider a continuous phenotype as specified in our previous linear model (LM) framework [2], i.e. $y_{i}\sim N\left(\eta_{i},\sigma^{2}\right)$, where $\eta_{i}=\alpha x_{i}^{T}+\beta g_{i}^{T}$. Our previously proposed LM framework is a special case of our GLM framework because

$$\begin{array}{ll} f\left(y_{i}|x_{i},g_{i}\right) & \;=\frac{1}{\sqrt{2\pi \sigma^{2}}}exp\left(-\frac{{\left(y_{i}-\eta_{i}\right)}^{2}}{2\sigma^{2}}\right)=exp\left(\frac{y_{i}\eta_{i}-\eta_{i}^{2}/2}{\sigma^{2}}-\frac{y_{i}^{2}}{2}-\frac{ln\left(2\pi \sigma^{2}\right)}{2}\right)\\ & \;=exp\left(\frac{y_{i}\eta_{i}-b\left(\eta_{i}\right)}{a(\phi )}+c\left(y_{i},\phi \right)\right). \end{array}$$

Thus, $\phi =\sigma^{2},a(\phi )=\phi ,b\left(\eta_{i}\right)=\eta_{i}^{2}/2$ and $c\left(y_{i},\phi \right)=-y_{i}^{2}/(2\phi )-ln(2\pi \phi )/2$ for a continuous phenotype.

Next, consider a binary phenotype modelled using a logistics regression model, i.e., $\pi_{i}=P\left(y_{i}=1|x_{i},g_{i}\right)$, and $ln\left(\pi_{i}/\left(1-\pi_{i}\right)\right)=\eta_{i}=\alpha x_{i}^{T}+\beta g_{i}^{T}$. This logistics model is a special case of our GLM framework because

$$\begin{array}{ll} f\left(y_{i}|x_{i},g_{i}\right) & \;=\pi_{i}{\left(1-\pi_{i}\right)}^{1-y_{i}}={\left(\frac{\pi_{i}}{1-\pi_{i}}\right)}^{y_{i}}\left(1-\pi_{i}\right)=exp\left(\eta_{i}y_{i}+ln\left(1-\pi_{i}\right)\right)\\ & \;=\exp\left(y_{i}\eta_{i}-\ln\left(1+e^{\eta_{i}}\right)\right)=\exp\left(\frac{y_{i}\eta_{i}-b\left(\eta_{i}\right)}{a\left(\phi \right)}+c\left(y_{i},\phi \right)\right). \end{array}$$

Thus, $a(\phi )=1,b\left(\eta_{i}\right)=ln\left(1+e^{\eta_{i}}\right)$ and $c\left(y_{i},\phi \right)=0$ for a binary response.

Thirdly, consider a count (integer) phenotype modelled using a Poisson regression model, i.e. $Y_{i}\sim Poisson\left(\lambda_{i}=e^{\eta_{i}}\right)$, where $\eta_{i}=\alpha x_{i}^{T}+\beta g_{i}^{T}$. This Poisson regression is also a special case of our GLM framework because

$$\begin{array}{ll} f\left(y_{i}|x_{i},g_{i}\right) & \;=\frac{\lambda_{i}^{y_{i}}}{y_{i}!}e^{-\lambda_{i}}=\frac{e^{\eta_{i}y_{i}}}{y_{i}!}e^{-exp\left(\eta_{i}\right)}\\ & \;=\exp\left(y_{i}\eta_{i}-e^{\eta_{i}}-\ln\left(y_{i}!\right)\right)=\exp\left(\frac{y_{i}\eta_{i}-b\left(\eta_{i}\right)}{a\left(\phi \right)}+c\left(y_{i},\phi \right)\right). \end{array}$$

Thus, $a(\phi )=1,b\left(\eta_{i}\right)=e^{\eta_{i}}$ and $c\left(y_{i},\phi \right)=-ln\left(y_{i}!\right)$ for a count (integer) response.

Our proposed GLM framework is a general framework which can handle different types of responses. The distribution of the phenotype $y_{i}$ depends on the individual predictors $\left(x_{i}\right.$ and $\left.g_{i}\right)$ as well as the parameters (α,β,ϕ). When a(ϕ) and $c\left(y_{i},\phi \right)$ are constant functions with respect to ϕ, we can drop ϕ out and denote the functions as a and $c\left(y_{i}\right)$. For example, in logistics regression, a(ϕ)=1 and $c\left(y_{i},\phi \right)=0$, and in Poisson regression, a(ϕ)=1 and $c\left(y_{i},\phi \right)=-ln\left(y_{i}!\right)$. The parameters are (α,β) not involving ϕ. Then the distribution of $y_{i}$ depends on $\left(x_{i}\right.$ and $\left.g_{i}\right)$ as well as the parameters (α,β). In the following, we will discuss two situations: (1) the situation when parameters are (α,β,ϕ); (2) the situation when parameters are (α,β), i.e. ϕ is dropped out.

### 2.2. Uncertain Genotypes

Since the actual genotypes remain unobserved in NGS studies, we directly model the joint distribution of the phenotypes and the observed sequencing data. Because phenotypes are conditionally independent of the sequencing data given true genotypes, the density of the joint distribution can be factorized as

(2) $$p_{\theta }\left(y_{i},D_{i}|x_{i}\right)=\sum_{g\in 𝒢}\;f_{\theta }\left(y_{i}|x_{i},g\right)h\left(g,D_{i}\right),$$

where θ=(α,β,ϕ) or θ=(α,β) depending on whether ϕ can be dropped out, i.e. whether a(ϕ) and $c\left(y_{i},\phi \right)$ are constant functions with respect to ϕ. The terms $y_{i},D_{i},x_{i}$ are *respectively* the phenotype, sequencing reads and additional covariates for individual i. The term 𝒢 is the genotype state space including all possible genotype values g, which means that in Equation (2), the summation is over all possible values of g. Since there are $d_{g}$ genetic markers in testing, and each genetic marker takes possible values of 0, 1, 2, then $𝒢=\{0,\;1,\;2{\}}^{d_{g}}$. The term h in Equation (2) is the shorthand notation for the joint distribution of the genotype and the observed sequencing data, i.e., $h\left(g,D_{i}\right)=p\left(D_{i}|g\right)p(g|\overset{ˆ}{f})$, where $\overset{ˆ}{f}$ is the estimated allele frequency, as modeled in Skotte *et al*. (2012) [28]. The log-likelihood function for the model with genotype uncertainties (i.e. latent genotypes) thus becomes

(3) $$l_{y,D}\left(\theta \right)=\sum_{i=1}^{N}\;\log\left\{p_{\theta }\left(y_{i},D_{i}|x_{i}\right)\right\}=\sum_{i=1}^{N}\;\log\left\{\sum_{g\in 𝒢}\;\;f_{\theta }\left(y_{i}|x_{i},g\right)h\left(g,D_{i}\right)\right\},$$

where θ=(α,β,ϕ) or (α,β) depending on whether ϕ is dropped out.

In this model, testing for genetic effects means testing with the null hypothesis $H_{0}:\beta =0$. Under this null hypothesis, the density f does not depend on the genotype so that it can be extracted out of the summation.

Thus the log-likelihood function under this null hypothesis $\left(H_{0}:\beta =0\right)$ can be simplified as follows:

$$\begin{array}{ll} l_{y,D}(\alpha ,0,\phi ) & \;=\sum_{i=1}^{N}\;\;\left(ln\left(\sum_{g\in 𝒢}\;\;f_{\theta }\left(y_{i}|x_{i},g\right)h\left(g,D_{i}\right)\right)\right)\\ & \;=\sum_{i=1}^{N}\;\;\left\{\frac{y_{i}\left(\alpha x_{i}^{T}\right)-b\left(\alpha x_{i}^{T}\right)}{a(\phi )}+c\left(y_{i},\phi \right)\right\}+\text{ constant in terms of parameters, } \end{array}$$

when ϕ is not dropped out and the parameters are (α,β,ϕ). When ϕ is dropped out and the parameters are (α,β), the formula is

$$\begin{array}{ll} l_{y,D}(\alpha ,0) & \;=\sum_{i=1}^{N}\;\;\left(ln\left(\sum_{g\in 𝒢}\;\;f_{\theta }\left(y_{i}|x_{i},g\right)h\left(g,D_{i}\right)\right)\right)\\ & \;=\sum_{i=1}^{N}\;\;\left\{\frac{y_{i}\left(\alpha x_{i}^{T}\right)-b\left(\alpha x_{i}^{T}\right)}{a}+c\left(y_{i}\right)\right\}+\text{ constant in terms of parameters. } \end{array}$$

We have provided full derivation details for this simplification in Appendix A.

Therefore, the constrained MLE under the null hypothesis $\left(H_{0}:\beta =0\right)$ can be easily found from the linear regression of phenotype y on the additional covariates x only, when no genotypes g show up in the formula $\eta_{i}=\alpha x_{i}^{T}+\beta g^{T}$ under $H_{0}:\beta =0$. This motivates us to develop our method using the score test because the score test considers the constrained MLE under $H_{0}:\beta =0$.

### 2.3. Joint Significance Test for a Group of Common Genetic Variants

#### 2.3.1. The situation when parameters are (α,β,ϕ)

We use a standard score test with our null hypothesis [33,34]. When the parameters are (α,β,ϕ), the score function is

(4) $$s_{y,D}(\alpha ,\beta ,\phi )=\left[\begin{array}{l} \partial l_{y,D}(\alpha ,\beta ,\phi )/\partial \alpha^{T}\\ \partial l_{y,D}(\alpha ,\beta ,\phi )/\partial \beta^{T}\\ \partial l_{y,D}(\alpha ,\beta ,\phi )/\partial \phi \end{array}\right],$$

where α is a row vector of length $d_{x}+1,\beta$ is a row vector of length $d_{g}$, and ϕ is a scalar. The analytical formula of the score function is derived to be

$$s_{y,D}(\alpha ,\beta ,\phi )=\sum_{i=1}^{N}\;\left[{\left\{p_{\theta }\left(y_{i},D_{i}|x_{i}\right)\right\}}^{-1}\sum_{g\in 𝒢}\;\;f_{\theta }\left(y_{i}|x_{i},g\right)\left[\begin{array}{c} \frac{y_{i}-b^{'}\left(\eta_{i}\right)}{a(\phi )}x_{i}^{T}\\ \frac{y_{i}-b^{'}\left(\eta_{i}\right)}{a(\phi )}g^{T}\\ -\frac{y_{i}\eta_{i}-b\left(\eta_{i}\right)}{[a(\phi ){]}^{2}}a^{'}(\phi )+\frac{\partial c\left(y_{i},\phi \right)}{\partial \phi } \end{array}\right]h\left(g,D_{i}\right)\right],$$

where $\eta_{i}=\eta \left(x_{i},g\right)=\alpha x_{i}^{T}+\beta g^{T}$. We have provided full derivation details in Appendix B.

The observed information matrix is

$$o_{y,D}(\alpha ,\beta ,\phi )=-\left[\begin{array}{ccc} \partial^{2}l_{y,D}(\alpha ,\beta ,\phi )/\partial \alpha^{T}\partial \alpha & \partial^{2}l_{y,D}(\alpha ,\beta ,\phi )/\partial \alpha^{T}\partial \beta & \partial^{2}l_{y,D}(\alpha ,\beta ,\phi )/\partial \alpha^{T}\partial \phi \\ \partial^{2}l_{y,D}(\alpha ,\beta ,\phi )/\partial \beta^{T}\partial \alpha & \partial^{2}l_{y,D}(\alpha ,\beta ,\phi )/\partial \beta^{T}\partial \beta & \partial^{2}l_{y,D}(\alpha ,\beta ,\phi )/\partial \beta^{T}\partial \phi \\ \partial^{2}l_{y,D}(\alpha ,\beta ,\phi )/\partial \phi \partial \alpha & \partial^{2}l_{y,D}(\alpha ,\beta ,\phi )/\partial \phi \partial \beta & \partial^{2}l_{y,D}(\alpha ,\beta ,\phi )/\partial \phi^{2} \end{array}\right].$$

The analytical formula for the observed information matrix $o_{y,D}(\alpha ,\beta ,\phi )$ is derived as the following with full derivation details provided in Appendix C.

$$\begin{array}{ll} & \frac{\partial^{2}l_{y,D}(\alpha ,\beta ,\phi )}{\partial \alpha^{T}\partial \alpha }=\sum_{i=1}^{N}\;\;\left(-{\left\{p_{\theta }\left(y_{i},D_{i}|x_{i}\right)\right\}}^{-2}\right)\left[\sum_{g\in 𝒢}\;\;f_{\theta }\left(y_{i}|x_{i},g\right)\frac{y_{i}-b^{'}\left(\eta_{i}\right)}{a(\phi )}x_{i}^{T}h\left(g,D_{i}\right)\right]\left[\sum_{g\in 𝒢}\;\;f_{\theta }\left(y_{i}|x_{i},g\right)\frac{y_{i}-b^{'}\left(\eta_{i}\right)}{a(\phi )}x_{i}h\left(g,D_{i}\right)\right]\\ & \;+{\left\{p_{\theta }\left(y_{i},D_{i}|x_{i}\right)\right\}}^{-1}\sum_{g\in 𝒢}\;\;f_{\theta }\left(y_{i}|x_{i},g\right)\left[\frac{{\left(y_{i}-b^{'}\left(\eta_{i}\right)\right)}^{2}}{[a(\phi ){]}^{2}}-\frac{b^{''}\left(\eta_{i}\right)}{a(\phi )}\right]x_{i}^{T}x_{i}h\left(g,D_{i}\right)\\ & \frac{\partial^{2}l_{y,D}(\alpha ,\beta ,\phi )}{\partial \alpha^{T}\partial \beta }=\sum_{i=1}^{N}\;\;\left(-{\left\{p_{\theta }\left(y_{i},D_{i}|x_{i}\right)\right\}}^{-2}\right)\left[\sum_{g\in 𝒢}\;\;f_{\theta }\left(y_{i}|x_{i},g\right)\frac{y_{i}-b^{'}\left(\eta_{i}\right)}{a(\phi )}x_{i}^{T}h\left(g,D_{i}\right)\right]\left[\sum_{g\in 𝒢}\;\;f_{\theta }\left(y_{i}|x_{i},g\right)\frac{y_{i}-b^{'}\left(\eta_{i}\right)}{a(\phi )}gh\left(g,D_{i}\right)\right]\\ & \;+{\left\{p_{\theta }\left(y_{i},D_{i}|x_{i}\right)\right\}}^{-1}\sum_{g\in 𝒢}\;\;f_{\theta }\left(y_{i}|x_{i},g\right)\left[\frac{{\left(y_{i}-b^{'}\left(\eta_{i}\right)\right)}^{2}}{[a(\phi ){]}^{2}}-\frac{b^{''}\left(\eta_{i}\right)}{a(\phi )}\right]x_{i}^{T}gh\left(g,D_{i}\right)\\ & \frac{\partial^{2}l_{y,D}(\alpha ,\beta ,\phi )}{\partial \alpha^{T}\partial \phi }=\sum_{i=1}^{N}\;\;\left(-{\left\{p_{\theta }\left(y_{i},D_{i}|x_{i}\right)\right\}}^{-2}\right)\left[\sum_{g\in 𝒢}\;\;f_{\theta }\left(y_{i}|x_{i},g\right)\frac{y_{i}-b^{'}\left(\eta_{i}\right)}{a(\phi )}x_{i}^{T}h\left(g,D_{i}\right)\right]\left[\sum_{g\in 𝒢}\;\;f_{\theta }\left(y_{i}|x_{i},g\right)\left(-\frac{y_{i}\eta_{i}-b\left(\eta_{i}\right)}{[a(\phi ){]}^{2}}a^{'}(\phi )+\frac{\partial c\left(y_{i},\phi \right)}{\partial \phi }\right)h\left(g,D_{i}\right)\right]\\ & \;+{\left\{p_{\theta }\left(y_{i},D_{i}|x_{i}\right)\right\}}^{-1}\sum_{g\in 𝒢}\;\;f_{\theta }\left(y_{i}|x_{i},g\right)\left[\left(-\frac{y_{i}\eta_{i}-b\left(\eta_{i}\right)}{[a(\phi ){]}^{2}}a^{'}(\phi )+\frac{\partial c\left(y_{i},\phi \right)}{\partial \phi }\right)\left(\frac{y_{i}-b^{'}\left(\eta_{i}\right)}{a(\phi )}\right)-\frac{y_{i}-b^{'}\left(\eta_{i}\right)}{[a(\phi ){]}^{2}}a^{'}(\phi )\right]x_{i}^{T}h\left(g,D_{i}\right)\\ & \frac{\partial^{2}l_{y,D}(\alpha ,\beta ,\phi )}{\partial \beta^{T}\partial \beta }=\sum_{i=1}^{N}\;\;\left(-{\left\{p_{\theta }\left(y_{i},D_{i}|x_{i}\right)\right\}}^{-2}\right)\left[\sum_{g\in 𝒢}\;\;f_{\theta }\left(y_{i}|x_{i},g\right)\frac{y_{i}-b^{'}\left(\eta_{i}\right)}{a(\phi )}g^{T}h\left(g,D_{i}\right)\right]\left[\sum_{g\in 𝒢}\;\;f_{\theta }\left(y_{i}|x_{i},g\right)\frac{y_{i}-b^{'}\left(\eta_{i}\right)}{a(\phi )}gh\left(g,D_{i}\right)\right]\\ & \;+{\left\{p_{\theta }\left(y_{i},D_{i}|x_{i}\right)\right\}}^{-1}\sum_{g\in 𝒢}\;\;f_{\theta }\left(y_{i}|x_{i},g\right)\left[\frac{{\left(y_{i}-b^{'}\left(\eta_{i}\right)\right)}^{2}}{[a(\phi ){]}^{2}}-\frac{b^{''}\left(\eta_{i}\right)}{a(\phi )}\right]g^{T}gh\left(g,D_{i}\right)\\ & \frac{\partial^{2}l_{y,D}(\alpha ,\beta ,\phi )}{\partial \beta^{T}\partial \phi }=\sum_{i=1}^{N}\;\;\left(-{\left\{p_{\theta }\left(y_{i},D_{i}|x_{i}\right)\right\}}^{-2}\right)\left[\sum_{g\in 𝒢}\;\;f_{\theta }\left(y_{i}|x_{i},g\right)\frac{y_{i}-b^{'}\left(\eta_{i}\right)}{a(\phi )}g^{T}h\left(g,D_{i}\right)\right]\left\{\sum_{g\in 𝒢}\;\;f_{\theta }\left(y_{i}|x_{i},g\right)\left[-\frac{y_{i}\eta_{i}-b\left(\eta_{i}\right)}{[a(\phi ){]}^{2}}a^{'}(\phi )+\frac{\partial c\left(y_{i},\phi \right)}{\partial \phi }\right]h\left(g,D_{i}\right)\right\}\\ & \;+{\left\{p_{\theta }\left(y_{i},D_{i}|x_{i}\right)\right\}}^{-1}\sum_{g\in 𝒢}\;\;f_{\theta }\left(y_{i}|x_{i},g\right)\left(\left(\frac{y_{i}-b^{'}\left(\eta_{i}\right)}{a(\phi )}g^{T}\right)\left(-\frac{y_{i}\eta_{i}-b\left(\eta_{i}\right)}{[a(\phi ){]}^{2}}a^{'}(\phi )+\frac{\partial c\left(y_{i},\phi \right)}{\partial \phi }\right)-\left(\frac{y_{i}-b^{'}\left(\eta_{i}\right)}{[a(\phi ){]}^{2}}a^{'}(\phi )g^{T}\right)\right)h\left(g,D_{i}\right);\\ & \frac{\partial^{2}l_{y,D}(\alpha ,\beta ,\phi )}{\partial \phi^{2}}=\sum_{i=1}^{N}\;\;\left[-{\left\{p_{\theta }\left(y_{i},D_{i}|x_{i}\right)\right\}}^{-2}{\left(\sum_{g\in 𝒢}\;\;f_{\theta }\left(y_{i}|x_{i},g\right)\left[-\frac{y_{i}\eta_{i}-b\left(\eta_{i}\right)}{[a(\phi ){]}^{2}}a^{'}(\phi )+\frac{\partial c\left(y_{i},\phi \right)}{\partial \phi }\right]h\left(g,D_{i}\right)\right)}^{2}\right.\\ & \left.+{\left\{p_{\theta }\left(y_{i},D_{i}|x_{i}\right)\right\}}^{-1}\sum_{g\in 𝒢}\;\;f_{\theta }\left(y_{i}|x_{i},g\right)\left\{{\left[-\frac{y_{i}\eta_{i}-b\left(\eta_{i}\right)}{[a(\phi ){]}^{2}}a^{'}(\phi )+\frac{\partial c\left(y_{i},\phi \right)}{\partial \phi }\right]}^{2}+\left(y_{i}\eta_{i}-b\left(\eta_{i}\right)\right)\left(\frac{2{\left[a^{'}(\phi )\right]}^{2}}{[a(\phi ){]}^{3}}-\frac{a^{''}(\phi )}{[a(\phi ){]}^{2}}\right)+\frac{\partial^{2}c\left(y_{i},\phi \right)}{\partial \phi^{2}}\right\}h\left(g,D_{i}\right)\right];\\ & \frac{\partial^{2}l_{y,D}(\alpha ,\beta ,\phi )}{\partial \beta^{T}\partial \alpha }={\left(\frac{\partial^{2}l_{y,D}(\alpha ,\beta ,\phi )}{\partial \alpha^{T}\partial \beta }\right)}^{T};\;\frac{\partial^{2}l_{y,D}(\alpha ,\beta ,\phi )}{\partial \phi \partial \alpha }={\left(\frac{\partial^{2}l_{y,D}(\alpha ,\beta ,\phi )}{\partial \alpha^{T}\partial \phi }\right)}^{T};\;\frac{\partial^{2}l_{y,D}(\alpha ,\beta ,\phi )}{\partial \phi \partial \beta }={\left(\frac{\partial^{2}l_{y,D}(\alpha ,\beta ,\phi )}{\partial \beta^{T}\partial \phi }\right)}^{T}. \end{array}$$

In the following, denote the constrained maximum likelihood estimate of the parameters (α,β,ϕ) under the null hypothesis $H_{0}:\beta =0$ as $\tilde{\theta }=(\tilde{\alpha },0,\tilde{\phi })$. The score statistic is

(5) $$R(y,D)={\left[s_{y,D}(\tilde{\alpha },0,\tilde{\phi })\right]}^{T}{\left[o_{y,D}(\tilde{\alpha },0,\tilde{\phi })\right]}^{-1}\left[s_{y,D}(\tilde{\alpha },0,\tilde{\phi })\right].$$

To calculate this score statistic, we need to evaluate both the score function o(y,D)(α,β,ϕ) and the information matrix o(y,D)(α,β,ϕ) at the constrained MLE $\tilde{\theta }=(\tilde{\alpha },0,\tilde{\phi })$. The value of evaluating the score function at the constrained MLE under the null hypothesis, i.e. $\tilde{\theta }=(\tilde{\alpha },0,\tilde{\phi })$, is derived to be

$$s_{y,D}\left(\tilde{\alpha },0,\tilde{\phi }\right)=\left[\begin{array}{c} 0\\ \overset{N}{\underset{i=1}{\sum }}\frac{y_{i}-b^{\prime }\left(\tilde{\alpha }x_{i}^{T}\right)}{a\left(\tilde{\phi }\right)}\\ 0 \end{array}E\left(g^{T}|D_{i}\right)\right],$$

where $E\left(g^{T}|D_{i}\right)={\left\{\sum_{g\in 𝒢}\;h\left(g,D_{i}\right)\right\}}^{-1}\sum_{g\in 𝒢}\;g^{T}h\left(g,D_{i}\right)=\left(\sum_{g\in 𝒢}\;g^{T}h\left(g,D_{i}\right)\right)/\left(\sum_{g\in 𝒢}\;h\left(g,D_{i}\right)\right)$ is the posterior expectation of the genotype of individual i given sequencing data $D_{i}$. We have provided full derivation details in Appendix D.

The value of evaluating the observed information matrix at constrained MLE $\tilde{\theta }$ is derived to be the following. We have provided full derivation details in Appendix E.


$$\begin{array}{ll} & {\left.\frac{\partial^{2}l_{y,D}(\alpha ,\beta ,\phi )}{\partial \alpha^{T}\partial \alpha }\right|}_{\tilde{\theta }}=-\frac{1}{a(\tilde{\phi })}\sum_{i=1}^{N}\;\;b^{''}\left(\tilde{\alpha }x_{i}^{T}\right)x_{i}^{T}x_{i}\\ & {\left.\frac{\partial^{2}l_{y,D}(\alpha ,\beta ,\phi )}{\partial \alpha^{T}\partial \beta }\right|}_{\tilde{\theta }}=-\frac{1}{a(\tilde{\phi })}\sum_{i=1}^{N}\;\;b^{''}\left(\tilde{\alpha }x_{i}^{T}\right)x_{i}^{T}E\left(g|D_{i}\right)\\ & {\left.\frac{\partial^{2}l_{y,D}(\alpha ,\beta ,\phi )}{\partial \alpha^{T}\partial \phi }\right|}_{\tilde{\theta }}=0\\ & {\left.\frac{\partial^{2}l_{y,D}(\alpha ,\beta ,\phi )}{\partial \beta^{T}\partial \beta }\right|}_{\tilde{\theta }}=\sum_{i=1}^{N}\;\;\left[\frac{{\left(y_{i}-b^{'}\left(\tilde{\alpha }x_{i}^{T}\right)\right)}^{2}}{[a(\tilde{\phi }){]}^{2}}\left(E\left(g^{T}g|D_{i}\right)-E\left(g^{T}|D_{i}\right)E\left(g|D_{i}\right)\right)-\frac{b^{''}\left(\tilde{\alpha }x_{i}^{T}\right)}{a(\tilde{\phi })}E\left(g^{T}g|D_{i}\right)\right]\\ & {\left.\frac{\partial^{2}l_{y,D}(\alpha ,\beta ,\phi )}{\partial \beta^{T}\partial \phi }\right|}_{\tilde{\theta }}=-\frac{a^{'}(\tilde{\phi })}{[a(\tilde{\phi }){]}^{2}}\sum_{i=1}^{N}\;\;\left(y_{i}-b^{'}\left(\tilde{\alpha }x_{i}^{T}\right)\right)E\left(g^{T}|D_{i}\right)\\ & {\left.\frac{\partial^{2}l_{y,D}(\alpha ,\beta ,\phi )}{\partial \phi^{2}}\right|}_{\tilde{\theta }}=\sum_{i=1}^{N}\;\;\left[\left(y_{i}\tilde{\alpha }x_{i}^{T}-b\left(\tilde{\alpha }x_{i}^{T}\right)\right)\left(\frac{2{\left[a^{'}(\tilde{\phi })\right]}^{2}}{[a(\tilde{\phi }){]}^{3}}-\frac{a^{''}(\tilde{\phi })}{[a(\tilde{\phi }){]}^{2}}\right)+{\left.\frac{\partial^{2}c\left(y_{i},\phi \right)}{\partial \phi^{2}}\right|}_{\tilde{\theta }}\right]\\ & {\left.\frac{\partial^{2}l_{y,D}(\alpha ,\beta ,\phi )}{\partial \beta^{T}\partial \alpha }\right|}_{\tilde{\theta }}={\left({\left.\frac{\partial^{2}l_{y,D}(\alpha ,\beta ,\phi )}{\partial \alpha^{T}\partial \beta }\right|}_{\tilde{\theta }}\right)}^{T};{\left.\frac{\partial^{2}l_{y,D}(\alpha ,\beta ,\phi )}{\partial \phi \partial \alpha }\right|}_{\tilde{\theta }}={\left({\left.\frac{\partial^{2}l_{y,D}(\alpha ,\beta ,\phi )}{\partial \alpha^{T}\partial \phi }\right|}_{\tilde{\theta }}\right)}^{T};\\ & {\left.\frac{\partial^{2}l_{y,D}(\alpha ,\beta ,\phi )}{\partial \phi \partial \beta }\right|}_{\tilde{\theta }}={\left({\left.\frac{\partial^{2}l_{y,D}(\alpha ,\beta ,\phi )}{\partial \beta^{T}\partial \phi }\right|}_{\tilde{\theta }}\right)}^{T} \end{array}$$

Under the null hypothesis, R(y,D) is approximately distributed as a Chi-square random variable with degrees of freedom $d_{g}$. The score test is conducted based on score statistics R(y,D) and the P-value of the test is then calculated.

#### 2.3.2. The situation when parameters are (α,β)

When the parameters are (α,β), i.e. ϕ is dropped out, the score function is

(6) $$s_{y,D}(\alpha ,\beta )=\left[\begin{array}{l} \partial l_{y,D}(\alpha ,\beta )/\partial \alpha^{T}\\ \partial l_{y,D}(\alpha ,\beta )/\partial \beta^{T} \end{array}\right],$$

where α is a row vector of length $d_{x}+1$ and β is a row vector of length $d_{g}$. The analytical formula of the score function is derived to be

$$s_{y,D}(\alpha ,\beta )=\sum_{i=1}^{N}\;\left[{\left\{p_{\theta }\left(y_{i},D_{i}|x_{i}\right)\right\}}^{-1}\sum_{g\in 𝒢}\;\;f_{\theta }\left(y_{i}|x_{i},g\right)\left[\begin{array}{l} \frac{y_{i}-b^{'}\left(\eta_{i}\right)}{a}x_{i}^{T}\\ \frac{y_{i}-b^{'}\left(\eta_{i}\right)}{a}g^{T} \end{array}\right]h\left(g,D_{i}\right)\right],$$

where $\eta_{i}=\eta \left(x_{i},g\right)=\alpha x_{i}^{T}+\beta g^{T}$. We have provided full derivation details in Appendix F.

The observed information matrix is

$$o_{y,D}(\alpha ,\beta )=-\left[\begin{array}{ll} \partial^{2}l_{y,D}(\alpha ,\beta )/\partial \alpha^{T}\partial \alpha & \partial^{2}l_{y,D}(\alpha ,\beta )/\partial \alpha^{T}\partial \beta \\ \partial^{2}l_{y,D}(\alpha ,\beta )/\partial \beta^{T}\partial \alpha & \partial^{2}l_{y,D}(\alpha ,\beta )/\partial \beta^{T}\partial \beta \end{array}\right].$$

with analytical formulae derived to be the following and full derivation details provided in Appendix G.


$$\begin{array}{ll} \frac{\partial^{2}l_{y,D}(\alpha ,\beta )}{\partial \alpha^{T}\partial \alpha }= & \;\sum_{i=1}^{N}\;\;\left(-{\left\{p_{\theta }\left(y_{i},D_{i}|x_{i}\right)\right\}}^{-2}\right)\left[\sum_{g\in 𝒢}\;\;f_{\theta }\left(y_{i}|x_{i},g\right)\frac{y_{i}-b^{'}\left(\eta_{i}\right)}{a}x_{i}^{T}h\left(g,D_{i}\right)\right]\left[\sum_{g\in 𝒢}\;\;f_{\theta }\left(y_{i}|x_{i},g\right)\frac{y_{i}-b^{'}\left(\eta_{i}\right)}{a}x_{i}h\left(g,D_{i}\right)\right]\\ & \;+{\left\{p_{\theta }\left(y_{i},D_{i}|x_{i}\right)\right\}}^{-1}\sum_{g\in 𝒢}\;\;f_{\theta }\left(y_{i}|x_{i},g\right)\left[\frac{{\left(y_{i}-b^{'}\left(\eta_{i}\right)\right)}^{2}}{a^{2}}-\frac{b^{''}\left(\eta_{i}\right)}{a}\right]x_{i}^{T}x_{i}h\left(g,D_{i}\right);\\ \frac{\partial^{2}l_{y,D}(\alpha ,\beta )}{\partial \alpha^{T}\partial \beta }= & \;\sum_{i=1}^{N}\;\;\left(-{\left\{p_{\theta }\left(y_{i},D_{i}|x_{i}\right)\right\}}^{-2}\right)\left[\sum_{g\in 𝒢}\;\;f_{\theta }\left(y_{i}|x_{i},g\right)\frac{y_{i}-b^{'}\left(\eta_{i}\right)}{a}x_{i}^{T}h\left(g,D_{i}\right)\right]\left[\sum_{g\in 𝒢}\;\;f_{\theta }\left(y_{i}|x_{i},g\right)\frac{y_{i}-b^{'}\left(\eta_{i}\right)}{a}gh\left(g,D_{i}\right)\right]\\ & \;+{\left\{p_{\theta }\left(y_{i},D_{i}|x_{i}\right)\right\}}^{-1}\sum_{g\in 𝒢}\;\;f_{\theta }\left(y_{i}|x_{i},g\right)\left[\frac{{\left(y_{i}-b^{'}\left(\eta_{i}\right)\right)}^{2}}{a^{2}}-\frac{b^{''}\left(\eta_{i}\right)}{a}\right]x_{i}^{T}gh\left(g,D_{i}\right);\\ \frac{\partial^{2}l_{y,D}(\alpha ,\beta )}{\partial \beta^{T}\partial \beta }= & \;\sum_{i=1}^{N}\;\;\left(-{\left\{p_{\theta }\left(y_{i},D_{i}|x_{i}\right)\right\}}^{-2}\right)\left[\sum_{g\in 𝒢}\;\;f_{\theta }\left(y_{i}|x_{i},g\right)\frac{y_{i}-b^{'}\left(\eta_{i}\right)}{a}g^{T}h\left(g,D_{i}\right)\right]\left[\sum_{g\in 𝒢}\;\;f_{\theta }\left(y_{i}|x_{i},g\right)\frac{y_{i}-b^{'}\left(\eta_{i}\right)}{a}gh\left(g,D_{i}\right)\right]\\ & \;+{\left\{p_{\theta }\left(y_{i},D_{i}|x_{i}\right)\right\}}^{-1}\sum_{g\in 𝒢}\;\;f_{\theta }\left(y_{i}|x_{i},g\right)\left[\frac{{\left(y_{i}-b^{'}\left(\eta_{i}\right)\right)}^{2}}{a^{2}}-\frac{b^{''}\left(\eta_{i}\right)}{a}\right]g^{T}gh\left(g,D_{i}\right);\\ \frac{\partial^{2}l_{y,D}(\alpha ,\beta )}{\partial \beta^{T}\partial \alpha }= & {\left(\frac{\partial^{2}l_{y,D}(\alpha ,\beta )}{\partial \alpha^{T}\partial \beta }\right)}^{T}. \end{array}$$

In the following, denote the constrained maximum likelihood estimate of the parameters (α,β) under the null hypothesis $H_{0}:\beta =0$ as $\tilde{\theta }=(\tilde{\alpha },0)$. The score statistic is

(7) $$R(y,D)={\left[s_{y,D}(\tilde{\alpha },0)\right]}^{T}{\left[o_{y,D}(\tilde{\alpha },0)\right]}^{-1}\left[s_{y,D}(\tilde{\alpha },0)\right].$$

To calculate this score statistic, we need to evaluate both the score function $s_{y,D}(\alpha ,\beta )$ and the information matrix o(y,D)(α,β) at the constrained MLE. The value of evaluating the score function at the constrained MLE under the null hypothesis, i.e. $\tilde{\theta }=(\tilde{\alpha },0)$, is derived to be

$$s_{y,D}(\tilde{\alpha },0)=\left[\begin{array}{c} 0\\ \sum_{i=1}^{N}\;\;\frac{y_{i}-b^{'}\left(\tilde{\alpha }x_{i}^{T}\right)}{a}E\left(g^{T}|D_{i}\right) \end{array}\right],$$

where $E\left(g^{T}|D_{i}\right)={\left\{\sum_{g\in 𝒢}\;h\left(g,D_{i}\right)\right\}}^{-1}\sum_{g\in 𝒢}\;g^{T}h\left(g,D_{i}\right)=\left(\sum_{g\in 𝒢}\;g^{T}h\left(g,D_{i}\right)\right)/\left(\sum_{g\in 𝒢}\;h\left(g,D_{i}\right)\right)$ is the posterior expectation of the genotype of individual i given sequencing data $D_{i}$. We have provided full derivation details in Appendix H.

The value of evaluating the information matrix at constrained MLE $\tilde{\theta }$ is derived to be the following with full derivation details provided in Appendix I.


$$\begin{array}{ll} & {\left.\frac{\partial^{2}l_{y,D}(\alpha ,\beta )}{\partial \alpha^{T}\partial \alpha }\right|}_{\tilde{\theta }}=-\frac{1}{a}\sum_{i=1}^{N}\;\;b^{''}\left(\tilde{\alpha }x_{i}^{T}\right)x_{i}^{T}x_{i};{\left.\frac{\partial^{2}l_{y,D}(\alpha ,\beta )}{\partial \alpha^{T}\partial \beta }\right|}_{\tilde{\theta }}=-\frac{1}{a}\sum_{i=1}^{N}\;\;b^{''}\left(\tilde{\alpha }x_{i}^{T}\right)x_{i}^{T}E\left(g|D_{i}\right)\\ & {\left.\frac{\partial^{2}l_{y,D}(\alpha ,\beta )}{\partial \beta^{T}\partial \beta }\right|}_{\tilde{\theta }}=\sum_{i=1}^{N}\;\;\left[\frac{{\left(y_{i}-b^{'}\left(\tilde{\alpha }x_{i}^{T}\right)\right)}^{2}}{a^{2}}\left(E\left(g^{T}g|D_{i}\right)-E\left(g^{T}|D_{i}\right)E\left(g|D_{i}\right)\right)-\frac{b^{''}\left(\tilde{\alpha }x_{i}^{T}\right)}{a}E\left(g^{T}g|D_{i}\right)\right]\\ & {\left.\frac{\partial^{2}l_{y,D}(\alpha ,\beta )}{\partial \beta^{T}\partial \alpha }\right|}_{\tilde{\theta }}={\left({\left.\frac{\partial^{2}l_{y,D}(\alpha ,\beta )}{\partial \alpha^{T}\partial \beta }\right|}_{\tilde{\theta }}\right)}^{T}. \end{array}$$

Under the null hypothesis, R(y,D) is approximately distributed as a Chi-square random variable with degrees of freedom $d_{g}$. The score test is conducted based on score statistics R(y,D) and the P-value of the test is then calculated.

### 2.4. Variable Collapse Test for a Group of Rare Genetic Variants

#### 2.4.1. The situation when parameters are (α,β,ϕ)

Variable collapse testing methods of rare variants combines individual genetic effects into an aggregate overall effect in the testing [22,35,36]. Different variable collapse methods use different ways to aggregate rare variants. In the genotype-based weighted burden test, the variable aggregating p rare variants $AG_{i}=\sum_{j=1}^{p}\;w_{j}g_{ij}$, where $g_{ij}$ is the genotype at rare variant j for individual i with allele coding of 1 as the rare allele and 0 as the wild or reference allele. The term $w_{j}$ is the weight. The equal weight $\left(w_{1}=w_{2}=\cdots =w_{p}=1\right)$ means that $AG_{i}=\sum_{j=1}^{p}\;g_{ij}$ is the sum of rare alleles in all the rare variants in the group.

The use of $AG_{i}$ in the linear model means that researchers assume that the influence of rare variants $g_{ij}$ on phenotype $y_{i}$ is through the aggregate variable $AG_{i}$.

We model a complex phenotype using a generalized linear model [31,32]. For individual i, the same generalized linear model is used except the change in linear predictor $\eta_{i}$. The probability of observing phenotype $y_{i}$ is modelled to be

(8) $$p\left(y_{i}|x_{i},g_{i}\right)=p_{\alpha ,\beta_{0},\phi }\left(y_{i}|x_{i},g_{i}\right)=exp\left(\frac{y_{i}\eta_{i}-b\left(\eta_{i}\right)}{a(\phi )}+c\left(y_{i},\phi \right)\right),$$

where the row vector $\alpha \in {ℛ}^{d_{x}+1}$, the scalar $\beta_{0}\in ℛ$, and the linear predictor

(9) $$\eta_{i}=\eta_{\alpha ,\beta_{0}}\left(x_{i},g_{i}\right)=\alpha x_{i}^{T}+\beta_{0}AG_{i}=\alpha x_{i}^{T}+\beta_{0}\sum_{j=1}^{d_{g}}\;w_{j}g_{ij}$$

with appropriate choice of the function a(.),.b(.) and c(.,.), depending on the type of the phenotype under consideration. Note that the aggregate genetic variable $AG_{i}=\sum_{j=1}^{d_{g}}\;w_{j}g_{ij}$ aggregates or collapses or combines $d_{g}$ rare genetic variables into one aggregate variable as the linear combination of the $d_{g}$ rare genetic values. The distribution of the phenotype $y_{i}$ depends on the individual predictors $\left(x_{i}\right.$ and $\left.AG_{i}\right)$ as well as the parameters $\left(\alpha ,\beta_{0},\phi \right)$. For rare variants, rare allele is typically coded as 1 and wild allele is coded as 0.

Consider the model we specified before for a group of common variants. That is the same GLM model except the change in linear predictor $\eta_{i}$, i.e.

(10) $$\eta_{i}=\alpha x_{i}^{T}+\beta g_{i}^{T},$$

where $\beta \in {ℛ}^{d_{g}}$. We found there is a connection between the effects of the two models. This allows us to use *the chain rule in calculus* to easily derive the likelihood function, score function, observed information matrix, and evaluations of these functions at constrained MLE for rare variant model, i.e. Equation (9) based on what we have derived before based on Equation (10).

To be more specific, since the effects of rare variants as modeled by $\beta_{0}$ satisfy the condition that $\beta =\beta_{0}W$, where $\beta \in {ℛ}^{d_{g}}$ are the effects of $d_{g}$ rare variants and $\beta_{0}\in ℛ$. For example, when $w_{1}=w_{2}=\cdots =w_{d_{g}}=1$, we have W=[1,1,…,1], which is a unit row vector of length $d_{g}$. In this situation, we have $AG_{i}=\sum_{j=1}^{d_{g}}\;g_{ij}$ [22,35]. In the linear model framework, we have $\eta_{i}=\alpha x_{i}^{T}+\beta_{0}Wg_{i}^{T}=\alpha x_{i}^{T}+\beta_{0}\sum_{j=1}^{d_{g}}\;w_{j}g_{ij}$. Other weights may also be used to collapse rare variants such as $w_{j}=\beta_{0}f_{\text{Beta}}\left(MAF_{j},1,25\right)$, where $f_{\text{Beta}}$ is the Beta density function, and $MAF_{j}$ is the minor allele frequency of rare variant j and $\beta_{0}$ is the common factor of all weights [1,23,24].

In our NGS data-based variable collapse method (VC), we use the same assumption about weights as used in most genotype-based variable-collapse rare variant testing methods in the literature, i.e. the weighted burden test assumption[22,35]. We model $\beta =\beta_{0}W$, where the row vector $W=\left(w_{1},w_{2},\ldots ,w_{d_{g}}\right)$ is the weight and $\beta_{0}$ is a common factor. For the purpose of identification, we impose the constraint $\sum_{j=1}^{d_{g}}\;w_{j}=d_{g}$.

We use the same generalized linear model framework as before. In our previous *joint significance testing method* for a group of *common genetic variants*, the linear predictor $\eta_{i}$ is modelled as $\eta_{i}=\eta_{\alpha ,\beta }\left(x_{i},g_{i}\right)=\alpha x_{i}^{T}+\beta g_{i}^{T}$ and the parameters are (α,β,ϕ), where β is a row vector of length $d_{g}$. In comparison, in our *variable collapse testing method* for a group of *rare genetic variants*, the linear predictor $\eta_{i}$ is modeled as $\eta_{i}=\eta_{\alpha ,\beta }\left(x_{i},g_{i}\right)=\alpha x_{i}^{T}+\beta_{0}Wg_{i}^{T}$ and the parameters are $\left(\alpha ,\beta_{0},\phi \right)$, where $\beta_{0}$ is a scalar. First, under the null hypothesis $H_{0}:\beta =0$ or $H_{0}:\beta_{0}=0$, we will get the same constrained MLE for α and ϕ, no matter which log-likelihood function $\left(l_{y,D}(\alpha ,\beta ,\phi )\right.$ or $\left.l_{y,D}\left(\alpha ,\beta_{0},\phi \right)\right)$ we maximize. Thus, we can use the same notation $\tilde{\theta }=(\tilde{\alpha },0,\tilde{\phi })$ to represent both the constrained MLE in $l_{y,D}(\alpha ,\beta ,\phi )$ (note the term 0 in this constrained MLE is a zero row vector of length $d_{g}$) and the constrained MLE in $l_{y,D}\left(\alpha ,\beta_{0},\phi \right)$ (note the term 0 in this constrained MLE is a scalar of 0).

By the chain rule in calculus, the score function evaluated at the constrained MLE is derived as the following.

$${\left.\frac{\partial l_{y,D}\left(\alpha ,\beta_{0},\phi \right)}{\partial \beta_{0}}\right|}_{\tilde{\theta }}={\left.W\frac{\partial l_{y,D}(\alpha ,\beta ,\phi )}{\partial \beta }\right|}_{\tilde{\theta }}=W\left\{\frac{1}{a(\tilde{\phi })}\sum_{i=1}^{N}\;\;\left[y_{i}-b^{'}\left(\tilde{\alpha }x_{i}^{T}\right)\right]E\left(g^{T}|D_{i}\right)\right\}\;{\left.\frac{\partial l_{y,D}\left(\alpha ,\beta_{0},\phi \right)}{\partial \alpha }\right|}_{\tilde{\theta }}=0;{\left.\frac{\partial l_{y,D}\left(\alpha ,\beta_{0},\phi \right)}{\partial \phi }\right|}_{\tilde{\theta }}=0$$

where $E\left(g^{T}|D_{i}\right)={\left\{\sum_{g\in 𝒢}\;h\left(g,D_{i}\right)\right\}}^{-1}\sum_{g\in 𝒢}\;g^{T}h\left(g,D_{i}\right)=\left(\sum_{g\in 𝒢}\;g^{T}h\left(g,D_{i}\right)\right)/\left(\sum_{g\in 𝒢}\;h\left(g,D_{i})\right)\right.$ is the posterior expectation of the genotype of individual i given sequencing data $D_{i}$. Note that the evaluation of the last two score functions at the constrained MLE is equal to 0, because the constrained MLE maximizes the log-likelihood under the null hypothesis $H_{0}:\beta_{0}=0$, so that the derivatives of the log-likelihood with respect to α and ϕ are equal to 0 when being evaluated at constrained MLE, according to the first order condition in constrained optimization.

Working similarly and applying the chain rule in calculus, we can derive the observed information matrix, and evaluate the observed information matrix at the constrained MLE. Based on the chain rule, we have

$${\left.\frac{\partial^{2}l_{y,D}\left(\alpha ,\beta_{0},\phi \right)}{\partial \alpha^{T}\partial \alpha }\right|}_{\tilde{\theta }}={\left.\frac{\partial^{2}l_{y,D}(\alpha ,\beta ,\phi )}{\partial \alpha^{T}\partial \alpha }\right|}_{\tilde{\theta }}=-\frac{1}{a(\tilde{\phi })}\sum_{i=1}^{N}\;\;b^{''}\left(\tilde{\alpha }x_{i}^{T}\right)x_{i}^{T}x_{i}\;{\left.\frac{\partial^{2}l_{y,D}\left(\alpha ,\beta_{0},\phi \right)}{\partial \beta_{0}^{2}}\right|}_{\tilde{\theta }}=W\left\{{\left.\frac{\partial^{2}l_{y,D}(\alpha ,\beta ,\phi )}{\partial \beta^{T}\partial \beta }\right|}_{\tilde{\theta }}\right\}W^{T}\;\;=W\left\{\sum_{i=1}^{N}\;\;\left[\frac{{\left(y_{i}-b^{'}\left(\tilde{\alpha }x_{i}^{T}\right)\right)}^{2}}{[a(\tilde{\phi }){]}^{2}}\left(E\left(g^{T}g|D_{i}\right)-E\left(g^{T}|D_{i}\right)E\left(g|D_{i}\right)\right)-\frac{b^{''}\left(\tilde{\alpha }x_{i}^{T}\right)}{a(\tilde{\phi })}E\left(g^{T}g|D_{i}\right)\right]\right.\;{\left.\frac{\partial^{2}l_{y,D}\left(\alpha ,\beta_{0},\phi \right)}{\partial \phi^{2}}\right|}_{\tilde{\theta }}=\sum_{i=1}^{N}\;\;\left[\left(y_{i}\tilde{\alpha }x_{i}^{T}-b\left(\tilde{\alpha }x_{i}^{T}\right)\right)\left(\frac{2{\left[a^{'}(\tilde{\phi })\right]}^{2}}{[a(\tilde{\phi }){]}^{3}}-\frac{a^{''}(\tilde{\phi })}{[a(\tilde{\phi }){]}^{2}}\right)+{\left.\frac{\partial^{2}c\left(y_{i},\phi \right)}{\partial \phi^{2}}\right|}_{\tilde{\theta }}\right]\;{\left.\frac{\partial^{2}l_{y_{,}D}\left(\alpha ,\beta_{0},\phi \right)}{\partial \alpha^{T}\partial \beta_{0}}\right|}_{\tilde{\theta }}={\left.\frac{\partial^{2}l_{y,D}(\alpha ,\beta ,\phi )}{\partial \alpha^{T}\partial \beta }\right|}_{\tilde{\theta }}W^{T}=-\frac{1}{a(\tilde{\phi })}\sum_{i=1}^{N}\;\;b^{''}\left(\tilde{\alpha }x_{i}^{T}\right)x_{i}^{T}E\left(g|D_{i}\right)W^{T}\;{\left.\frac{\partial^{2}l_{y,D}\left(\alpha ,\beta_{0},\phi \right)}{\partial \alpha^{T}\partial \phi }\right|}_{\tilde{\theta }}={\left.\frac{\partial^{2}l_{y,D}(\alpha ,\beta ,\phi )}{\partial \alpha^{T}\partial \phi }\right|}_{\tilde{\theta }}=0\;{\left.\frac{\partial^{2}l_{y,D}\left(\alpha ,\beta_{0},\phi \right)}{\partial \beta_{0}\partial \phi }\right|}_{\tilde{\theta }}={\left.W\frac{\partial^{2}l_{y,D}(\alpha ,\beta ,\phi )}{\partial \beta^{T}\partial \phi }\right|}_{\tilde{\theta }}=-W\frac{a^{'}(\tilde{\phi })}{[a(\tilde{\phi }){]}^{2}}\sum_{i=1}^{N}\;\;\left(y_{i}-b^{'}\left(\tilde{\alpha }x_{i}^{T}\right)\right)E\left(g^{T}|D_{i}\right)\;{\left.\frac{\partial^{2}l_{y,D}\left(\alpha ,\beta_{0},\phi \right)}{\partial \beta_{0}\partial \alpha }\right|}_{\tilde{\theta }}={\left({\left.\frac{\partial^{2}l_{y,D}\left(\alpha ,\beta_{0},\phi \right)}{\partial \alpha^{T}\partial \beta_{0}}\right|}_{\tilde{\theta }}\right)}^{T};{\left.\frac{\partial^{2}l_{y,D}\left(\alpha ,\beta_{0},\phi \right)}{\partial \phi \partial \alpha }\right|}_{\tilde{\theta }}={\left({\left.\frac{\partial^{2}l_{y,D}\left(\alpha ,\beta_{0},\phi \right)}{\partial \alpha^{T}\partial \phi }\right|}_{\tilde{\theta }}\right)}^{T}\;{\left.\frac{\partial^{2}l_{y,D}\left(\alpha ,\beta_{0},\phi \right)}{\partial \phi \partial \beta_{0}}\right|}_{\tilde{\theta }}={\left.\frac{\partial^{2}l_{y,D}\left(\alpha ,\beta_{0},\phi \right)}{\partial \beta_{0}\partial \phi }\right|}_{\tilde{\theta }}$$

All the formulae except the last three are derived by the chain rule in calculus. The last three formulae are obtained by matrix transposition.

A chi-square statistic is derived and used in the test. The score statistic is

$$R(y,D)={\left[s_{y,D}(\tilde{\alpha },0,\tilde{\phi })\right]}^{T}o_{y,D}(\tilde{\alpha },0,\tilde{\phi })\left[s_{y,D}(\tilde{\alpha },0,\tilde{\phi })\right].$$

Under the null hypothesis $H_{0}:\beta_{0}=0,R(y,D)$ is approximately distributed as a Chi-square random variable with 1 degree of freedom. The score test is conducted based on score statistics R(y,D) and the P-value is calculated.

#### 2.4.2. The situation when parameters are (α,β)

Consider the situation when ϕ is dropped out and parameters are parameters are (α,β). All setups are the same except the following changes:

1. the parameters in rare-variant testing are $\left(\alpha ,\beta_{0}\right)$, where $\beta_{0}\in ℛ$ and the parameters in common-variant testing are (α,β), where $\beta \in {ℛ}^{d_{g}}$;
2. the likelihood functions for rare-variant testing and common-variant testing are *respectively* denoted as $l_{y,D}\left(\alpha ,\beta_{0}\right)$ and $l_{y,D}(\alpha ,\beta )$;
3. use the same notation $\tilde{\theta }=(\tilde{\alpha },0)$ to represent the constrained MLE in $l_{y,D}\left(\alpha ,\beta_{0}\right)$ (note the term 0 in the constrained MLE is a scalar of 0) and the constrained MLE in $l_{y,D}(\alpha ,\beta )$ (note the term 0 in the constrained MLE is a zero row vector of length $d_{g}$).

The score function evaluated at the constrained MLE is derived as the following.

$${\left.\frac{\partial l_{y,D}\left(\alpha ,\beta_{0}\right)}{\partial \beta_{0}}\right|}_{\tilde{\theta }}={\left.W\frac{\partial l_{y,D}(\alpha ,\beta )}{\partial \beta_{0}}\right|}_{\tilde{\theta }}=W\left\{a^{-1}\sum_{i=1}^{N}\;\;\left(y_{i}-\tilde{\alpha }x_{i}^{T}\right)E\left(g^{T}|D_{i}\right)\right\}\;{\left.\frac{\partial l_{y,D}\left(\alpha ,\beta_{0}\right)}{\partial \alpha }\right|}_{\tilde{\theta }}=0$$

Note that on the above, the first formula is derived by the chain rule in calculus. The second formula is derived to be 0 because the constrained MLE maximizes the log-likelihood under the null hypothesis $H_{0}:\beta_{0}=0$, so that the derivative of the log-likelihood with respect to α is equal to 0 when being evaluated at the constrained MLE according to the first-order condition of constrained optimization.

Working similarly, we can derive the observed information matrix, and evaluate the observed information matrix at the constrained MLE. We have the following derivations:

$$\begin{array}{ll} {\left.\frac{\partial^{2}l_{y,D}\left(\alpha ,\beta_{0}\right)}{\partial \alpha^{T}\partial \alpha }\right|}_{\tilde{\theta }} & \;={\left.\frac{\partial^{2}l_{y,D}(\alpha ,\beta )}{\partial \alpha^{T}\partial \alpha }\right|}_{\tilde{\theta }}=-\frac{1}{a}\sum_{i=1}^{N}\;\;b^{''}\left(\tilde{\alpha }x_{i}^{T}\right)x_{i}^{T}x_{i}\\ {\left.\frac{\partial^{2}l_{y,D}\left(\alpha ,\beta_{0}\right)}{\partial \beta_{0}^{2}}\right|}_{\tilde{\theta }} & \;=W\left\{{\left.\frac{\partial^{2}l_{y,D}(\alpha ,\beta )}{\partial \beta^{T}\partial \beta }\right|}_{\tilde{\theta }}\right\}W^{T}\\ & \;=W\left\{\sum_{i=1}^{N}\;\;\left[\frac{{\left(y_{i}-b^{'}\left(\tilde{\alpha }x_{i}^{T}\right)\right)}^{2}}{a^{2}}\left(E\left(g^{T}g|D_{i}\right)-E\left(g^{T}|D_{i}\right)E\left(g|D_{i}\right)\right)-\frac{b^{''}\left(\tilde{\alpha }x_{i}^{T}\right)}{a}E\left(g^{T}g|D_{i}\right)\right]\right\}W^{T}\\ {\left.\frac{\partial^{2}l_{y,D}\left(\alpha ,\beta_{0}\right)}{\partial \alpha^{T}\partial \beta_{0}}\right|}_{\tilde{\theta }} & \;={\left.\frac{\partial^{2}l_{y,D}(\alpha ,\beta )}{\partial \alpha^{T}\partial \beta }\right|}_{\tilde{\theta }}W^{T}=-\frac{1}{a}\left\{\sum_{i=1}^{N}\;\;b^{''}\left(\tilde{\alpha }x_{i}^{T}\right)x_{i}^{T}E\left(g|D_{i}\right)\right\}W^{T}\\ {\left.\frac{\partial^{2}l_{y,D}\left(\alpha ,\beta_{0}\right)}{\partial \beta_{0}\partial \alpha }\right|}_{\tilde{\theta }} & \;={\left({\left.\frac{\partial^{2}l_{y,D}\left(\alpha ,\beta_{0}\right)}{\partial \alpha^{T}\partial \beta_{0}}\right|}_{\tilde{\theta }}\right)}^{T}; \end{array}$$

The first three formulae are derived by the chain rule in calculus. The last formula is obtained by matrix transposition.

A chi-square statistic is derived and used in the test. The score statistic is

$$R(y,D)={\left[s_{y,D}(\tilde{\alpha },0)\right]}^{T}o_{y,D}(\tilde{\alpha },0)\left[s_{y,D}(\tilde{\alpha },0)\right].$$

Under the null hypothesis $H_{0}:\beta_{0}=0,R(y,D)$ is approximately distributed as a Chi-square random variable with 1 degree of freedom. The score test is conducted based on score statistics R(y,D) and the P-value is calculated.

## 3. Results of Simulation Studies

We perform extensive simulation studies under a range of settings to evaluate the performance of our proposed sequencing data-based methods (*joint significance test* for a group of *common variants* and *variable collapse test* for a group of *rare variants*) and their corresponding genotype-based methods in the literature.

For common genetic variants and binary response, we compare our NGS data-based joint significance test (JS) with the corresponding genotype-based methods (Chi-square test) in the literature. For rare genetic variants and binary response, we compare our sequencing data-based variable collapse test (VC) with the genotype-based burden test and the genotype-based SKAT test in the literature.

We also conducted simulations for count/integer response. Although continuous response and binary response are two mostly encountered types of phenotype in association studies, there are other types of phenotypes which are not as common as continous type and binary type. We show our methods for count/integer phenotype to show that the use of a generalized linear model-framework allows us to handle other types of responses in addition to continuous phenotype. Because SKAT testing is only available for continuous phenotype and binary phenotype[23], we will not compare our methods with SKAT testing for count/integer response in rare-variant testing. For count/integer phenotype and rare genetic variants, we compare our sequencing data-based variable collapse test (VC) with the genotype-based burden test in the literature. For count/integer phenotype and common genetic variants, we compare our methods NGS data-based joint significance test (JS) with the corresponding genotype-based methods (Chi-square test) in the literature

We use COSI software’s bestfit model to generate 100-kb regions that mimic LD patterns, local recombination rate and population history of Europeans through a coalescent model [37]. Within the simulated regions, chromosomes are generated. Sequencing data are simulated using the software ShotGun [38] with a per base pair error rate of 0.5%. ShotGun is available via the link https://yunliweb.its.unc.edu/shotgun.html. We consider a wide range of sequencing depth scenarios by choosing average sequencing depths to be 1X, 2X, 4X and 10X respectively. Rare variants and common variants are separated depending on whether their MAFs are above or equal to 0.05. We generate two additional covariates in our simulation: a binary covariate $X_{1}\sim Bernoulli(0.5)$ and a continuous covariate $X_{2}\sim N(0,1)$.

The binary phenotype is generated via a logistics regression model:

$$ln\left(\frac{P(Y=1)}{1-P(Y=1)}\right)=\beta_{0}+\beta_{1}X_{1}+\beta_{2}X_{2}+\sum_{j=1}^{d_{g}}\;\beta_{gj}G_{j}+\epsilon ,$$

where $\epsilon \sim N(0,1),\beta_{0}=0,\beta_{1}=1,\beta_{2}=1$, and $d_{g}$ is the number of genetic variables.

The count/integer phenotype is generated via a Poisson regression model:

(12) $$Y\sim Poisson\left(\lambda =e^{\eta }\right),\eta =\beta_{0}+\beta_{1}X_{1}+\beta_{2}X_{2}+\sum_{j=1}^{d_{g}}\;\beta_{gj}G_{j},$$

where $\epsilon \sim N(0,1),\beta_{0}=0,\beta_{1}=1,\beta_{2}=1$, and $d_{g}$ is the number of genetic variables.

In simulations of both types, the values of $\beta_{gj}$, i.e. genetic effects, are set differently in different scenarios so that we can evaluate Type I error and conduct power analysis.

Under the null hypothesis, i.e. $\beta_{g1}=\beta_{g1}=\cdots =\beta_{gd_{g}}=0,\;9000$ replicates are generated to evaluate Type I errors. We evaluate Type I errors for all possible combinations of three sample sizes and four sequencing depths. The three sample sizes are 300, 500, and 1000. The four sequencing depths are 1X, 2X, 4X, and 10X. We reported the Type I error results for (1) the *joint significance test* of a group of *common genetic variants* with a continuous phenotype and (2) the *variable collapse test* of a group of *rare genetic variants* with a continuous phenotype.

We next consider the alternative hypothesis, i.e. there are some non-zero genetic effects for the $d_{g}$ genetic variants. Under the alternative hypothesis, we randomly choose a group of genetic markers as causal markers in simulating the phenotype. We first generate a random integer number between two and five as the number of *causal common variants*, and a random integer between two and ten as the number of *causal rare variants*. Then we use these causal genetic variants and our simulated additional covariates $\left(X_{1},X_{2}\right)$ to simulate continuous phenotypes. We let the total genetic effective size be between 0 and 1 (Scale Parameter = 0.2 multiplied by the magnitude range of 0 to 5). The individual effect is the total effect divided by the number of causal variants in each simulation.

**Figure 1.**
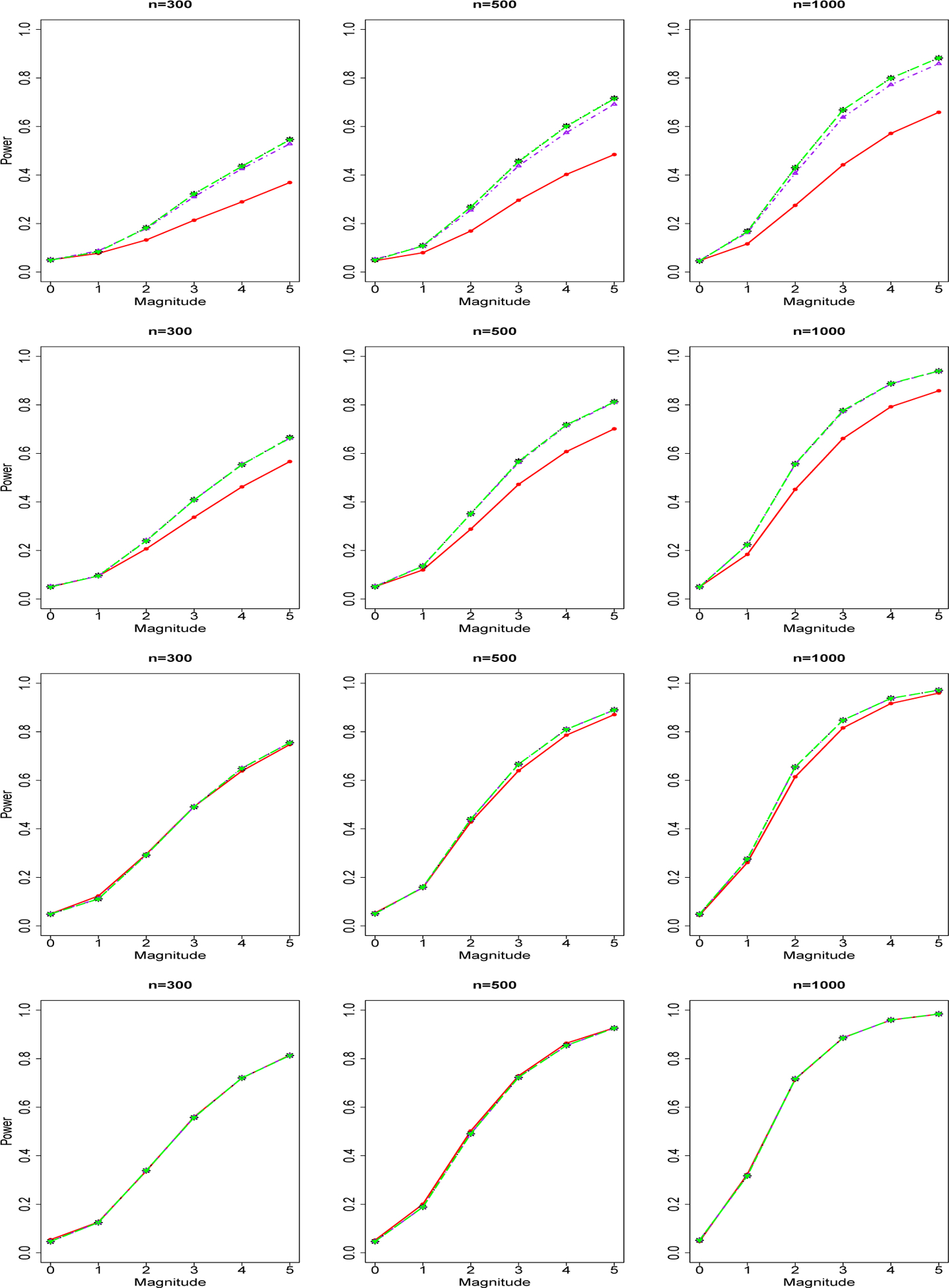
Power Curves of Tests for A Group of Common Genetic Variants and Binary Phenotype. The four rows of images from up to down are for sequencing depth 1X, 2X, 4X, 10X. The three columns of images from left to right are for sample size n=300,500,1000. Powers of genotype-based Chi-square test (red solid line), NGS data-based joint significance test 1 (black dotted line), test 2 (purple dotted-dashed line), and test 3 (green long-dashed line).

**Figure 2.**
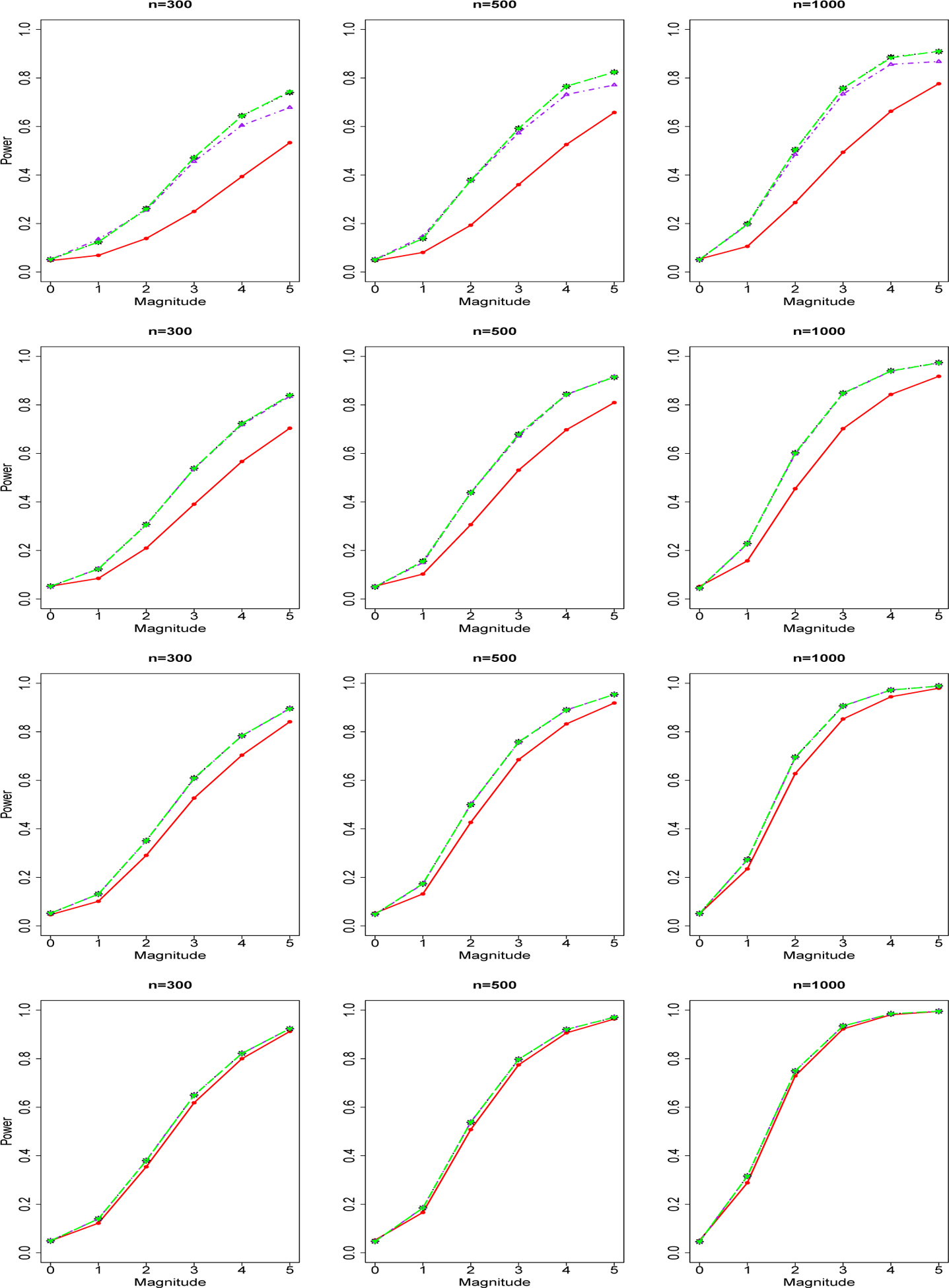
Power Curves of Tests for A Group of Common Genetic Variants and Count/Integer Phenotype. The four rows of images from up to down are for sequencing depth 1X, 2X, 4X, 10X. The three columns of images from left to right are for sample size n=300,500,1000. Powers of genotype-based Chi-square test (red solid line), NGS data-based joint significance test 1 (black dotted line), test 2 (purple dotted-dashed line), and test 3 (green long-dashed line).

**Figure 3.**
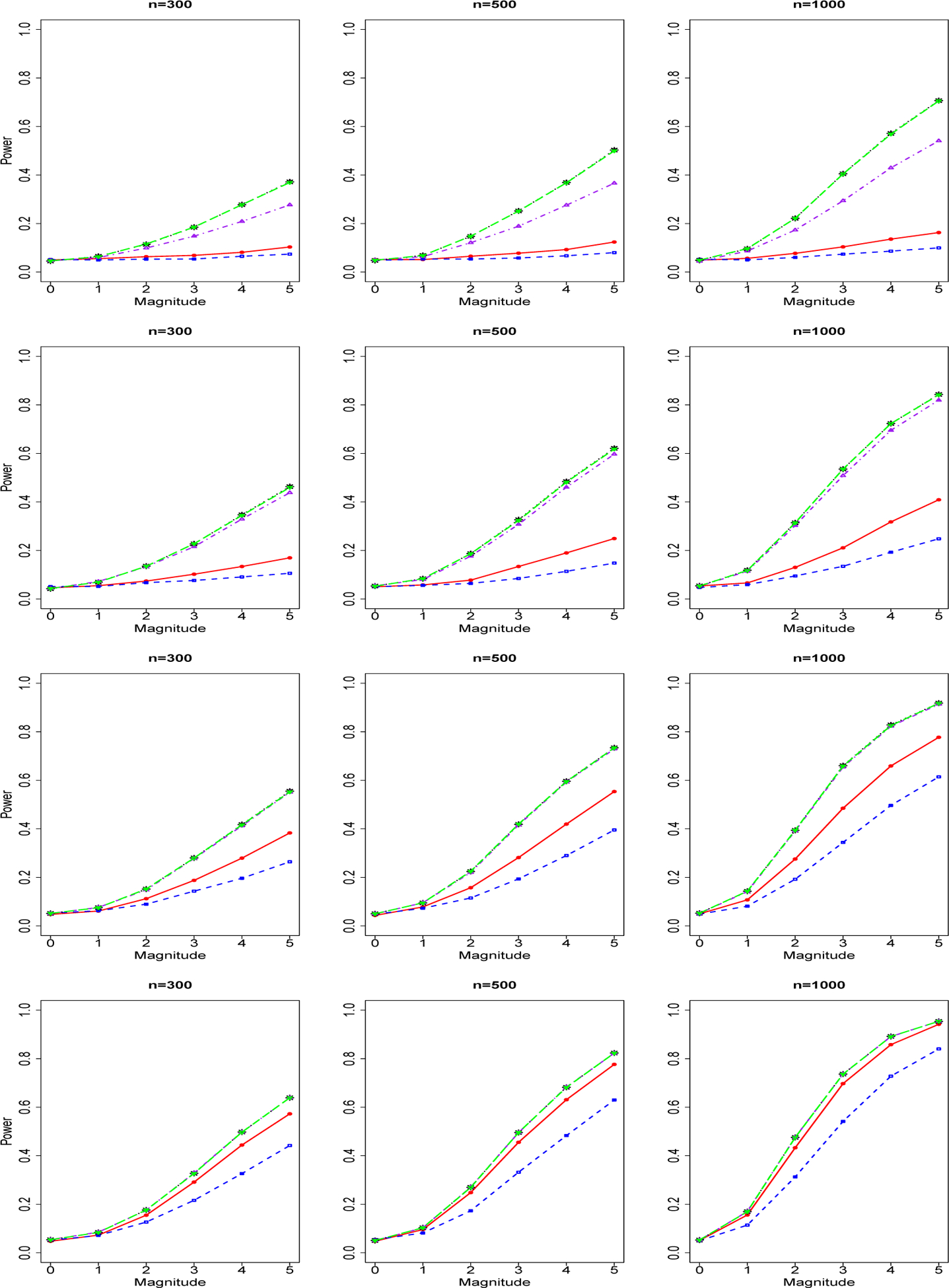
Power Curves of Tests for A Group of Rare Genetic Variants and Binary Phenotype. The four rows of images from up to down are for sequencing depth 1X, 2X, 4X, 10X. The three columns of images from left to right are for sample size n=300,500,1000. Powers of genotype-based burden test (red solid line), genotype-based SKAT test (blue dashed line), NGS data-based variable collapse test 1 (black dotted line), test 2 (purple dotted-dashed line) and test 3 (green long-dashed line).

**Figure 4.**
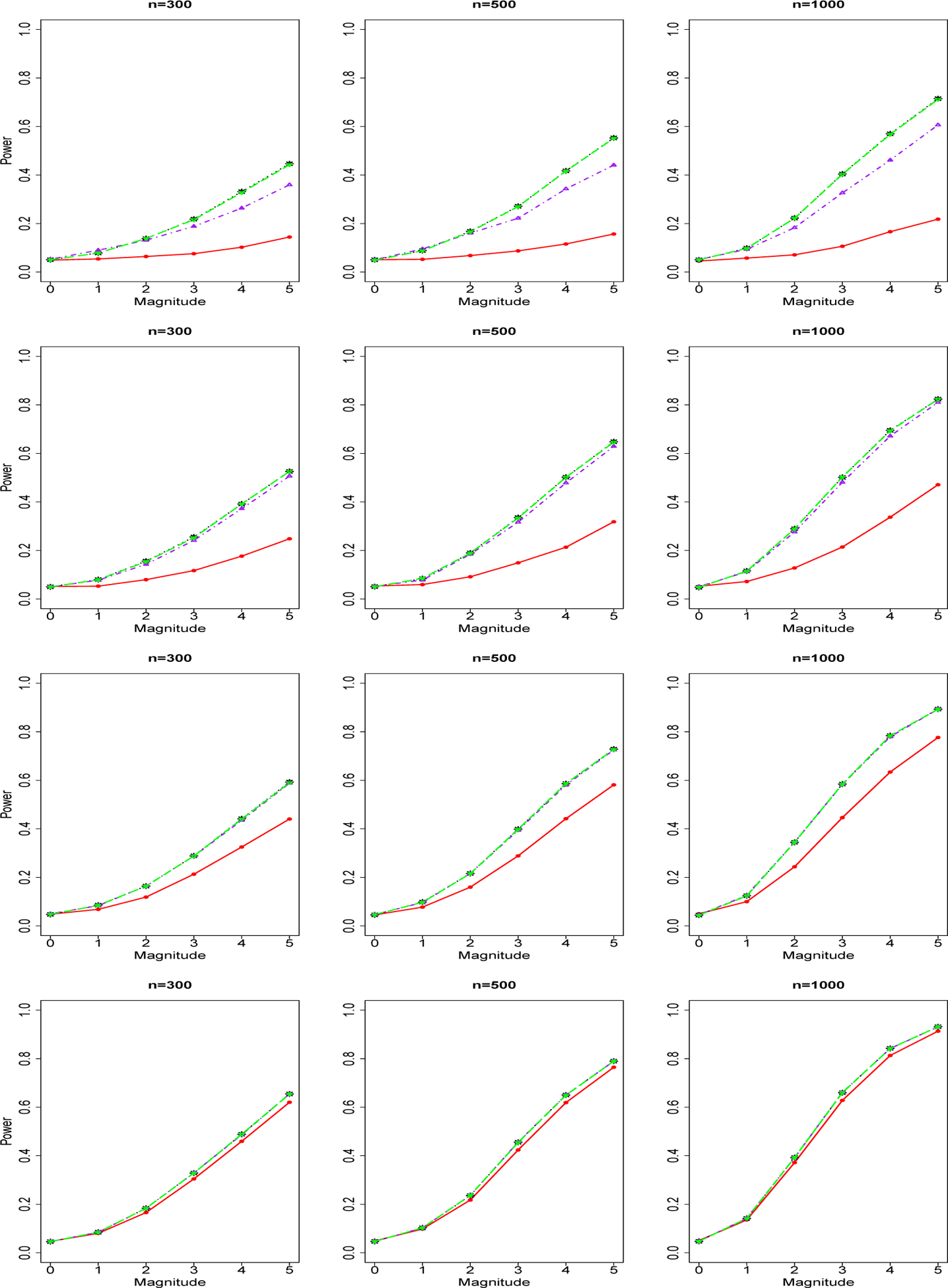
Power Curves of Tests for A Group of Rare Genetic Variants and Count/Integer Phenotype. The four rows of images from up to down are for sequencing depth 1X, 2X, 4X, 10X. The three columns of images from left to right are for sample size n=300,500,1000. Powers of genotype-based burden test (red solid line), NGS data-based variable collapse test 1 (black dotted line), test 2 (purple dotteddashed line) and test 3 (green long-dashed line).

Our simulation studies have considered various scenarios for a range of sample sizes, sequencing depths, number of causal SNPs, and genetic effects for the testing of a group of common variants and a group of rare variants using different testing methods (joint significance test and variable collapse test). We summarize our simulation results as (1) Table 1–4 reporting Type I errors, and (2) Figure 1–4 reporting power analyses. We compare our proposed NGS data-based testing methods with their corresponding genotype-based testing methods. In the following, we present our results on (1) Type I errors, (2) Power analyses, i.e. probability of not committing Type II errors.

### 3.1. Results of Type I Errors

We report the results of Type I errors in all scenarios of sample sizes (300, 500, 1000) and sequencing depths (1X, 2X, 4X, 10X). For different types of genetic variants (common or rare), we apply different testing methods (joint significance test or variable collapse test).

For common genetic variants and continuous phenotypes, we evaluate the Type I errors of our NGS data-based joint significance tests using true allele frequencies (AF) and two ways of estimating allele frequencies as in Skotte *et al.* (2012) and genotype-based F tests [28]. NGS data-based joint significance test 1, 2 and 3 refers *respectively* to NGS-data based joint significance test using (1) true allele frequencies, (2) estimated allele frequencies by a two-step genotype-based method (first estimate genotypes, and then calculate allele frequencies based on the estimated genotypes), and (3) one-step MLE estimator of allele frequencies based on the log-likelihood of sequencing data in Skotte *et al.*(2012)[28]. Both methods of estimating allele frequencies are proposed in Skotte *et al.*(2012)[28]. In general, we expect our NGS data-based testing method will have the best performance when true allele frequencies are used, i.e. NGS data-based Joint Significance Test 1. However, this method (Test 1) is not feasible because in reality, we do not know allele frequencies. Therefore, we need to use estimated allele frequencies which are expected to make performance a little worse but still much better than their corresponding genotype-based methods. We expect the one-step MLE of allele frequency (NGS data-based) to be more accurate than the two-step genotype-based estimator of allele frequency. Thus, we expect NGS data-based joint significance test 3 is better than test 2.

In Table 1, we report simulation results of Type I error for a group of *common genetic variants* with a binary phenotype. The genotype-based Chi-square test conducts genotype calling first, and then conducts a Chi-square test for joint significance of a group of common variants assuming binary phenotype is a logistics model of genotypes. We can see that the genotype-based testing method first estimates genotypes, and then treats the obtained genotypes as true genotypes, and directly conducts the test based on genotypes and phenotypes. In comparison, NGS data-based method treat genotypes as latent variables, and directly models sequencing data and phenotypes. We repeat our simulation study for count/integer phenotype and common genetic variants, and report simulation results of Type I errors in Table 2. According to Table 1 and 2, for both binary response and integer/count response, Type I errors are controlled in most scenarios as we expected.

For rare genetic variants and binary phenotypes, we also evaluated type 1 errors of our sequencing data-based variable collapse tests using true allele frequencies, estimated allele frequencies by the two-step genotype-based estimation method, and estimated allele frequencies by the one-step sequencing data-based estimation method. Like the *joint significance test*, NGS data-based *variable collapse test* 1, 2, 3 refers respectively to our testing methods using true allele frequencies and two allele frequency estimators. In Table 3, we report simulation results of Type I errors for a group of *rare genetic variants* with a binary phenotype. We evaluate the Type I errors of our sequencing data-based variable collapse tests using true allele frequencies and two ways of estimating allele frequencies as previously described, and two genotype-based rare-variant testing methods (burden test and SKAT test). We repeat our simulation study for count/integer phenotype and rare genetic variants and report simulation results of Type I errors in Table 4. Because genotype-based SKAT testing is only available for continuous response and binary response, we compare our methods with genotype-based burden tests. According to Table 3 and 4, for both binary response and integer/count response, Type I errors are controlled in most scenarios as we expected.

### 3.2. Results of Type II Errors and Power Analyses

We evaluate the performance of various methods in the perspective of statistical power. Type 2 error probability is the probability of accepting the null hypothesis when the truth is the alternative hypothesis, which is equal to one minus statistical power, which is the probability of rejecting the null hypothesis when the truth is the alternative hypothesis.

We report in Figure 1 the power curves of tests for a group of *common variants* and a binary phenotype. The four rows from top to bottom are for sequencing depth 1X, 2X, 4X, 10X. The three columns from left to right are for sample size n=300,500,1000. Powers of genotype-based F test (red), NGS data-based joint significance test 1 (blue), test 2 (black) and test 3 (purple) are shown as curves in different colors. We found that in the low sequencing scenario (depth=1X, 2X), there are advantages of NGS-data based methods over genotype-based F test. However, as the sequencing depth increases, the difference between the performance of sequencing-data based methods and the performance of genotype-based F test decreases. In the scenario of sequencing depth 10X, genotype-based methods are nearly the same or only slightly worse than NGS data-based methods. For all the testing methods, we found increasing power as sample size increases or sequencing depth increases. Within the three NGS-data joint significance testing methods, NGS data-based Joint Significance Test 3 (using sequencing data-based MLE of allele frequencies [28]) achieves nearly the same performance as NGS data-based Test 1 (using true allele frequencies). NGS data-based Test 2 (using a two-step genotype-based estimator of allele frequencies) has relatively worse performance compared with Test 1 and Test 3, but Test 2 is still better than their corresponding genotype-based methods. As sequencing depth increases, the difference between the perforamnce of the three NGS data-based testing methods decreases. We repeat our power analysis of common genetic variants for count/integer phenotype. We obtained similar findings for count/integer phenotype and report our results for count/binary phenotype in Figure 2.

We report in Figure 3 the power curve of tests for a group of *rare genetic variants* and binary phenotype. The four rows from top to bottom are for sequencing depth 1X, 2X, 4X, 10X. The three columns from left to right are for sample size n=300,500,1000. Powers of genotype-based burden test (red), SKAT test (blue), NGS data-based variable collapse test 1 (black), test 2 (purple) and test 3 (green). We found that sequencing data-based methods are better than genotype-based methods in the scenarios of low sequencing depths (1X and 2X). As sequencing depth increases, the difference between the performance of genotype-based methods and sequencing data-based methods becomes smaller. For the scenario of sequencing depth 10X, the genotype-based burden test method and sequencing based methods achieve similar performance. Within the three sequencing data-based variable collapse testing methods, Test 3 (estimate allele frequencies using a NGS-based method) achieves nearly the same performance as Test 1 (using true allele frequencies), and Test 2 (estimate allele frequencies using a two-step genotype-based method) is relatively worse compared to Test 1 and 3. As sequencing depth increases, the difference between performances of the three sequencing data-based methods disappears. Within the genotype-based methods, the burden-test achieves better performance than SKAT methods because our scenarios assume all positive genetic effects which are not assumptions of SKAT, which allows both positive effects and negative effects. We repeat our power analysis of rare genetic variants for count/integer phenotype and report in Figure 4 the power curve of tests for a group of *rare genetic variants* and count/integer phenotype. Because SKAT test is not available for count/integer phenotype, we compare our NGS-based methods with genotype-based burden test. We obtained similar findings in count/integer phenotype as we find in binary phenotype.

We also conduct tests under the scenarios consistent with the assumptions of SKAT, i.e. genetic effects in both positive and negative directions, and find that burden tests, no matter whether they are genotype-based tests in the literature or NGS data-based tests proposed by us, fail as we expected because the burden test requires genetic effects to be only in one direction (positive or negative). The failure of the burden test under the scenarios for SKAT was recognized in the literature [23,39]. We leave the work of developing NGS data-based methods corresponding to genotype-based SKAT as our future study since it is beyond the scope of this article. The *main objective* of this article is to use a standard generalized linear model (GLM) framework to develop NGS data-based testing methods corresponding to genotype-based joint significance tests and genotype-based burden test in the literature which can analyze different types of responses especially for binary response and continuous response which are two mostly encountered types in association studies.

## 4. Discussion

In this article, we adopted a standard generalized linear model framework to derive NGS data-based testing methods for *a group of* common genetic variants and a group of rare genetic variants. The proposed methods are extensions of an NGS data-based testing method for *a single genetic variant* proposed by Skotte *et al*. (2012) and Yan *et al*. (2015)[1,28]. With rapid advance in NGS technology and data, there is a big motivation to develop novel methods to deal with NGS data, preferably to have corresponding genotype-based methods in literature for comparison. Since there are established methods for testing a group of common variants and a group of rare variants using genotypes in the literature, our proposed methods fill the literature gap by providing corresponding testing methods using sequencing data instead of the called genotypes.

We adopted a standard generalized linear model framework, and noticed that under the null hypothesis of no genetic effects, the likelihood function and its derivatives can be greatly simplified and easily calculated. Thus, we proposed a score test which only considers the evaluation of score functions and the information matrix at a constrained MLE, and the constrained MLE can be easily obtained. In this way, the chi-square statistic of our joint significance test is derived. Then, we apply the chain rule in calculus to derive our variable collapse test. We have provided full derivation details in the appendix.

There are different models to describe genetic effects of a single marker with genotype g coded as 0, 1 and 2. Four widely-used genetic effect models are modeled so that the effect on linear predictor η in the GLM framework is specified differently: (1) additive model $effect=\beta_{0}+\beta_{a}g$, (2) dominant model: $effect=\beta_{0}+\beta_{d}I(g\geq 1)$, (3) recessive model $effect=\beta_{0}+\beta_{r}I(g=2)$, (4) heterogeneous effect model: $effect=\beta_{0}+\beta_{1}I(g=1)+\beta_{2}I(g=2)$, where I(.) is the indicator function which is equal to 1 when the condition specified in the parenthesis is satisfied, and 0 otherwise [40]. The probability of observing phenotype in our GLM framework is

(13) $$p\left(y_{i}|x_{i},g_{i}\right)=p_{\alpha ,\beta ,\phi }\left(y_{i}|x_{i},g_{i}\right)=exp\left(\frac{y_{i}\eta_{i}-b\left(\eta_{i}\right)}{a(\phi )}+c\left(y_{i},\phi \right)\right),$$

and the linear predictor $\eta_{i}$ is modelled differently: based on dominant model, recessive model and heterogeneous effect model with $d_{g}$ genetic markers. we model linear predictor $\eta_{i}$ respectively as follows:

$$\begin{array}{ll} \eta_{i} & \;=\alpha x_{i}^{T}+\beta_{d}{\left\{I\left(G_{i}\geq 1\right)\right\}}^{T},\\ \eta_{i} & \;=\alpha x_{i}^{T}+\beta_{a}{\left\{I\left(G_{i}=2\right)\right\}}^{T},\\ \eta_{i} & \;=\alpha x_{i}^{T}+\beta_{1}{\left\{I\left(G_{i}=1\right)\right\}}^{T}+\beta_{2}I{\left(G_{i}=2\right)}^{T}, \end{array}$$

where $\beta_{d},\beta_{a},\beta_{1},\beta_{2}$ are row vectors of length $d_{g}$, and $\eta_{i}$ is the linear predictor part. Although our proposed testing methods are described based on the additive model as described in Equation (10), the methods can be adapted to work for the other three genetic effects models by modifying the linear predictor $\eta_{i}$, and its first and second derivatives with respect to model parameters.

The *main objective* of this article is to apply a standard statistical framework to develop novel methods to satisfy the need of group testing of common variants or rare variants based on next-generation data from the society of bioinformatics, biostatistics and modern biology. The proposed methods show advantages over their corresponding genotype-based testing methods in the literature.

Our proposed method is based on a generalized linear model framework dealing with a range of types of phenotypes including binary responses and continuous responses which are two mostly encountered types in association studies. Our method extended our previous linear model framework [2] with the analytical capacity for more complex phenotypes in addition to continuous phenotypes.

Sequencing depth plays an important role in performance comparison of NGS data-based methods versus their corresponding genotype-based methods. When sequencing is deep, genotype can be precisely called, and there are no big differences between the performances of sequencing data-based methods and their corresponding genotype-based methods. However, when sequencing depth is not big (1X, 2X), genotypes can not be precisely called so that sequencing-data based methods can achieve better performance compared with their corresponding genotype-based methods [1,28]. Given the same budget constraint, a low sequencing depth with more individuals sequenced is preferred to a deep sequencing depth with few individuals sequenced [1, 28]. Sequencing depths can also be different for different individuals. Future studies can be on NGS data-based association testing for individuals with heterogeneous sequencing depths.

## 5. Conclusion

We extend our previously proposed NGS data-based testing methods (joint significance test for a group of common variants and variable collapse test for a group of rare variants) from a linear model (LM) framework to a generalized linear model (GLM) framework so that it can handle a range of types of responses (binary phenotypes; count/integer phenotypes) in addition to continuous phenotypes. Our proposed methods fill the literature gap, and can achieve better performance compared with their corresponding genotype-based testing methods in the literature.

## Data Availability Statement:

All data used in the study is publicly available.

## Figures and Tables

**Table 1.** Type I Errors of Testing Methods for a Group of Common Genetic Variants and Binary Phenotype. Genotype-based $\chi^{2}$ test refers to Genotype-based Chi-square test. NGS JS Test 1, 2, 3 refer to NGS data-based joint significant test with use of (1) true allele frequency (AF), (2) estimated AF by genotype-based method, and (3) estimated AF by NGS data-based method.

Sample Size	Depth	Genotype-based $\chi^{2}$ test	NGS JS Test 1	NGS JS Test 2	NGS JS Test 3
300	1	0.050	0.050	0.050	0.049
500	1	0.047	0.050	0.053	0.050
1000	1	0.047	0.046	0.048	0.046
300	2	0.050	0.050	0.053	0.049
500	2	0.051	0.051	0.052	0.050
1000	2	0.050	0.050	0.049	0.049
300	4	0.050	0.048	0.049	0.049
500	4	0.054	0.050	0.050	0.050
1000	4	0.044	0.048	0.048	0.048
300	10	0.054	0.046	0.047	0.047
500	10	0.052	0.047	0.047	0.047
1000	10	0.047	0.051	0.052	0.052

**Table 2.** Type I Errors of Testing Methods for a Group of Common Genetic Variants and Count/Integer Phenotype. Genotype-based $\chi^{2}$ test refers to Genotype-based Chi-square test. NGS JS Test 1, 2, 3 refer to NGS data-based joint significant test with use of (1) true allele frequency (AF), (2) estimated AF by genotype-based method, and (3) estimated AF by NGS data-based method.

Sample Size	Depth	Genotype-based $\chi^{2}$ test	NGS JS Test 1	NGS JS Test 2	NGS JS Test 3
300	1	0.047	0.052	0.052	0.052
500	1	0.047	0.051	0.052	0.051
1000	1	0.054	0.051	0.051	0.052
300	2	0.053	0.052	0.050	0.052
500	2	0.053	0.050	0.050	0.050
1000	2	0.052	0.045	0.043	0.044
300	3	0.046	0.052	0.052	0.052
500	3	0.052	0.049	0.047	0.048
1000	3	0.050	0.051	0.050	0.051
300	4	0.049	0.049	0.050	0.049
500	4	0.052	0.048	0.047	0.047
1000	4	0.050	0.046	0.046	0.046

**Table 3.** Type I Errors of Testing Methods for a Group of Rare Genetic Variants and Binary Phenotype. Burden and SKAT refer to genotype-based burden test and SKAT test. NGS VC Test 1, 2, 3 refer to NGS data-based variable collapse test with use of (1) true allele frequency (AF), (2) estimated AF by genotype-based method, and (3) estimated AF by NGS data-based method.

Sample Size	Depth	Burden	SKAT	NGS VC Test 1	NGS VC Test 2	NGS VC Test 3
300	1	0.050	0.052	0.045	0.046	0.046
500	1	0.052	0.048	0.048	0.050	0.049
1000	1	0.049	0.053	0.049	0.045	0.049
300	2	0.046	0.051	0.042	0.043	0.043
500	2	0.050	0.050	0.053	0.054	0.053
1000	2	0.054	0.047	0.054	0.053	0.053
300	3	0.048	0.052	0.051	0.051	0.052
500	3	0.043	0.048	0.050	0.049	0.050
1000	3	0.050	0.049	0.052	0.052	0.052
300	4	0.048	0.052	0.054	0.054	0.054
500	4	0.047	0.054	0.051	0.050	0.051
1000	4	0.051	0.050	0.052	0.052	0.053

**Table 4.** Type I Errors of Testing Methods for a Group of Rare Genetic Variants and Count/Integer Phenotype. Burden refers to genotype-based burden test. NGS VC Test 1, 2, 3 refer to NGS data-based variable collapse test with use of (1) true allele frequency (AF), (2) estimated AF by genotype-based method, and (3) estimated AF by NGS data-based method.

Sample Size	Depth	Burden	NGS VC Test 1	NGS VC Test 2	NGS VC Test 3
300	1	0.049	0.051	0.053	0.050
500	1	0.051	0.051	0.051	0.049
1000	1	0.045	0.050	0.052	0.050
300	2	0.052	0.050	0.050	0.050
500	2	0.054	0.051	0.052	0.051
1000	2	0.052	0.048	0.051	0.048
300	3	0.048	0.047	0.050	0.048
500	3	0.045	0.045	0.047	0.046
1000	3	0.050	0.044	0.045	0.044
300	4	0.047	0.046	0.046	0.046
500	4	0.049	0.047	0.046	0.047
1000	4	0.050	0.047	0.048	0.047

## Appendix A Detailed Derivation for Simplification of Log-Likelihood Under Null Hypothesis $H_{0}:\beta =0$

When parameters are (α,β,ϕ),

$$\begin{array}{ll} l_{y,D}(\alpha ,0,\phi ) & \;=\sum_{i=1}^{N}\;\;\left(log\left(\sum_{g\in 𝒢}\;\;f_{\theta }\left(y_{i}|x_{i},g\right)h\left(g,D_{i}\right)\right)\right)=\sum_{i=1}^{N}\;\;\left(log\left(f_{\theta }\left(y_{i}|x_{i}\right)\sum_{g\in 𝒢}\;\;h\left(g,D_{i}\right)\right)\right)\\ & \left.=\sum_{i=1}^{N}\;\;\left(log\left(f_{\theta }\left(y_{i}|x_{i}\right)\right)+log\left(\sum_{g\in 𝒢}\;\;h\left(g,D_{i}\right)\right)\right)=\sum_{i=1}^{N}\;\;log\left(f_{\theta }\left(y_{i}|x_{i}\right)\right)+\sum_{i=1}^{N}\;\;log\left(\sum_{g\in 𝒢}\;\;h\left(g,D_{i}\right)\right)\right)\\ & \;=\sum_{i=1}^{N}\;\;log\left(f_{\theta }\left(y_{i}|x_{i}\right)\right)+\text{ constant in terms of parameters }\\ & \;=\sum_{i=1}^{N}\;\;\left\{\frac{y_{i}\eta_{i}-b\left(\eta_{i}\right)}{a(\phi )}+c\left(y_{i},\phi \right)\right\}+\text{ constant in terms of parameters }\\ & \;=\sum_{i=1}^{N}\;\;\left\{\frac{y_{i}\left(\alpha x_{i}^{T}\right)-b\left(\alpha x_{i}^{T}\right)}{a(\phi )}+c\left(y_{i},\phi \right)\right\}+\text{ constant in terms of parameters. } \end{array}$$

When parameters are (α,β), i.e. ϕ is dropped out,

$$\begin{array}{ll} l_{y,D}(\alpha ,0) & \;=\sum_{i=1}^{N}\;\;\left(log\left(\sum_{g\in 𝒢}\;\;f_{\theta }\left(y_{i}|x_{i},g\right)h\left(g,D_{i}\right)\right)\right)=\sum_{i=1}^{N}\;\;\left(log\left(f_{\theta }\left(y_{i}|x_{i}\right)\sum_{g\in 𝒢}\;\;h\left(g,D_{i}\right)\right)\right)\\ & \left.=\sum_{i=1}^{N}\;\;\left(log\left(f_{\theta }\left(y_{i}|x_{i}\right)\right)+log\left(\sum_{g\in 𝒢}\;\;h\left(g,D_{i}\right)\right)\right)=\sum_{i=1}^{N}\;\;log\left(f_{\theta }\left(y_{i}|x_{i}\right)\right)+\sum_{i=1}^{N}\;\;log\left(\sum_{g\in 𝒢}\;\;h\left(g,D_{i}\right)\right)\right)\\ & \;=\sum_{i=1}^{N}\;\;log\left(f_{\theta }\left(y_{i}|x_{i}\right)\right)+\text{ constant in terms of parameters }\\ & \;=\sum_{i=1}^{N}\;\;\left\{\frac{y_{i}\eta_{i}-b\left(\eta_{i}\right)}{a}+c\left(y_{i}\right)\right\}+\text{ constant in terms of parameters }\\ & \;=\sum_{i=1}^{N}\;\;\left\{\frac{y_{i}\left(\alpha x_{i}^{T}\right)-b\left(\alpha x_{i}^{T}\right)}{a}+c\left(y_{i}\right)\right\}+\text{ constant in terms of parameters. } \end{array}$$

## Appendix B Analytical Formula of Score Function when Parameters are (α,β,ϕ)

The score function is

$$s_{y,D}(\alpha ,\beta ,\phi )=\left[\begin{array}{l} \frac{\partial l_{y,D}(\alpha ,\beta ,\phi )}{\partial \alpha^{T}}\\ \frac{\partial l_{y,D}(\alpha ,\beta ,\phi )}{\partial \beta^{T}}\\ \frac{\partial l_{y,D}(\alpha ,\beta ,\phi )}{\partial \phi } \end{array}\right]$$

We derived each element in the above vector as the following.

$$\begin{array}{ll} & \frac{\partial l_{y,D}(\alpha ,\beta ,\phi )}{\partial \alpha^{T}}=\frac{\partial \sum_{i=1}^{N}\;\;ln\left(p_{\theta }\left(y_{i},D_{i}|x_{i}\right)\right)}{\partial \alpha^{T}}=\sum_{i=1}^{N}\;\;\frac{\partial ln\left(p_{\theta }\left(y_{i},D_{i}|x_{i}\right)\right)}{\partial \alpha^{T}}\\ & \;=\sum_{i=1}^{N}\;\;\left[{\left\{p_{\theta }\left(y_{i},D_{i}|x_{i}\right)\right\}}^{-1}\frac{\partial p_{\theta }\left(y_{i},D_{i}|x_{i}\right)}{\partial \alpha^{T}}\right]\\ & \;=\sum_{i=1}^{N}\;\;\left[{\left\{p_{\theta }\left(y_{i},D_{i}|x_{i}\right)\right\}}^{-1}\frac{\partial \sum_{g\in 𝒢}\;\;f_{\theta }\left(y_{i}|x_{i},g\right)h\left(g,D_{i}\right)}{\partial \alpha^{T}}\right]\\ & \;=\sum_{i=1}^{N}\;\;\left[{\left\{p_{\theta }\left(y_{i},D_{i}|x_{i}\right)\right\}}^{-1}\sum_{g\in 𝒢}\;\;\frac{\partial \left\{exp\left(\frac{y_{i}\eta_{i}-b\left(\eta_{i}\right)}{a(\phi )}+c\left(y_{i},\phi \right)\right)\right\}}{\partial \alpha^{T}}h\left(g,D_{i}\right)\right]\\ & \;=\sum_{i=1}^{N}\;\;\left[{\left\{p_{\theta }\left(y_{i},D_{i}|x_{i}\right)\right\}}^{-1}\sum_{g\in 𝒢}\;\;exp\left\{\frac{y_{i}\eta_{i}-b\left(\eta_{i}\right)}{a(\phi )}+c\left(y_{i},\phi \right)\right\}\frac{y_{i}-b^{'}\left(\eta_{i}\right)}{a(\phi )}x_{i}^{T}h\left(g,D_{i}\right)\right]\\ & \;=\sum_{i=1}^{N}\;\;\left[{\left\{p_{\theta }\left(y_{i},D_{i}|x_{i}\right)\right\}}^{-1}\sum_{g\in 𝒢}\;\;f_{\theta }\left(y_{i}|x_{i},g\right)\frac{y_{i}-b^{'}\left(\eta_{i}\right)}{a(\phi )}x_{i}^{T}h\left(g,D_{i}\right)\right].\\ & \;\\ & \;\\ & \;\\ & \; \end{array}$$

$$\frac{\partial l_{y,D}(\alpha ,\beta ,\phi )}{\partial \beta^{T}}=\frac{\partial \sum_{i=1}^{N}\;\;log\left(p_{\theta }\left(y_{i},D_{i}|x_{i}\right)\right)}{\partial \beta^{T}}=\sum_{i=1}^{N}\;\;\frac{\partial log\left(p_{\theta }\left(y_{i},D_{i}|x_{i}\right)\right)}{\partial \beta^{T}}=\sum_{i=1}^{N}\;\;\left[{\left\{p_{\theta }\left(y_{i},D_{i}|x_{i}\right)\right\}}^{-1}\frac{\partial p_{\theta }\left(y_{i},D_{i}|x_{i}\right)}{\partial \beta^{T}}\right]=\sum_{i=1}^{N}\;\;\left[{\left\{p_{\theta }\left(y_{i},D_{i}|x_{i}\right)\right\}}^{-1}\frac{\partial \sum_{g\in 𝒢}\;\;f_{\theta }\left(y_{i}|x_{i},g\right)h\left(g,D_{i}\right)}{\partial \beta^{T}}\right]\;=\sum_{i=1}^{N}\;\;\left[{\left\{p_{\theta }\left(y_{i},D_{i}|x_{i}\right)\right\}}^{-1}\sum_{g\in 𝒢}\;\;\frac{\partial \left\{exp\left(\frac{y_{i}\eta_{i}-b\left(\eta_{i}\right)}{a(\phi )}+c\left(y_{i},\phi \right)\right)\right\}}{\partial \beta^{T}}h\left(g,D_{i}\right)\right]=\sum_{i=1}^{N}\;\;{\left\{p_{\theta }\left(y_{i},D_{i}|x_{i}\right)\right\}}^{-1}\sum_{g\in 𝒢}\;\;exp\left\{\frac{y_{i}\eta_{i}-b\left(\eta_{i}\right)}{a(\phi )}+c\left(y_{i},\phi \right)\right\}\frac{y_{i}-b^{'}\left(\eta_{i}\right)}{a(\phi )}g^{T}h\left(g,D_{i}\right)=\sum_{i=1}^{N}\;\;{\left\{p_{\theta }\left(y_{i},D_{i}|x_{i}\right)\right\}}^{-1}\sum_{g\in 𝒢}\;\;f_{\theta }\left(y_{i}|x_{i},g\right)\frac{y_{i}-b^{'}\left(\eta_{i}\right)}{a(\phi )}g^{T}h\left(g,D_{i}\right).$$

$$\begin{array}{ll} \frac{\partial l_{y,D}(\alpha ,\beta ,\phi )}{\partial \phi } & \;=\frac{\partial \sum_{i=1}^{N}\;\;log\left(p_{\theta }\left(y_{i},D_{i}|x_{i}\right)\right)}{\partial \phi }=\sum_{i=1}^{N}\;\;\frac{\partial log\left(p_{\theta }\left(y_{i},D_{i}|x_{i}\right)\right)}{\partial \phi }\\ & \;=\sum_{i=1}^{N}\;\;\left[{\left\{p_{\theta }\left(y_{i},D_{i}|x_{i}\right)\right\}}^{-1}\frac{\partial p_{\theta }\left(y_{i},D_{i}|x_{i}\right)}{\partial \phi }\right]\\ & \;=\sum_{i=1}^{N}\;\;\left[{\left\{p_{\theta }\left(y_{i},D_{i}|x_{i}\right)\right\}}^{-1}\frac{\partial \sum_{g\in 𝒢}\;\;f_{\theta }\left(y_{i}|x_{i},g\right)h\left(g,D_{i}\right)}{\partial \phi }\right]\\ & \;=\left[\sum_{i=1}^{N}\;\;{\left\{p_{\theta }\left(y_{i},D_{i}|x_{i}\right)\right\}}^{-1}\sum_{g\in 𝒢}\;\;\frac{\partial \left\{exp\left(\frac{y_{i}\eta_{i}-b\left(\eta_{i}\right)}{a(\phi )}+c\left(y_{i},\phi \right)\right)\right\}}{\partial \phi }h\left(g,D_{i}\right)\right]\\ & \;=\sum_{i=1}^{N}\;\;\left[{\left\{p_{\theta }\left(y_{i},D_{i}|x_{i}\right)\right\}}^{-1}\sum_{g\in 𝒢}\;\;f_{\theta }\left(y_{i}|x_{i},g\right)\left\{-\frac{y_{i}\eta_{i}-b\left(\eta_{i}\right)}{[a(\phi ){]}^{2}}a^{'}(\phi )+\frac{\partial c\left(y_{i},\phi \right)}{\partial \phi }\right\}h\left(g,D_{i}\right)\right]. \end{array}$$

So write compactly, we have

$$s_{y,D}(\alpha ,\beta ,\phi )=\sum_{i=1}^{N}\;\left[{\left\{p_{\theta }\left(y_{i},D_{i}|x_{i}\right)\right\}}^{-1}\sum_{g\in 𝒢}\;\;f_{\theta }\left(y_{i}|x_{i},g\right)\left[\begin{array}{c} \frac{y_{i}-b^{'}\left(\eta_{i}\right)}{a(\phi )}x_{i}^{T}\\ \frac{y_{i}-b^{'}\left(\eta_{i}\right)}{a(\phi )}g^{T}\\ -\frac{y_{i}\eta_{i}-b\left(\eta_{i}\right)}{[a(\phi ){]}^{2}}a^{'}(\phi )+\frac{\partial c\left(y_{i},\phi \right)}{\partial \phi } \end{array}\right]h\left(g,D_{i}\right)\right],$$

where $\eta_{i}=\eta \left(x_{i},g\right)=\alpha x_{i}^{T}+\beta g^{T}$.

## Appendix C Analytical Formula of Observed Information Matrix when Parameters are (α,β,ϕ)

The observed information is

$$o_{y,D}(\alpha ,\beta ,\phi )=-\left[\begin{array}{lll} \frac{\partial^{2}l_{y,D}(\alpha ,\beta ,\phi )}{\partial \alpha^{T}\partial \alpha } & \frac{\partial^{2}l_{y,D}(\alpha ,\beta ,\phi )}{\partial \alpha^{T}\partial \beta } & \frac{\partial^{2}l_{y,D}(\alpha ,\beta ,\phi )}{\partial \alpha^{T}\partial \phi }\\ \frac{\partial^{2}l_{y,D}(\alpha ,\beta ,\phi )}{\partial \beta^{T}\partial \alpha } & \frac{\partial^{2}l_{y,D}(\alpha ,\beta ,\phi )}{\partial \beta^{T}\partial \beta } & \frac{\partial^{2}l_{y,D}(\alpha ,\beta ,\phi )}{\partial \beta^{T}\partial \phi }\\ \frac{\partial^{2}l_{y,D}(\alpha ,\beta ,\phi )}{\partial \phi \partial \alpha } & \frac{\partial^{2}l_{y,D}(\alpha ,\beta ,\phi )}{\partial \phi \partial \beta } & \frac{\partial^{2}l_{y,D}(\alpha ,\beta ,\phi )}{\partial \phi^{2}} \end{array}\right]$$

We derive each element in the matrix as the following.

We start from the score function, which is derived as

$$s_{y,D}\left(\alpha ,\beta ,\phi \right)=\sum_{i=1}^{N}\;\left[{\left\{p_{\theta }\left(y_{i},D_{i}|x_{i}\right)\right\}}^{-1}\sum_{g\in 𝒢}\;\;f_{\theta }\left(y_{i}|x_{i},g\right)\left[\begin{array}{c} \frac{y_{i}-b^{'}\left(\eta_{i}\right)}{a\left(\phi \right)}x_{i}^{T}\\ \frac{y_{i}-b^{'}\left(\eta_{i}\right)}{a\left(\phi \right)}g^{T}\\ -\frac{y_{i}\eta_{i}-b\left(\eta_{i}\right)}{[a(\phi ){]}^{2}}a^{'}\left(\phi \right)+\frac{\partial c\left(y_{i},\phi \right)}{\partial \phi } \end{array}\right]h\left(g,D_{i}\right)\right],$$

where $\eta_{i}=\eta \left(x_{i},g\right)=\alpha x_{i}^{T}+\beta g^{T}$. Take the derivative of the score function.

$$\frac{\partial^{2}l_{y,D}(\alpha ,\beta ,\phi )}{\partial \alpha^{T}\partial \alpha }=\frac{\partial \frac{\partial l_{y,D}(\alpha ,\beta ,\phi )}{\partial \alpha^{T}}}{\partial \alpha }\;\;=\frac{\partial \left[\sum_{i=1}^{N}\;\;{\left\{p_{\theta }\left(y_{i},D_{i}|x_{i}\right)\right\}}^{-1}\sum_{g\in 𝒢}\;\;f_{\theta }\left(y_{i}|x_{i},g\right)\frac{y_{i}-b^{'}\left(\eta_{i}\right)}{a(\phi )}x_{i}^{T}h\left(g,D_{i}\right)\right]}{\partial \alpha }\;\;=\sum_{i=1}^{N}\;\;\frac{\partial \left[{\left\{p_{\theta }\left(y_{i},D_{i}|x_{i}\right)\right\}}^{-1}\sum_{g\in 𝒢}\;\;f_{\theta }\left(y_{i}|x_{i},g\right)\frac{y_{i}-b^{'}\left(\eta_{i}\right)}{a(\phi )}x_{i}^{T}h\left(g,D_{i}\right)\right]}{\partial \alpha }\;\;=\sum_{i=1}^{N}\;\;\left[\sum_{g\in 𝒢}\;\;f_{\theta }\left(y_{i}|x_{i},g\right)\frac{y_{i}-b^{'}\left(\eta_{i}\right)}{a(\phi )}x_{i}^{T}h\left(g,D_{i}\right)\right]\frac{\partial {\left\{p_{\theta }\left(y_{i},D_{i}|x_{i}\right)\right\}}^{-1}}{\partial \alpha }+{\left\{p_{\theta }\left(y_{i},D_{i}|x_{i}\right)\right\}}^{-1}\frac{\partial \sum_{g\in 𝒢}\;\;f_{\theta }\left(y_{i}|x_{i},g\right)\frac{y_{i}-b^{'}\left(\eta_{i}\right)}{a(\phi )}x_{i}^{T}h\left(g,D_{i}\right)}{\partial \alpha }\;\;=\sum_{i=1}^{N}\;\;\left[\sum_{g\in 𝒢}\;\;f_{\theta }\left(y_{i}|x_{i},g\right)\frac{y_{i}-b^{'}\left(\eta_{i}\right)}{a(\phi )}x_{i}^{T}h\left(g,D_{i}\right)\right]\left[\left(-{\left\{p_{\theta }\left(y_{i},D_{i}|x_{i}\right)\right\}}^{-2}\right)\sum_{g\in 𝒢}\;\;f_{\theta }\left(y_{i}|x_{i},g\right)\frac{y_{i}-b^{'}\left(\eta_{i}\right)}{a(\phi )}x_{i}h\left(g,D_{i}\right)\right]\;\;+{\left\{p_{\theta }\left(y_{i},D_{i}|x_{i}\right)\right\}}^{-1}\sum_{g\in 𝒢}\;\;\frac{\partial f_{\theta }\left(y_{i}|x_{i},g\right)\frac{y_{i}-b^{'}\left(\eta_{i}\right)}{a(\phi )}x_{i}^{T}}{\partial \alpha }h\left(g,D_{i}\right)\;\;=\sum_{i=1}^{N}\;\;\left(-{\left\{p_{\theta }\left(y_{i},D_{i}|x_{i}\right)\right\}}^{-2}\right)\left[\sum_{g\in 𝒢}\;\;f_{\theta }\left(y_{i}|x_{i},g\right)\frac{y_{i}-b^{'}\left(\eta_{i}\right)}{a(\phi )}x_{i}^{T}h\left(g,D_{i}\right)\right]\left[\sum_{g\in 𝒢}\;\;f_{\theta }\left(y_{i}|x_{i},g\right)\frac{y_{i}-b^{'}\left(\eta_{i}\right)}{a(\phi )}x_{i}h\left(g,D_{i}\right)\right]\;\;+{\left\{p_{\theta }\left(y_{i},D_{i}|x_{i}\right)\right\}}^{-1}\sum_{g\in 𝒢}\;\;f_{\theta }\left(y_{i}|x_{i},g\right)\left[\left(\frac{y_{i}-b^{'}\left(\eta_{i}\right)}{a(\phi )}x_{i}^{T}\right)\frac{\partial \left[\frac{y_{i}\eta_{i}-b\left(\eta_{i}\right)}{a(\phi )}+c\left(y_{i},\phi \right)\right]}{\partial \alpha }+\frac{\partial \left[\frac{y_{i}-b^{'}\left(\eta_{i}\right)}{a(\phi )}x_{i}^{T}\right]}{\partial \alpha }\right]h\left(g,D_{i}\right)\;\;=\sum_{i=1}^{N}\;\;\left(-{\left\{p_{\theta }\left(y_{i},D_{i}|x_{i}\right)\right\}}^{-2}\right)\left[\sum_{g\in 𝒢}\;\;f_{\theta }\left(y_{i}|x_{i},g\right)\frac{y_{i}-b^{'}\left(\eta_{i}\right)}{a(\phi )}x_{i}^{T}h\left(g,D_{i}\right)\right]\left[\sum_{g\in 𝒢}\;\;f_{\theta }\left(y_{i}|x_{i},g\right)\frac{y_{i}-b^{'}\left(\eta_{i}\right)}{a(\phi )}x_{i}h\left(g,D_{i}\right)\right]\;\;+{\left\{p_{\theta }\left(y_{i},D_{i}|x_{i}\right)\right\}}^{-1}\sum_{g\in 𝒢}\;\;f_{\theta }\left(y_{i}|x_{i},g\right)\left[\left(\frac{y_{i}-b^{'}\left(\eta_{i}\right)}{a(\phi )}x_{i}^{T}\right)\left(\frac{y_{i}-b^{'}\left(\eta_{i}\right)}{a(\phi )}x_{i}\right)-\frac{b^{''}\left(\eta_{i}\right)}{a(\phi )}x_{i}^{T}x_{i}\right]h\left(g,D_{i}\right)\;\;=\sum_{i=1}^{N}\;\;\left(-{\left\{p_{\theta }\left(y_{i},D_{i}|x_{i}\right)\right\}}^{-2}\right)\left[\sum_{g\in 𝒢}\;\;f_{\theta }\left(y_{i}|x_{i},g\right)\frac{y_{i}-b^{'}\left(\eta_{i}\right)}{a(\phi )}x_{i}^{T}h\left(g,D_{i}\right)\right]\left[\sum_{g\in 𝒢}\;\;f_{\theta }\left(y_{i}|x_{i},g\right)\frac{y_{i}-b^{'}\left(\eta_{i}\right)}{a(\phi )}x_{i}h\left(g,D_{i}\right)\right]\;\;+{\left\{p_{\theta }\left(y_{i},D_{i}|x_{i}\right)\right\}}^{-1}\sum_{g\in 𝒢}\;\;f_{\theta }\left(y_{i}|x_{i},g\right)\left[\frac{{\left(y_{i}-b^{'}\left(\eta_{i}\right)\right)}^{2}}{[a(\phi ){]}^{2}}-\frac{b^{''}\left(\eta_{i}\right)}{a(\phi )}\right]x_{i}^{T}x_{i}h\left(g,D_{i}\right)$$

$$\frac{\partial^{2}l_{y,D}(\alpha ,\beta ,\phi )}{\partial \alpha^{T}\partial \beta }=\frac{\partial \frac{\partial l_{y,D}(\alpha ,\beta ,\phi )}{\partial \alpha^{T}}}{\partial \beta }\;\;=\frac{\partial \left[\sum_{i=1}^{N}\;\;{\left\{p_{\theta }\left(y_{i},D_{i}|x_{i}\right)\right\}}^{-1}\sum_{g\in 𝒢}\;\;f_{\theta }\left(y_{i}|x_{i},g\right)\frac{y_{i}-b^{'}\left(\eta_{i}\right)}{a(\phi )}x_{i}^{T}h\left(g,D_{i}\right)\right]}{\partial \beta }\;\;=\sum_{i=1}^{N}\;\;\frac{\partial \left[{\left\{p_{\theta }\left(y_{i},D_{i}|x_{i}\right)\right\}}^{-1}\sum_{g\in 𝒢}\;\;f_{\theta }\left(y_{i}|x_{i},g\right)\frac{y_{i}-b^{'}\left(\eta_{i}\right)}{a(\phi )}x_{i}^{T}h\left(g,D_{i}\right)\right]}{\partial \beta }\;\;=\sum_{i=1}^{N}\;\;\left[\sum_{g\in 𝒢}\;\;f_{\theta }\left(y_{i}|x_{i},g\right)\frac{y_{i}-b^{'}\left(\eta_{i}\right)}{a(\phi )}x_{i}^{T}h\left(g,D_{i}\right)\right]\frac{\partial {\left\{p_{\theta }\left(y_{i},D_{i}|x_{i}\right)\right\}}^{-1}}{\partial \beta }+{\left\{p_{\theta }\left(y_{i},D_{i}|x_{i}\right)\right\}}^{-1}\frac{\partial \left[\sum_{g\in 𝒢}\;\;f_{\theta }\left(y_{i}|x_{i},g\right)\frac{y_{i}-b^{'}\left(\eta_{i}\right)}{a(\phi )}x_{i}^{T}h\left(g,D_{i}\right)\right.}{\partial \beta }\;\;=\sum_{i=1}^{N}\;\;\left[\sum_{g\in 𝒢}\;\;f_{\theta }\left(y_{i}|x_{i},g\right)\frac{y_{i}-b^{'}\left(\eta_{i}\right)}{a(\phi )}x_{i}^{T}h\left(g,D_{i}\right)\right]\left[\left(-{\left\{p_{\theta }\left(y_{i},D_{i}|x_{i}\right)\right\}}^{-2}\right)\sum_{g\in 𝒢}\;\;f_{\theta }\left(y_{i}|x_{i},g\right)\frac{y_{i}-b^{'}\left(\eta_{i}\right)}{a(\phi )}gh\left(g,D_{i}\right)\right]\;\;+{\left\{p_{\theta }\left(y_{i},D_{i}|x_{i}\right)\right\}}^{-1}\sum_{g\in 𝒢}\;\;\frac{\partial \left[f_{\theta }\left(y_{i}|x_{i},g\right)\frac{y_{i}-b^{'}\left(\eta_{i}\right)}{a(\phi )}x_{i}^{T}\right]}{\partial \beta }h\left(g,D_{i}\right)\;\;=\sum_{i=1}^{N}\;\;\left(-{\left\{p_{\theta }\left(y_{i},D_{i}|x_{i}\right)\right\}}^{-2}\right)\left[\sum_{g\in 𝒢}\;\;f_{\theta }\left(y_{i}|x_{i},g\right)\frac{y_{i}-b^{'}\left(\eta_{i}\right)}{a(\phi )}x_{i}^{T}h\left(g,D_{i}\right)\right]\left[\sum_{g\in 𝒢}\;\;f_{\theta }\left(y_{i}|x_{i},g\right)\frac{y_{i}-b^{'}\left(\eta_{i}\right)}{a(\phi )}gh\left(g,D_{i}\right)\right]\;\;+{\left\{p_{\theta }\left(y_{i},D_{i}|x_{i}\right)\right\}}^{-1}\sum_{g\in 𝒢}\;\;f_{\theta }\left(y_{i}|x_{i},g\right)\left[\left(\frac{y_{i}-b^{'}\left(\eta_{i}\right)}{a(\phi )}x_{i}^{T}\right)\frac{\partial \left[\frac{y_{i}\eta_{i}-b\left(\eta_{i}\right)}{a(\phi )}+c\left(y_{i},\phi \right)\right]}{\partial \beta }+\frac{\partial \left[\frac{y_{i}-b^{'}\left(\eta_{i}\right)}{a(\phi )}x_{i}^{T}\right]}{\partial \beta }\right]h\left(g,D_{i}\right)\;\;=\sum_{i=1}^{N}\;\;\left(-{\left\{p_{\theta }\left(y_{i},D_{i}|x_{i}\right)\right\}}^{-2}\right)\left[\sum_{g\in 𝒢}\;\;f_{\theta }\left(y_{i}|x_{i},g\right)\frac{y_{i}-b^{'}\left(\eta_{i}\right)}{a(\phi )}x_{i}^{T}h\left(g,D_{i}\right)\right]\left[\sum_{g\in 𝒢}\;\;f_{\theta }\left(y_{i}|x_{i},g\right)\frac{y_{i}-b^{'}\left(\eta_{i}\right)}{a(\phi )}gh\left(g,D_{i}\right)\right]\;\;+{\left\{p_{\theta }\left(y_{i},D_{i}|x_{i}\right)\right\}}^{-1}\sum_{g\in 𝒢}\;\;f_{\theta }\left(y_{i}|x_{i},g\right)\left[\left(\frac{y_{i}-b^{'}\left(\eta_{i}\right)}{a(\phi )}x_{i}^{T}\right)\left(\frac{y_{i}-b^{'}\left(\eta_{i}\right)}{a(\phi )}g\right)-\frac{b^{''}\left(\eta_{i}\right)}{a(\phi )}x_{i}^{T}g\right]h\left(g,D_{i}\right)\;\;=\sum_{i=1}^{N}\;\;\left(-{\left\{p_{\theta }\left(y_{i},D_{i}|x_{i}\right)\right\}}^{-2}\right)\left[\sum_{g\in 𝒢}\;\;f_{\theta }\left(y_{i}|x_{i},g\right)\frac{y_{i}-b^{'}\left(\eta_{i}\right)}{a(\phi )}x_{i}^{T}h\left(g,D_{i}\right)\right]\left[\sum_{g\in 𝒢}\;\;f_{\theta }\left(y_{i}|x_{i},g\right)\frac{y_{i}-b^{'}\left(\eta_{i}\right)}{a(\phi )}gh\left(g,D_{i}\right)\right]\;\;+{\left\{p_{\theta }\left(y_{i},D_{i}|x_{i}\right)\right\}}^{-1}\sum_{g\in 𝒢}\;\;f_{\theta }\left(y_{i}|x_{i},g\right)\left[\frac{{\left(y_{i}-b^{'}\left(\eta_{i}\right)\right)}^{2}}{[a(\phi ){]}^{2}}-\frac{b^{''}\left(\eta_{i}\right)}{a(\phi )}\right]x_{i}^{T}gh\left(g,D_{i}\right)$$

$$\frac{\partial^{2}l_{y,D}(\alpha ,\beta ,\phi )}{\partial \alpha^{T}\partial \phi }=\frac{\partial \frac{\partial l_{y,D}(\alpha ,\beta ,\phi )}{\partial \alpha^{T}}}{\partial \phi }\;\;=\frac{\partial \left[\sum_{i=1}^{N}\;\;{\left\{p_{\theta }\left(y_{i},D_{i}|x_{i}\right)\right\}}^{-1}\sum_{g\in 𝒢}\;\;f_{\theta }\left(y_{i}|x_{i},g\right)\frac{y_{i}-b^{'}\left(\eta_{i}\right)}{a(\phi )}x_{i}^{T}h\left(g,D_{i}\right)\right]}{\partial \phi }\;\;=\sum_{i=1}^{N}\;\;\frac{\partial \left[{\left\{p_{\theta }\left(y_{i},D_{i}|x_{i}\right)\right\}}^{-1}\sum_{g\in 𝒢}\;\;f_{\theta }\left(y_{i}|x_{i},g\right)\frac{y_{i}-b^{'}\left(\eta_{i}\right)}{a(\phi )}x_{i}^{T}h\left(g,D_{i}\right)\right]}{\partial \phi }\;\;=\sum_{i=1}^{N}\;\;\left[\sum_{g\in 𝒢}\;\;f_{\theta }\left(y_{i}|x_{i},g\right)\frac{y_{i}-b^{'}\left(\eta_{i}\right)}{a(\phi )}x_{i}^{T}h\left(g,D_{i}\right)\right]\frac{\partial {\left\{p_{\theta }\left(y_{i},D_{i}|x_{i}\right)\right\}}^{-1}}{\partial \phi }+{\left\{p_{\theta }\left(y_{i},D_{i}|x_{i}\right)\right\}}^{-1}\frac{\partial \left[\sum_{g\in 𝒢}\;\;f_{\theta }\left(y_{i}|x_{i},g\right)\frac{y_{i}-b^{'}\left(\eta_{i}\right)}{a(\phi )}x_{i}^{T}h\left(g,D_{i}\right)\right]}{\partial \phi }\;\;=\sum_{i=1}^{N}\;\;\left[\sum_{g\in 𝒢}\;\;f_{\theta }\left(y_{i}|x_{i},g\right)\frac{y_{i}-b^{'}\left(\eta_{i}\right)}{a(\phi )}x_{i}^{T}h\left(g,D_{i}\right)\right]\left[\left(-{\left\{p_{\theta }\left(y_{i},D_{i}|x_{i}\right)\right\}}^{-2}\right)\sum_{g\in 𝒢}\;\;f_{\theta }\left(y_{i}|x_{i},g\right)\right.\;\left.\left(-\frac{y_{i}\eta_{i}-b\left(\eta_{i}\right)}{[a(\phi ){]}^{2}}a^{'}(\phi )+\frac{\partial c\left(y_{i},\phi \right)}{\partial \phi }\right)h\left(g,D_{i}\right)\right]+{\left\{p_{\theta }\left(y_{i},D_{i}|x_{i}\right)\right\}}^{-1}\sum_{g\in 𝒢}\;\;\frac{\partial \left[f_{\theta }\left(y_{i}|x_{i},g\right)\frac{y_{i}-b^{'}\left(\eta_{i}\right)}{a(\phi )}x_{i}^{T}\right]}{\partial \phi }h\left(g,D_{i}\right)\;\;=\sum_{i=1}^{N}\;\;\left(-{\left\{p_{\theta }\left(y_{i},D_{i}|x_{i}\right)\right\}}^{-2}\right)\left[\sum_{g\in 𝒢}\;\;f_{\theta }\left(y_{i}|x_{i},g\right)\frac{y_{i}-b^{'}\left(\eta_{i}\right)}{a(\phi )}x_{i}^{T}h\left(g,D_{i}\right)\right]\left[\sum_{g\in 𝒢}\;\;f_{\theta }(y_{i}|x_{i},g)(-\frac{y_{i}\eta_{i}-b(\eta_{i})}{[a(\phi ){]}^{2}}a^{'}(\phi )+\frac{\partial c(y_{i},\phi )}{\partial \phi })h\left(g,D_{i}\right.\right.\;\;+{\left\{p_{\theta }\left(y_{i},D_{i}|x_{i}\right)\right\}}^{-1}\sum_{g\in 𝒢}\;\;f_{\theta }\left(y_{i}|x_{i},g\right)\left[\frac{\partial \left[\frac{y_{i}\eta_{i}-b\left(\eta_{i}\right)}{a(\phi )}+c\left(y_{i},\phi \right)\right]}{\partial \phi }\left(\frac{y_{i}-b^{'}\left(\eta_{i}\right)}{a(\phi )}x_{i}^{T}\right)+\frac{\partial \left[\frac{y_{i}-b^{'}\left(\eta_{i}\right)}{a(\phi )}x_{i}^{T}\right]}{\partial \phi }\right]h\left(g,D_{i}\right)\;\;=\sum_{i=1}^{N}\;\;\left(-{\left\{p_{\theta }\left(y_{i},D_{i}|x_{i}\right)\right\}}^{-2}\right)\left[\sum_{g\in 𝒢}\;\;f_{\theta }\left(y_{i}|x_{i},g\right)\frac{y_{i}-b^{'}\left(\eta_{i}\right)}{a(\phi )}x_{i}^{T}h\left(g,D_{i}\right)\right]\left[\sum_{g\in 𝒢}\;\;f_{\theta }(y_{i}|x_{i},g)(-\frac{y_{i}\eta_{i}-b(\eta_{i})}{[a(\phi ){]}^{2}}a^{'}(\phi )+\frac{\partial c(y_{i},\phi )}{\partial \phi })h\left(g,D_{i}\right.\right.\;\;+{\left\{p_{\theta }\left(y_{i},D_{i}|x_{i}\right)\right\}}^{-1}\sum_{g\in 𝒢}\;\;f_{\theta }\left(y_{i}|x_{i},g\right)\left[\left(-\frac{y_{i}\eta_{i}-b\left(\eta_{i}\right)}{[a(\phi ){]}^{2}}a^{'}(\phi )+\frac{\partial c\left(y_{i},\phi \right)}{\partial \phi }\right)\left(\frac{y_{i}-b^{'}\left(\eta_{i}\right)}{a(\phi )}\right)-\frac{y_{i}-b^{'}\left(\eta_{i}\right)}{[a(\phi ){]}^{2}}a^{'}(\phi )\right]x_{i}^{T}h\left(g,D_{i}\right)$$

$$\frac{\partial^{2}l_{y,D}(\alpha ,\beta ,\phi )}{\partial \beta^{T}\partial \beta }=\frac{\partial \frac{\partial l_{y,D}(\alpha ,\beta ,\phi )}{\partial \beta^{T}}}{\partial \beta }\;\;=\frac{\partial \left[\sum_{i=1}^{N}\;\;{\left\{p_{\theta }\left(y_{i},D_{i}|x_{i}\right)\right\}}^{-1}\sum_{g\in 𝒢}\;\;f_{\theta }\left(y_{i}|x_{i},g\right)\frac{y_{i}-b^{'}\left(\eta_{i}\right)}{a(\phi )}g^{T}h\left(g,D_{i}\right)\right]}{\partial \beta }\;\;=\sum_{i=1}^{N}\;\;\frac{\partial \left[{\left\{p_{\theta }\left(y_{i},D_{i}|x_{i}\right)\right\}}^{-1}\sum_{g\in 𝒢}\;\;f_{\theta }\left(y_{i}|x_{i},g\right)\frac{y_{i}-b^{'}\left(\eta_{i}\right)}{a(\phi )}g^{T}h\left(g,D_{i}\right)\right]}{\partial \beta }\;\;=\sum_{i=1}^{N}\;\;\left[\sum_{g\in 𝒢}\;\;f_{\theta }\left(y_{i}|x_{i},g\right)\frac{y_{i}-b^{'}\left(\eta_{i}\right)}{a(\phi )}g^{T}h\left(g,D_{i}\right)\right]\frac{\partial {\left\{p_{\theta }\left(y_{i},D_{i}|x_{i}\right)\right\}}^{-1}}{\partial \beta }+{\left\{p_{\theta }\left(y_{i},D_{i}|x_{i}\right)\right\}}^{-1}\frac{\partial \left[\sum_{g\in 𝒢}\;\;f_{\theta }\left(y_{i}|x_{i},g\right)\frac{y_{i}-b^{'}\left(\eta_{i}\right)}{a(\phi )}g^{T}h\left(g,D_{i}\right)\right.}{\partial \beta }\;\;=\sum_{i=1}^{N}\;\;\left[\sum_{g\in 𝒢}\;\;f_{\theta }\left(y_{i}|x_{i},g\right)\frac{y_{i}-b^{'}\left(\eta_{i}\right)}{a(\phi )}g^{T}h\left(g,D_{i}\right)\right]\left[\left(-{\left\{p_{\theta }\left(y_{i},D_{i}|x_{i}\right)\right\}}^{-2}\right)\sum_{g\in 𝒢}\;\;f_{\theta }\left(y_{i}|x_{i},g\right)\frac{y_{i}-b^{'}\left(\eta_{i}\right)}{a(\phi )}gh\left(g,D_{i}\right)\right]\;\;+{\left\{p_{\theta }\left(y_{i},D_{i}|x_{i}\right)\right\}}^{-1}\sum_{g\in 𝒢}\;\;\frac{\partial \left[f_{\theta }\left(y_{i}|x_{i},g\right)\frac{y_{i}-b^{'}\left(\eta_{i}\right)}{a(\phi )}g^{T}\right]}{\partial \beta }h\left(g,D_{i}\right)\;\;=\sum_{i=1}^{N}\;\;\left(-{\left\{p_{\theta }\left(y_{i},D_{i}|x_{i}\right)\right\}}^{-2}\right)\left[\sum_{g\in 𝒢}\;\;f_{\theta }\left(y_{i}|x_{i},g\right)\frac{y_{i}-b^{'}\left(\eta_{i}\right)}{a(\phi )}g^{T}h\left(g,D_{i}\right)\right]\left[\sum_{g\in 𝒢}\;\;f_{\theta }\left(y_{i}|x_{i},g\right)\frac{y_{i}-b^{'}\left(\eta_{i}\right)}{a(\phi )}gh\left(g,D_{i}\right)\right]\;\;+{\left\{p_{\theta }\left(y_{i},D_{i}|x_{i}\right)\right\}}^{-1}\sum_{g\in 𝒢}\;\;f_{\theta }\left(y_{i}|x_{i},g\right)\left[\left(\frac{y_{i}-b^{'}\left(\eta_{i}\right)}{a(\phi )}g^{T}\right)\frac{\partial \left[\frac{y_{i}\eta_{i}-b\left(\eta_{i}\right)}{a(\phi )}+c\left(y_{i},\phi \right)\right]}{\partial \beta }+\frac{\partial \left[\frac{y_{i}-b^{'}\left(\eta_{i}\right)}{a(\phi )}g^{T}\right]}{\partial \beta }\right]h\left(g,D_{i}\right)\;\;=\sum_{i=1}^{N}\;\;\left(-{\left\{p_{\theta }\left(y_{i},D_{i}|x_{i}\right)\right\}}^{-2}\right)\left[\sum_{g\in 𝒢}\;\;f_{\theta }\left(y_{i}|x_{i},g\right)\frac{y_{i}-b^{'}\left(\eta_{i}\right)}{a(\phi )}g^{T}h\left(g,D_{i}\right)\right]\left[\sum_{g\in 𝒢}\;\;f_{\theta }\left(y_{i}|x_{i},g\right)\frac{y_{i}-b^{'}\left(\eta_{i}\right)}{a(\phi )}gh\left(g,D_{i}\right)\right]\;\;+{\left\{p_{\theta }\left(y_{i},D_{i}|x_{i}\right)\right\}}^{-1}\sum_{g\in 𝒢}\;\;f_{\theta }\left(y_{i}|x_{i},g\right)\left[\left(\frac{y_{i}-b^{'}\left(\eta_{i}\right)}{a(\phi )}g^{T}\right)\left(\frac{y_{i}-b^{'}\left(\eta_{i}\right)}{a(\phi )}g\right)-\frac{b^{''}\left(\eta_{i}\right)}{a(\phi )}g^{T}g\right]h\left(g,D_{i}\right)\;\;=\sum_{i=1}^{N}\;\;\left(-{\left\{p_{\theta }\left(y_{i},D_{i}|x_{i}\right)\right\}}^{-2}\right)\left[\sum_{g\in 𝒢}\;\;f_{\theta }\left(y_{i}|x_{i},g\right)\frac{y_{i}-b^{'}\left(\eta_{i}\right)}{a(\phi )}g^{T}h\left(g,D_{i}\right)\right]\left[\sum_{g\in 𝒢}\;\;f_{\theta }\left(y_{i}|x_{i},g\right)\frac{y_{i}-b^{'}\left(\eta_{i}\right)}{a(\phi )}gh\left(g,D_{i}\right)\right]\;\;+{\left\{p_{\theta }\left(y_{i},D_{i}|x_{i}\right)\right\}}^{-1}\sum_{g\in 𝒢}\;\;f_{\theta }\left(y_{i}|x_{i},g\right)\left[\frac{{\left(y_{i}-b^{'}\left(\eta_{i}\right)\right)}^{2}}{[a(\phi ){]}^{2}}-\frac{b^{''}\left(\eta_{i}\right)}{a(\phi )}\right]g^{T}gh\left(g,D_{i}\right)$$

$$\frac{\partial^{2}l_{y,D}(\alpha ,\beta ,\phi )}{\partial \beta^{T}\partial \phi }=\frac{\partial \frac{\partial l_{y,D}(\alpha ,\beta ,\phi )}{\partial \beta^{T}}}{\partial \phi }\;\;=\frac{\partial \left[\sum_{i=1}^{N}\;\;{\left\{p_{\theta }\left(y_{i},D_{i}|x_{i}\right)\right\}}^{-1}\sum_{g\in 𝒢}\;\;f_{\theta }\left(y_{i}|x_{i},g\right)\frac{y_{i}-b^{'}\left(\eta_{i}\right)}{a(\phi )}g^{T}h\left(g,D_{i}\right)\right]}{\partial \phi }\;\;=\sum_{i=1}^{N}\;\;\frac{\partial \left[{\left\{p_{\theta }\left(y_{i},D_{i}|x_{i}\right)\right\}}^{-1}\sum_{g\in 𝒢}\;\;f_{\theta }\left(y_{i}|x_{i},g\right)\frac{y_{i}-b^{'}\left(\eta_{i}\right)}{a(\phi )}g^{T}h\left(g,D_{i}\right)\right]}{\partial \phi }\;\;=\sum_{i=1}^{N}\;\;\left[\sum_{g\in 𝒢}\;\;f_{\theta }\left(y_{i}|x_{i},g\right)\frac{y_{i}-b^{'}\left(\eta_{i}\right)}{a(\phi )}g^{T}h\left(g,D_{i}\right)\right]\frac{\partial {\left\{p_{\theta }\left(y_{i},D_{i}|x_{i}\right)\right\}}^{-1}}{\partial \phi }+{\left\{p_{\theta }\left(y_{i},D_{i}|x_{i}\right)\right\}}^{-1}\frac{\partial \sum_{g\in 𝒢}\;\;f_{\theta }\left(y_{i}|x_{i},g\right)\frac{y_{i}-b^{'}\left(\eta_{i}\right)}{a(\phi )}g^{T}h\left(g,D_{i}\right)}{\partial \phi }\;\;=\sum_{i=1}^{N}\;\;\left\{\sum_{g\in 𝒢}\;\;f_{\theta }\left(y_{i}|x_{i},g\right)\frac{y_{i}-b^{'}\left(\eta_{i}\right)}{a(\phi )}g^{T}h\left(g,D_{i}\right)\right\}\left(-{\left\{p_{\theta }\left(y_{i},D_{i}|x_{i}\right)\right\}}^{-2}\right)\;\left\{\sum_{g\in 𝒢}\;\;f_{\theta }\left(y_{i}|x_{i},g\right)\left[-\frac{y_{i}\eta_{i}-b\left(\eta_{i}\right)}{[a(\phi ){]}^{2}}+\frac{\partial c\left(y_{i},\phi \right)}{\partial \phi }\right]h\left(g,D_{i}\right)\right\}+{\left\{p_{\theta }\left(y_{i},D_{i}|x_{i}\right)\right\}}^{-1}\sum_{g\in 𝒢}\;\;\frac{\partial f_{\theta }\left(y_{i}|x_{i},g\right)\frac{y_{i}-b^{'}\left(\eta_{i}\right)}{a(\phi )}g^{T}}{\partial \phi }h\left(g,D_{i}\right)\;\;=\sum_{i=1}^{N}\;\;\left(-{\left\{p_{\theta }\left(y_{i},D_{i}|x_{i}\right)\right\}}^{-2}\right)\left[\sum_{g\in 𝒢}\;\;f_{\theta }\left(y_{i}|x_{i},g\right)\frac{y_{i}-b^{'}\left(\eta_{i}\right)}{a(\phi )}g^{T}h\left(g,D_{i}\right)\right]\left\{\sum_{g\in 𝒢}\;\;f_{\theta }(y_{i}|x_{i},g)[-\frac{y_{i}\eta_{i}-b(\eta_{i})}{[a(\phi ){]}^{2}}a^{'}(\phi )+\frac{\partial c(y_{i},\phi )}{\partial \phi }]h\left(g,D_{i}\right.\right.\;\;+{\left\{p_{\theta }\left(y_{i},D_{i}|x_{i}\right)\right\}}^{-1}\sum_{g\in 𝒢}\;\;f_{\theta }\left(y_{i}|x_{i},g\right)\left[\left(\frac{y_{i}-b^{'}\left(\eta_{i}\right)}{a(\phi )}g^{T}\right)\frac{\partial \left[\frac{y_{i}\eta_{i}-b\left(\eta_{i}\right)}{a(\phi )}+c\left(y_{i},\phi \right)\right]}{\partial \phi }+\frac{\partial \left[\frac{y_{i}-b^{'}\left(\eta_{i}\right)}{a(\phi )}g^{T}\right]}{\partial \phi }\right]h\left(g,D_{i}\right)\;\;=\sum_{i=1}^{N}\;\;\left(-{\left\{p_{\theta }\left(y_{i},D_{i}|x_{i}\right)\right\}}^{-2}\right)\left[\sum_{g\in 𝒢}\;\;f_{\theta }\left(y_{i}|x_{i},g\right)\frac{y_{i}-b^{'}\left(\eta_{i}\right)}{a(\phi )}g^{T}h\left(g,D_{i}\right)\right]\left\{\sum_{g\in 𝒢}\;\;f_{\theta }(y_{i}|x_{i},g)[-\frac{y_{i}\eta_{i}-b(\eta_{i})}{[a(\phi ){]}^{2}}a^{'}(\phi )+\frac{\partial c(y_{i},\phi )}{\partial \phi }]h\left(g,D_{i}\right.\right.\;\;+{\left\{p_{\theta }\left(y_{i},D_{i}|x_{i}\right)\right\}}^{-1}\sum_{g\in 𝒢}\;\;f_{\theta }\left(y_{i}|x_{i},g\right)\left(\left(\frac{y_{i}-b^{'}\left(\eta_{i}\right)}{a(\phi )}g^{T}\right)\left(-\frac{y_{i}\eta_{i}-b\left(\eta_{i}\right)}{[a(\phi ){]}^{2}}a^{'}(\phi )+\frac{\partial c\left(y_{i},\phi \right)}{\partial \phi }\right)-\left(\frac{y_{i}-b^{'}\left(\eta_{i}\right)}{[a(\phi ){]}^{2}}a^{'}(\phi )g^{T}\right)\right)h\left(g,D_{i}\right)$$

$$\frac{\partial^{2}l_{y,D}(\alpha ,\beta ,\phi )}{\partial \phi^{2}}=\frac{\partial \frac{\partial l_{y,D}(\alpha ,\beta ,\phi )}{\partial \phi }}{\partial \phi }\;\;=\frac{\partial \sum_{i=1}^{N}\;\;{\left\{p_{\theta }\left(y_{i},D_{i}|x_{i}\right)\right\}}^{-1}\sum_{g\in 𝒢}\;\;f_{\theta }\left(y_{i}|x_{i},g\right)\left[-\frac{y_{i}\eta_{i}-b\left(\eta_{i}\right)}{[a(\phi ){]}^{2}}a^{'}(\phi )+\frac{\partial c\left(y_{i},\phi \right)}{\partial \phi }\right]h\left(g,D_{i}\right)}{\partial \phi }\;\;=\sum_{i=1}^{N}\;\;\frac{\partial {\left\{p_{\theta }\left(y_{i},D_{i}|x_{i}\right)\right\}}^{-1}\sum_{g\in 𝒢}\;\;f_{\theta }\left(y_{i}|x_{i},g\right)\left[-\frac{y_{i}\eta_{i}-b\left(\eta_{i}\right)}{[a(\phi ){]}^{2}}a^{'}(\phi )+\frac{\partial c\left(y_{i},\phi \right)}{\partial \phi }\right]h\left(g,D_{i}\right)}{\partial \phi }\;\;=\sum_{i=1}^{N}\;\;\left[\frac{\partial {\left\{p_{\theta }\left(y_{i},D_{i}|x_{i}\right)\right\}}^{-1}}{\partial \phi }\sum_{g\in 𝒢}\;\;f_{\theta }\left(y_{i}|x_{i},g\right)\left[-\frac{y_{i}\eta_{i}-b\left(\eta_{i}\right)}{[a(\phi ){]}^{2}}a^{'}(\phi )+\frac{\partial c\left(y_{i},\phi \right)}{\partial \phi }\right]h\left(g,D_{i}\right)\right]\;\left.+{\left\{p_{\theta }\left(y_{i},D_{i}|x_{i}\right)\right\}}^{-1}\frac{\partial \sum_{g\in 𝒢}\;\;f_{\theta }\left(y_{i}|x_{i},g\right)\left[-\frac{y_{i}\eta_{i}-b\left(\eta_{i}\right)}{[a(\phi ){]}^{2}}a^{'}(\phi )+\frac{\partial c\left(y_{i},\phi \right)}{\partial \phi }\right]h\left(g,D_{i}\right)}{\partial \phi }\right]\;\;=\sum_{i=1}^{N}\;\;\left[-{\left\{p_{\theta }\left(y_{i},D_{i}|x_{i}\right)\right\}}^{-2}\frac{\partial p_{\theta }\left(y_{i},D_{i}|x_{i}\right)}{\partial \phi }\left(\sum_{g\in 𝒢}\;\;f_{\theta }\left(y_{i}|x_{i},g\right)\left[-\frac{y_{i}\eta_{i}-b\left(\eta_{i}\right)}{[a(\phi ){]}^{2}}a^{'}(\phi )+\frac{\partial c\left(y_{i},\phi \right)}{\partial \phi }\right]h\left(g,D_{i}\right)\right)\right.\;\left.+{\left\{p_{\theta }\left(y_{i},D_{i}|x_{i}\right)\right\}}^{-1}\sum_{g\in 𝒢}\;\;\frac{\partial f_{\theta }\left(y_{i}|x_{i},g\right)\left[-\frac{y_{i}\eta_{i}-b\left(\eta_{i}\right)}{[a(\phi ){]}^{2}}a^{'}(\phi )+\frac{\partial c\left(y_{i},\phi \right)}{\partial \phi }\right]}{\partial \phi }h\left(g,D_{i}\right)\right]\;\;=\sum_{i=1}^{N}\;\;\left[-{\left\{p_{\theta }\left(y_{i},D_{i}|x_{i}\right)\right\}}^{-2}{\left(\sum_{g\in 𝒢}\;\;f_{\theta }\left(y_{i}|x_{i},g\right)\left[-\frac{y_{i}\eta_{i}-b\left(\eta_{i}\right)}{[a(\phi ){]}^{2}}a^{'}(\phi )+\frac{\partial c\left(y_{i},\phi \right)}{\partial \phi }\right]h\left(g,D_{i}\right)\right)}^{2}\right.\;\;+{\left\{p_{\theta }\left(y_{i},D_{i}|x_{i}\right)\right\}}^{-1}\sum_{g\in 𝒢}\;\;\left(\frac{\partial f_{\theta }\left(y_{i}|x_{i},g\right)}{\partial \phi }\right)\left[-\frac{y_{i}\eta_{i}-b\left(\eta_{i}\right)}{[a(\phi ){]}^{2}}a^{'}(\phi )+\frac{\partial c\left(y_{i},\phi \right)}{\partial \phi }\right]\;\left.\left.+f_{\theta }\left(y_{i}|x_{i},g\right)\frac{\partial \left[-\frac{y_{i}\eta_{i}-b\left(\eta_{i}\right)}{[a(\phi ){]}^{2}}a^{'}(\phi )+\frac{\partial c\left(y_{i},\phi \right)}{\partial \phi }\right]}{\partial \phi }\right)h\left(g,D_{i}\right)\right]\;\;=\sum_{i=1}^{N}\;\;\left[-{\left\{p_{\theta }\left(y_{i},D_{i}|x_{i}\right)\right\}}^{-2}{\left(\sum_{g\in 𝒢}\;\;f_{\theta }\left(y_{i}|x_{i},g\right)\left[-\frac{y_{i}\eta_{i}-b\left(\eta_{i}\right)}{[a(\phi ){]}^{2}}a^{'}(\phi )+\frac{\partial c\left(y_{i},\phi \right)}{\partial \phi }\right]h\left(g,D_{i}\right)\right)}^{2}\right.\;\;+{\left\{p_{\theta }\left(y_{i},D_{i}|x_{i}\right)\right\}}^{-1}\sum_{g\in 𝒢}\;\;f_{\theta }\left(y_{i}|x_{i},g\right)\left\{{\left[-\frac{y_{i}\eta_{i}-b\left(\eta_{i}\right)}{[a(\phi ){]}^{2}}a^{'}(\phi )+\frac{\partial c\left(y_{i},\phi \right)}{\partial \phi }\right]}^{2}\right.\;\left.\left.+\left(y_{i}\eta_{i}-b\left(\eta_{i}\right)\right)\left(\frac{2{\left[a^{'}(\phi )\right]}^{2}}{[a(\phi ){]}^{3}}-\frac{a^{''}(\phi )}{[a(\phi ){]}^{2}}\right)+\frac{\partial^{2}c\left(y_{i},\phi \right)}{\partial \phi^{2}}\right\}h\left(g,D_{i}\right)\right]$$

The following terms can be calculated by matrix transposition.

$$\frac{\partial^{2}l_{y,D}(\alpha ,\beta ,\phi )}{\partial \beta^{T}\partial \alpha }={\left(\frac{\partial^{2}l_{y,D}(\alpha ,\beta ,\phi )}{\partial \alpha^{T}\partial \beta }\right)}^{T};\;\frac{\partial^{2}l_{y,D}(\alpha ,\beta ,\phi )}{\partial \phi \partial \alpha }={\left(\frac{\partial^{2}l_{y,D}(\alpha ,\beta ,\phi )}{\partial \alpha^{T}\partial \phi }\right)}^{T};\;\frac{\partial^{2}l_{y,D}(\alpha ,\beta ,\phi )}{\partial \phi \partial \beta }={\left(\frac{\partial^{2}l_{y,D}(\alpha ,\beta ,\phi )}{\partial \beta^{T}\partial \phi }\right)}^{T}.$$

## Appendix D Evaluation of Score Function at Constrained MLE when Parameters are (α,β,ϕ)

We evaluated the score function at the constrained MLE under the null hypothesis, i.e. $\tilde{\theta }=(\tilde{\alpha },0,\tilde{\phi })$. Under the null hypothesis, We have

$$p_{\tilde{\theta }}\left(y_{i},D_{i}|x_{i}\right)=\sum_{g\in 𝒢}\;f_{\tilde{\theta }}\left(y_{i}|x_{i},g\right)h\left(g,D_{i}\right)=\sum_{g\in 𝒢}\;f_{\tilde{\theta }}\left(y_{i}|x_{i}\right)h\left(g,D_{i}\right)=f_{\tilde{\theta }}\left(y_{i}|x_{i}\right)\sum_{g\in 𝒢}\;h\left(g,D_{i}\right)$$

and ${\tilde{\eta }}_{i}=\tilde{\eta }\left(x_{i},g\right)=\tilde{\eta }\left(x_{i}\right)=\tilde{\alpha }x_{i}^{T}$. Note that under the null hypothesis, the term ${\tilde{\eta }}_{i}=\tilde{\alpha }x_{i}^{T}$ does not include g. Thus,

$$\begin{array}{ll} & s_{y,D}(\tilde{\alpha },0,\tilde{\phi })\\ & \;=\sum_{i=1}^{N}\;\;\left[{\left\{p_{\tilde{\theta }}\left(y_{i},D_{i}|x_{i}\right)\right\}}^{-1}\sum_{g\in 𝒢}\;\;f_{\tilde{\theta }}\left(y_{i}|x_{i},g\right)\left[\begin{array}{c} \frac{y_{i}-b^{'}\left({\tilde{\eta }}_{i}\right)}{a(\tilde{\phi })}x_{i}^{T}\\ \frac{y_{i}-b^{'}\left({\tilde{\eta }}_{i}\right)}{a(\tilde{\phi })}g^{T}\\ -\frac{y_{i}{\tilde{\eta }}_{i}-b\left(\tilde{\eta_{i}}\right)}{[a(\tilde{\phi }){]}^{2}}a^{'}(\tilde{\phi })+{\left.\frac{\partial c\left(y_{i},\phi \right)}{\partial \phi }\right|}_{\phi =\tilde{\phi }} \end{array}\right]h\left(g,D_{i}\right)\right]\\ & \;=\sum_{i=1}^{N}\;\;\left[{\left\{f_{\tilde{\theta }}\left(y_{i}|x_{i}\right)\sum_{g\in 𝒢}\;\;h\left(g,D_{i}\right)\right\}}^{-1}f_{\tilde{\theta }}\left(y_{i}|x_{i}\right)\sum_{g\in 𝒢}\;\;\left[\begin{array}{c} \frac{y_{i}-b^{'}\left({\tilde{\eta }}_{i}\right)}{a(\tilde{\phi })}x_{i}^{T}\\ \frac{y_{i}-b^{'}\left({\tilde{\eta }}_{i}\right)}{a(\tilde{\phi })}g^{T}\\ -\frac{y_{i}{\tilde{\eta }}_{i}-b\left(\tilde{\eta_{i}}\right)}{[a(\tilde{\phi }){]}^{2}}a^{'}(\tilde{\phi })+{\left.\frac{\partial c\left(y_{i},\phi \right)}{\partial \phi }\right|}_{\phi =\tilde{\phi }} \end{array}\right]h\left(g,D_{i}\right)\right]\\ & \;=\sum_{i=1}^{N}\;\;\left[{\left\{\sum_{g\in 𝒢}\;\;h\left(g,D_{i}\right)\right\}}^{-1}\sum_{g\in 𝒢}\;\;\left[\begin{array}{c} \frac{y_{i}-b^{'}\left({\tilde{\eta }}_{i}\right)}{a(\tilde{\phi })}x_{i}^{T}\\ \frac{y_{i}-b^{'}\left({\tilde{\eta }}_{i}\right)}{a(\tilde{\phi })}g^{T}\\ -\frac{y_{i}{\tilde{\eta }}_{i}-b\left(\tilde{\eta_{i}}\right)}{[a(\tilde{\phi }){]}^{2}}a^{'}(\tilde{\phi })+{\left.\frac{\partial c\left(y_{i},\phi \right)}{\partial \phi }\right|}_{\phi =\tilde{\phi }} \end{array}\right]h\left(g,D_{i}\right)\right]\\ & \left.=\sum_{i=1}^{N}\;\;\left[\begin{array}{c} {\left\{\sum_{g\in 𝒢}\;\;h\left(g,D_{i}\right)\right\}}^{-1}\sum_{g\in 𝒢}\;\;\frac{y_{i}-b^{'}\left({\tilde{\eta }}_{i}\right)}{a(\tilde{\phi })}x_{i}^{T}\left\{h\left(g,D_{i}\right)\right\}\\ {\left\{\sum_{g\in 𝒢}\;\;h\left(g,D_{i}\right)\right\}}^{-1}\sum_{g\in 𝒢}\;\;\frac{y_{i}-b^{'}\left({\tilde{\eta }}_{i}\right)}{a(\tilde{\phi })}g^{T}\left\{h\left(g,D_{i}\right)\right\}\\ {\left\{\sum_{g\in 𝒢}\;\;h\left(g,D_{i}\right)\right\}}^{-1}\sum_{g\in 𝒢}\;\;\left(-\frac{y_{i}{\tilde{\eta }}_{i}-b\left(\tilde{\eta }{\tilde{\eta }}_{i}\right)}{[a(\tilde{\phi }){]}^{2}}a^{'}(\tilde{\phi })+{\left.\frac{\partial c\left(y_{i},\phi \right)}{\partial \phi }\right|}_{\phi =\tilde{\phi }}\right)\left\{h\left(g,D_{i}\right)\right\} \end{array}\right]\right]\\ & \left.=\sum_{i=1}^{N}\;\;\left[\begin{array}{c} {\left\{\sum_{g\in 𝒢}\;\;h\left(g,D_{i}\right)\right\}}^{-1}\frac{y_{i}-b^{'}\left({\tilde{\eta }}_{i}\right)}{a(\tilde{\phi })}x_{i}^{T}\left\{\sum_{g\in 𝒢}\;\;h\left(g,D_{i}\right)\right\}\\ {\left\{\sum_{g\in 𝒢}\;\;h\left(g,D_{i}\right)\right\}}^{-1}\sum_{g\in 𝒢}\;\;\frac{y_{i}-b^{'}\left({\tilde{\eta }}_{i}\right)}{a(\tilde{\phi })}g^{T}\left\{h\left(g,D_{i}\right)\right\}\\ {\left\{\sum_{g\in 𝒢}\;\;h\left(g,D_{i}\right)\right\}}^{-1}\left(-\frac{y_{i}{\tilde{\eta }}_{i}-{\tilde{\eta }}_{i}^{2}/2}{[a(\tilde{\phi }){]}^{2}}a^{'}(\tilde{\phi })+{\left.\frac{\partial c\left(y_{i},\phi \right)}{\partial \phi }\right|}_{\phi =\tilde{\phi }}\right)\left\{\sum_{g\in 𝒢}\;\;h\left(g,D_{i}\right)\right\} \end{array}\right]\right]\\ & \;=\left[\begin{array}{c} \sum_{i=1}^{N}\;\;\frac{y_{i}-b^{'}\left({\tilde{\eta }}_{i}\right)}{a(\tilde{\phi })}x_{i}^{T}\\ \sum_{i=1}^{N}\;\;\left[\frac{y_{i}-b^{'}\left({\tilde{\eta }}_{i}\right)}{a(\tilde{\phi })}{\left\{\sum_{g\in 𝒢}\;\;h\left(g,D_{i}\right)\right\}}^{-1}\sum_{g\in 𝒢}\;\;g^{T}\left\{h\left(g,D_{i}\right)\right\}\right]\\ \sum_{i=1}^{N}\;\;\left(-\frac{y_{i}{\tilde{\eta }}_{i}-b\left({\tilde{\eta }}_{i}\right)}{[a(\tilde{\phi }){]}^{2}}a^{'}(\tilde{\phi })+{\left.\frac{\partial c\left(y_{i},\phi \right)}{\partial \phi }\right|}_{\phi =\tilde{\phi }}\right) \end{array}\right]\\ & \left.=\left[\begin{array}{c} 0\\ \sum_{i=1}^{N}\;\;\frac{y_{i}-b^{'}\left(\tilde{\alpha }x_{i}^{T}\right)}{a(\tilde{\phi })}\\ 0 \end{array}\right]\left(g^{T}|D_{i}\right)\right], \end{array}$$

where $E\left(g^{T}|D_{i}\right)={\left\{\sum_{g\in 𝒢}\;h\left(g,D_{i}\right)\right\}}^{-1}\sum_{g\in 𝒢}\;g^{T}h\left(g,D_{i}\right)=\left(\sum_{g\in 𝒢}\;g^{T}h\left(g,D_{i}\right)\right)/\left(\sum_{g\in 𝒢}\;h\left(g,D_{i}\right)\right)$ is the posterior expectation of the genotype of individual i given sequencing data $D_{i}$.

## Appendix E Evaluation of Observed Information Matrix at Constrained MLE when Parameters are (α,β,ϕ)

$$\begin{array}{ll} & {\left.\frac{\partial^{2}l_{y,D}(\alpha ,\beta ,\phi )}{\partial \alpha^{T}\partial \alpha }\right|}_{\tilde{\theta }}\\ & \;=\sum_{i=1}^{N}\;\;\left(-{\left\{p_{\theta }\left(y_{i},D_{i}|x_{i}\right)\right\}}^{-2}\right)\left[\sum_{g\in 𝒢}\;\;f_{\theta }\left(y_{i}|x_{i},g\right)\frac{y_{i}-b^{'}\left({\tilde{\eta }}_{i}\right)}{a(\tilde{\phi })}x_{i}^{T}h\left(g,D_{i}\right)\right]\left[\sum_{g\in 𝒢}\;\;f_{\theta }\left(y_{i}|x_{i},g\right)\frac{y_{i}-b^{'}\left({\tilde{\eta }}_{i}\right)}{a(\tilde{\phi })}x_{i}h\left(g,D_{i}\right)\right]\\ & \;+{\left\{p_{\theta }\left(y_{i},D_{i}|x_{i}\right)\right\}}^{-1}\sum_{g\in 𝒢}\;\;f_{\theta }\left(y_{i}|x_{i},g\right)\left[\frac{{\left(y_{i}-b^{'}\left({\tilde{\eta }}_{i}\right)\right)}^{2}}{[a(\tilde{\phi }){]}^{2}}-\frac{b^{''}\left({\tilde{\eta }}_{i}\right)}{a(\tilde{\phi })}\right]x_{i}^{T}x_{i}h\left(g,D_{i}\right)\\ & \;=\sum_{i=1}^{N}\;\;\left(-{\left\{f_{\theta }\left(y_{i}|x_{i}\right)\sum_{g\in 𝒢}\;\;h\left(g,D_{i}\right)\right\}}^{-2}\right)\left[f_{\theta }\left(y_{i}|x_{i}\right)\frac{y_{i}-b^{'}\left({\tilde{\eta }}_{i}\right)}{a(\tilde{\phi })}x_{i}^{T}\sum_{g\in 𝒢}\;\;h\left(g,D_{i}\right)\right]\left[f_{\theta }\left(y_{i}|x_{i}\right)\frac{y_{i}-b^{'}\left({\tilde{\eta }}_{i}\right)}{a(\tilde{\phi })}x_{i}\sum_{g\in 𝒢}\;\;h\left(g,D_{i}\right)\right]\\ & \;+{\left\{f_{\theta }\left(y_{i}|x_{i}\right)\sum_{g\in 𝒢}\;\;h\left(g,D_{i}\right)\right\}}^{-1}f_{\theta }\left(y_{i}|x_{i}\right)\left[\frac{{\left(y_{i}-b^{'}\left({\tilde{\eta }}_{i}\right)\right)}^{2}}{[a(\tilde{\phi }){]}^{2}}-\frac{b^{''}\left({\tilde{\eta }}_{i}\right)}{a(\tilde{\phi })}\right]x_{i}^{T}x_{i}\sum_{g\in 𝒢}\;\;h\left(g,D_{i}\right)\\ & \;=\sum_{i=1}^{N}\;\;(-1)\left[\frac{y_{i}-b^{'}\left({\tilde{\eta }}_{i}\right)}{a(\tilde{\phi })}x_{i}^{T}\right]\left[\frac{y_{i}-b^{'}\left({\tilde{\eta }}_{i}\right)}{a(\tilde{\phi })}x_{i}\right]+\left[\frac{{\left(y_{i}-b^{'}\left({\tilde{\eta }}_{i}\right)\right)}^{2}}{[a(\tilde{\phi }){]}^{2}}-\frac{b^{''}\left({\tilde{\eta }}_{i}\right)}{a(\tilde{\phi })}\right]x_{i}^{T}x_{i}\\ & \;=-\frac{1}{a\left(\tilde{\phi }\right)}\sum_{i=1}^{N}\;\;b^{''}\left(\tilde{\alpha }x_{i}^{T}\right)x_{i}^{T}x_{i}.\\ & \;\\ & \;\\ & \;\\ & \;\\ & \;\\ & \;\\ & \;\\ & \;\\ & \; \end{array}$$

$${\left.\frac{\partial^{2}l_{y,D}(\alpha ,\beta ,\phi )}{\partial \alpha^{T}\partial \beta }\right|}_{\tilde{\theta }}=\sum_{i=1}^{N}\;\;\left(-{\left\{p_{\theta }\left(y_{i},D_{i}|x_{i}\right)\right\}}^{-2}\right)\left[\sum_{g\in 𝒢}\;\;f_{\theta }\left(y_{i}|x_{i},g\right)\frac{y_{i}-b^{'}\left({\tilde{\eta }}_{i}\right)}{a(\tilde{\phi })}x_{i}^{T}h\left(g,D_{i}\right)\right]\left[\sum_{g\in 𝒢}\;\;f_{\theta }\left(y_{i}|x_{i},g\right)\frac{y_{i}-b^{'}\left({\tilde{\eta }}_{i}\right)}{a(\tilde{\phi })}gh\left(g,D_{i}\right)\right]\;+{\left\{p_{\theta }\left(y_{i},D_{i}|x_{i}\right)\right\}}^{-1}\sum_{g\in 𝒢}\;\;f_{\theta }\left(y_{i}|x_{i},g\right)\left[\frac{{\left(y_{i}-b^{'}\left({\tilde{\eta }}_{i}\right)\right)}^{2}}{[a(\tilde{\phi }){]}^{2}}-\frac{b^{''}\left({\tilde{\eta }}_{i}\right)}{a(\tilde{\phi })}\right]x_{i}^{T}gh\left(g,D_{i}\right)=\sum_{i=1}^{N}\;\;\left(-{\left\{f_{\theta }\left(y_{i}|x_{i}\right)\sum_{g\in 𝒢}\;\;h\left(g,D_{i}\right)\right\}}^{-2}\right)\left[f_{\theta }\left(y_{i}|x_{i}\right)\frac{y_{i}-b^{'}\left({\tilde{\eta }}_{i}\right)}{a(\tilde{\phi })}x_{i}^{T}\sum_{g\in 𝒢}\;\;h\left(g,D_{i}\right)\right]\left[f_{\theta }\left(y_{i}|x_{i}\right)\frac{y_{i}-b^{'}\left({\tilde{\eta }}_{i}\right)}{a(\tilde{\phi })}\sum_{g\in 𝒢}\;\;gh\left(g,D_{i}\right)\right]+{\left\{f_{\theta }\left(y_{i}|x_{i}\right)\sum_{g\in 𝒢}\;\;h\left(g,D_{i}\right)\right\}}^{-1}f_{\theta }\left(y_{i}|x_{i}\right)\left[\frac{{\left(y_{i}-b^{'}\left({\tilde{\eta }}_{i}\right)\right)}^{2}}{[a(\tilde{\phi }){]}^{2}}-\frac{b^{''}\left({\tilde{\eta }}_{i}\right)}{a(\tilde{\phi })}\right]x_{i}^{T}\sum_{g\in 𝒢}\;\;gh\left(g,D_{i}\right)=\sum_{i=1}^{N}\;\;\left(-{\left\{\sum_{g\in 𝒢}\;\;h\left(g,D_{i}\right)\right\}}^{-2}\right)\left[\frac{y_{i}-b^{'}\left({\tilde{\eta }}_{i}\right)}{a(\tilde{\phi })}x_{i}^{T}\sum_{g\in 𝒢}\;\;h\left(g,D_{i}\right)\right]\left[\frac{y_{i}-b^{'}\left({\tilde{\eta }}_{i}\right)}{a(\tilde{\phi })}\sum_{g\in 𝒢}\;\;gh\left(g,D_{i}\right)\right]+{\left\{\sum_{g\in 𝒢}\;\;h\left(g,D_{i}\right)\right\}}^{-1}\left[\frac{{\left(y_{i}-b^{'}\left({\tilde{\eta }}_{i}\right)\right)}^{2}}{[a(\tilde{\phi }){]}^{2}}-\frac{b^{''}\left({\tilde{\eta }}_{i}\right)}{a(\tilde{\phi })}\right]x_{i}^{T}\sum_{g\in 𝒢}\;\;gh\left(g,D_{i}\right)=\sum_{i=1}^{N}\;\;\left(-{\left\{\sum_{g\in 𝒢}\;\;h\left(g,D_{i}\right)\right\}}^{-1}\right)\left[\frac{y_{i}-b^{'}\left({\tilde{\eta }}_{i}\right)}{a(\tilde{\phi })}x_{i}^{T}\right]\left[\frac{y_{i}-b^{'}\left({\tilde{\eta }}_{i}\right)}{a(\tilde{\phi })}\sum_{g\in 𝒢}\;\;gh\left(g,D_{i}\right)\right]+{\left\{\sum_{g\in 𝒢}\;\;h\left(g,D_{i}\right)\right\}}^{-1}\left[\frac{{\left(y_{i}-b^{'}\left({\tilde{\eta }}_{i}\right)\right)}^{2}}{[a(\tilde{\phi }){]}^{2}}-\frac{b^{''}\left({\tilde{\eta }}_{i}\right)}{a(\tilde{\phi })}\right]x_{i}^{T}\sum_{g\in 𝒢}\;\;gh\left(g,D_{i}\right)=-\sum_{i=1}^{N}\;\;{\left\{\sum_{g\in 𝒢}\;\;h\left(g,D_{i}\right)\right\}}^{-1}\frac{b^{''}\left({\tilde{\eta }}_{i}\right)}{a(\tilde{\phi })}x_{i}^{T}\sum_{g\in 𝒢}\;\;gh\left(g,D_{i}\right)=-\sum_{i=1}^{N}\;\;\frac{b^{''}\left({\tilde{\eta }}_{i}\right)}{a(\tilde{\phi })}x_{i}^{T}{\left\{\sum_{g\in 𝒢}\;\;h\left(g,D_{i}\right)\right\}}^{-1}\sum_{g\in 𝒢}\;\;gh\left(g,D_{i}\right)=-\frac{1}{a(\tilde{\phi })}\sum_{i=1}^{N}\;\;b^{''}\left(\tilde{\alpha }x_{i}^{T}\right)x_{i}^{T}E\left(g|D_{i}\right)$$

$$\begin{array}{ll} & {\left.\frac{\partial^{2}l_{y,D}(\alpha ,\beta ,\phi )}{\partial \alpha^{T}\partial \phi }\right|}_{\tilde{\theta }}\\ = & \;\sum_{i=1}^{N}\;\;\left(-{\left\{p_{\theta }\left(y_{i},D_{i}|x_{i}\right)\right\}}^{-2}\right)\left[\sum_{g\in 𝒢}\;\;f_{\theta }\left(y_{i}|x_{i},g\right)\frac{y_{i}-b^{'}\left({\tilde{\eta }}_{i}\right)}{a(\tilde{\phi })}x_{i}^{T}h\left(g,D_{i}\right)\right]\left[\sum_{g\in 𝒢}\;\;f_{\theta }\left(y_{i}|x_{i},g\right)\left(-\frac{y_{i}{\tilde{\eta }}_{i}-b\left({\tilde{\eta }}_{i}\right)}{[a(\tilde{\phi }){]}^{2}}a^{'}(\tilde{\phi })+{\left.\frac{\partial c\left(y_{i},\phi \right)}{\partial \phi }\right|}_{\tilde{\theta }}\right)h\left(g,D_{i}\right)\right]\\ & \;+{\left\{p_{\theta }\left(y_{i},D_{i}|x_{i}\right)\right\}}^{-1}\sum_{g\in 𝒢}\;\;f_{\theta }\left(y_{i}|x_{i},g\right)\left[\left(-\frac{y_{i}{\tilde{\eta }}_{i}-b\left({\tilde{\eta }}_{i}\right)}{[a(\tilde{\phi }){]}^{2}}a^{'}(\tilde{\phi })+{\left.\frac{\partial c\left(y_{i},\phi \right)}{\partial \phi }\right|}_{\tilde{\theta }}\right)\left(\frac{y_{i}-b^{'}\left({\tilde{\eta }}_{i}\right)}{a(\tilde{\phi })}\right)-\frac{y_{i}-b^{'}\left({\tilde{\eta }}_{i}\right)}{[a(\tilde{\phi }){]}^{2}}a^{'}(\tilde{\phi })\right]x_{i}^{T}h\left(g,D_{i}\right)\\ = & \;\sum_{i=1}^{N}\;\;\left(-{\left\{f_{\theta }\left(y_{i}|x_{i}\right)\sum_{g\in 𝒢}\;\;h\left(g,D_{i}\right)\right\}}^{-2}\right)\left[f_{\theta }\left(y_{i}|x_{i}\right)\frac{y_{i}-b^{'}\left({\tilde{\eta }}_{i}\right)}{a(\tilde{\phi })}x_{i}^{T}\sum_{g\in 𝒢}\;\;h\left(g,D_{i}\right)\right]\left[f_{\theta }\left(y_{i}|x_{i}\right)\left(-\frac{y_{i}{\tilde{\eta }}_{i}-b\left({\tilde{\eta }}_{i}\right)}{[a(\tilde{\phi }){]}^{2}}+{\left.\frac{\partial c\left(y_{i},\phi \right)}{\partial \phi }\right|}_{\tilde{\theta }}\right)\sum_{g\in 𝒢}\;\;h\left(g,D_{i}\right)\right]\\ & \;+{\left\{f_{\theta }\left(y_{i}|x_{i}\right)\sum_{g\in 𝒢}\;\;h\left(g,D_{i}\right)\right\}}^{-1}f_{\theta }\left(y_{i}|x_{i}\right)\left[\left(-\frac{y_{i}{\tilde{\eta }}_{i}-b\left({\tilde{\eta }}_{i}\right)}{[a(\tilde{\phi }){]}^{2}}a^{'}(\tilde{\phi })+{\left.\frac{\partial c\left(y_{i},\phi \right)}{\partial \phi }\right|}_{\tilde{\theta }}\right)\left(\frac{y_{i}-b^{'}\left({\tilde{\eta }}_{i}\right)}{a(\tilde{\phi })}\right)-\frac{y_{i}-b^{'}\left({\tilde{\eta }}_{i}\right)}{[a(\tilde{\phi }){]}^{2}}a^{'}(\tilde{\phi })\right]x_{i}^{T}\sum_{g\in 𝒢}\;\;h\left(g,D_{i}\right)\\ = & \;\sum_{i=1}^{N}\;\;(-1)\left[\frac{y_{i}-b^{'}\left({\tilde{\eta }}_{i}\right)}{a(\tilde{\phi })}x_{i}^{T}\right]\left[-\frac{y_{i}{\tilde{\eta }}_{i}-b\left({\tilde{\eta }}_{i}\right)}{[a(\tilde{\phi }){]}^{2}}a^{'}(\tilde{\phi })+{\left.\frac{\partial c\left(y_{i},\phi \right)}{\partial \phi }\right|}_{\tilde{\theta }}\right]+\left[\left(-\frac{y_{i}{\tilde{\eta }}_{i}-b\left({\tilde{\eta }}_{i}\right)}{[a(\tilde{\phi }){]}^{2}}a^{'}(\tilde{\phi })+{\left.\frac{\partial c\left(y_{i},\phi \right)}{\partial \phi }\right|}_{\tilde{\theta }}\right)\left(\frac{y_{i}-b^{'}\left({\tilde{\eta }}_{i}\right)}{a(\tilde{\phi })}\right)-\frac{y_{i}-b^{'}\left({\tilde{\eta }}_{i}\right)}{[a(\tilde{\phi }){]}^{2}}a^{'}(\tilde{\phi })\right]x\\ = & \;\sum_{i=1}^{N}\;\;\left[-\frac{y_{i}-b^{'}\left({\tilde{\eta }}_{i}\right)}{[a(\tilde{\phi }){]}^{2}}a^{'}(\tilde{\phi })x_{i}^{T}\right]=-\frac{a^{'}(\tilde{\phi })}{[a(\tilde{\phi }){]}^{2}}\sum_{i=1}^{N}\;\;\left[\left(y_{i}-b^{'}\left({\tilde{\eta }}_{i}\right)\right)x_{i}^{T}\right]\\ = & 0.\text{ By Constrained MLE First Order Condition } \end{array}$$

$$\begin{array}{ll} & {\left.\frac{\partial^{2}l_{y,D}(\alpha ,\beta ,\phi )}{\partial \beta^{T}\partial \beta }\right|}_{\tilde{\theta }}\\ & \;=\sum_{i=1}^{N}\;\;\left(-{\left\{p_{\theta }\left(y_{i},D_{i}|x_{i}\right)\right\}}^{-2}\right)\left[\sum_{g\in 𝒢}\;\;f_{\theta }\left(y_{i}|x_{i},g\right)\frac{y_{i}-b^{'}\left({\tilde{\eta }}_{i}\right)}{a(\tilde{\phi })}g^{T}h\left(g,D_{i}\right)\right]\left[\sum_{g\in 𝒢}\;\;f_{\theta }\left(y_{i}|x_{i},g\right)\frac{y_{i}-b^{'}\left({\tilde{\eta }}_{i}\right)}{a(\tilde{\phi })}gh\left(g,D_{i}\right)\right]\\ & \;+{\left\{p_{\theta }\left(y_{i},D_{i}|x_{i}\right)\right\}}^{-1}\sum_{g\in 𝒢}\;\;f_{\theta }\left(y_{i}|x_{i},g\right)\left[\frac{{\left(y_{i}-b^{'}\left({\tilde{\eta }}_{i}\right)\right)}^{2}}{[a(\tilde{\phi }){]}^{2}}-\frac{b^{''}\left({\tilde{\eta }}_{i}\right)}{a(\tilde{\phi })}\right]g^{T}gh\left(g,D_{i}\right)\\ & \;=\sum_{i=1}^{N}\;\;\left(-{\left\{f_{\theta }\left(y_{i}|x_{i}\right)\sum_{g\in 𝒢}\;\;h\left(g,D_{i}\right)\right\}}^{-2}\right)\left[\sum_{g\in 𝒢}\;\;f_{\theta }\left(y_{i}|x_{i},g\right)\frac{y_{i}-b^{'}\left({\tilde{\eta }}_{i}\right)}{a(\tilde{\phi })}g^{T}h\left(g,D_{i}\right)\right]\left[\sum_{g\in 𝒢}\;\;f_{\theta }\left(y_{i}|x_{i},g\right)\frac{y_{i}-b^{'}\left({\tilde{\eta }}_{i}\right)}{a(\tilde{\phi })}gh\left(g,D_{i}\right)\right]\\ & \;+{\left\{f_{\theta }\left(y_{i}|x_{i}\right)\sum_{g\in 𝒢}\;\;h\left(g,D_{i}\right)\right\}}^{-1}\sum_{g\in 𝒢}\;\;f_{\theta }\left(y_{i}|x_{i},g\right)\left[\frac{{\left(y_{i}-b^{'}\left({\tilde{\eta }}_{i}\right)\right)}^{2}}{[a(\tilde{\phi }){]}^{2}}-\frac{b^{''}\left({\tilde{\eta }}_{i}\right)}{a(\tilde{\phi })}\right]g^{T}gh\left(g,D_{i}\right)\\ & \;=\sum_{i=1}^{N}\;\;\left(-{\left\{f_{\theta }\left(y_{i}|x_{i}\right)\sum_{g\in 𝒢}\;\;h\left(g,D_{i}\right)\right\}}^{-2}\right)\left[f_{\theta }\left(y_{i}|x_{i}\right)\frac{y_{i}-b^{'}\left({\tilde{\eta }}_{i}\right)}{a(\tilde{\phi })}\sum_{g\in 𝒢}\;\;g^{T}h\left(g,D_{i}\right)\right]\left[f_{\theta }\left(y_{i}|x_{i}\right)\frac{y_{i}-b^{'}\left({\tilde{\eta }}_{i}\right)}{a(\tilde{\phi })}\sum_{g\in 𝒢}\;\;gh\left(g,D_{i}\right)\right]\\ & \;+{\left\{f_{\theta }\left(y_{i}|x_{i}\right)\sum_{g\in 𝒢}\;\;h\left(g,D_{i}\right)\right\}}^{-1}f_{\theta }\left(y_{i}|x_{i}\right)\left[\frac{{\left(y_{i}-b^{'}\left({\tilde{\eta }}_{i}\right)\right)}^{2}}{[a(\tilde{\phi }){]}^{2}}-\frac{b^{''}\left({\tilde{\eta }}_{i}\right)}{a(\tilde{\phi })}\right]\sum_{g\in 𝒢}\;\;g^{T}gh\left(g,D_{i}\right)\\ & \;=\sum_{i=1}^{N}\;\;\left(-{\left\{\sum_{g\in 𝒢}\;\;h\left(g,D_{i}\right)\right\}}^{-2}\right)\left[\frac{y_{i}-b^{'}\left({\tilde{\eta }}_{i}\right)}{a(\tilde{\phi })}\sum_{g\in 𝒢}\;\;g^{T}h\left(g,D_{i}\right)\right]\left[\frac{y_{i}-b^{'}\left({\tilde{\eta }}_{i}\right)}{a(\tilde{\phi })}\sum_{g\in 𝒢}\;\;gh\left(g,D_{i}\right)\right]\\ & \;+{\left\{\sum_{g\in 𝒢}\;\;h\left(g,D_{i}\right)\right\}}^{-1}\left[\frac{{\left(y_{i}-b^{'}\left({\tilde{\eta }}_{i}\right)\right)}^{2}}{[a(\tilde{\phi }){]}^{2}}-\frac{b^{''}\left({\tilde{\eta }}_{i}\right)}{a(\tilde{\phi })}\right]\sum_{g\in 𝒢}\;\;g^{T}gh\left(g,D_{i}\right)\\ & \;=\sum_{i=1}^{N}\;\;(-1)\left[\frac{y_{i}-b^{'}\left({\tilde{\eta }}_{i}\right)}{a(\tilde{\phi })}{\left\{\sum_{g\in 𝒢}\;\;h\left(g,D_{i}\right)\right\}}^{-1}\sum_{g\in 𝒢}\;\;g^{T}h\left(g,D_{i}\right)\right]\left[\frac{y_{i}-b^{'}\left({\tilde{\eta }}_{i}\right)}{a(\tilde{\phi })}{\left\{\sum_{g\in 𝒢}\;\;h\left(g,D_{i}\right)\right\}}^{-1}\sum_{g\in 𝒢}\;\;gh\left(g,D_{i}\right)\right]\\ & \;+\left[\frac{{\left(y_{i}-b^{'}\left({\tilde{\eta }}_{i}\right)\right)}^{2}}{[a(\tilde{\phi }){]}^{2}}-\frac{b^{''}\left({\tilde{\eta }}_{i}\right)}{a(\tilde{\phi })}\right]{\left\{\sum_{g\in 𝒢}\;\;h\left(g,D_{i}\right)\right\}}^{-1}\sum_{g\in 𝒢}\;\;g^{T}gh\left(g,D_{i}\right)\\ & \;=\sum_{i=1}^{N}\;\;\left[\frac{{\left(y_{i}-b^{'}\left(\tilde{\alpha }x_{i}^{T}\right)\right)}^{2}}{[a(\tilde{\phi }){]}^{2}}\left\{E\left(g^{T}g|D_{i}\right)-E\left(g^{T}|D_{i}\right)E\left(g|D_{i}\right)\right\}-\frac{b^{''}\left(\tilde{\alpha }x_{i}^{T}\right)}{a(\tilde{\phi })}E\left(g^{T}g|D_{i}\right)\right] \end{array}$$

$$\begin{array}{ll} & {\left.\frac{\partial^{2}l_{y,D}(\alpha ,\beta ,\phi )}{\partial \beta^{T}\partial \phi }\right|}_{\tilde{\theta }}\\ & \;=\sum_{i=1}^{N}\;\;\left(-{\left\{p_{\theta }\left(y_{i},D_{i}|x_{i}\right)\right\}}^{-2}\right)\left[\sum_{g\in 𝒢}\;\;f_{\theta }\left(y_{i}|x_{i},g\right)\frac{y_{i}-b^{'}\left({\tilde{\eta }}_{i}\right)}{a(\tilde{\phi })}g^{T}h\left(g,D_{i}\right)\right]\left\{\sum_{g\in 𝒢}\;\;f_{\theta }\left(y_{i}|x_{i},g\right)\left[-\frac{y_{i}{\tilde{\eta }}_{i}-b\left({\tilde{\eta }}_{i}\right)}{[a(\tilde{\phi }){]}^{2}}a^{'}(\tilde{\phi })+\frac{\partial c\left(y_{i},\phi \right)}{\partial \phi }|\tilde{\theta }\right]h\left(g,D_{i}\right)\right\}\\ & \;+{\left\{p_{\theta }\left(y_{i},D_{i}|x_{i}\right)\right\}}^{-1}\sum_{g\in 𝒢}\;\;f_{\theta }\left(y_{i}|x_{i},g\right)\left(\left(\frac{y_{i}-b^{'}\left({\tilde{\eta }}_{i}\right)}{a(\tilde{\phi })}g^{T}\right)\left(-\frac{y_{i}{\tilde{\eta }}_{i}-b\left({\tilde{\eta }}_{i}\right)}{[a(\tilde{\phi }){]}^{2}}a^{'}(\tilde{\phi })+{\left.\frac{\partial c\left(y_{i},\phi \right)}{\partial \phi }\right|}_{\tilde{\theta }}\right)-\left(\frac{y_{i}-b^{'}\left({\tilde{\eta }}_{i}\right)}{[a(\tilde{\phi }){]}^{2}}a^{'}(\tilde{\phi })g^{T}\right)\right)h\left(g,D_{i}\right)\\ & \;=\sum_{i=1}^{N}\;\;\left(-{\left\{f_{\theta }\left(y_{i}|x_{i}\right)\sum_{g\in 𝒢}\;\;h\left(g,D_{i}\right)\right\}}^{-2}\right)\left[f_{\theta }\left(y_{i}|x_{i}\right)\frac{y_{i}-b^{'}\left({\tilde{\eta }}_{i}\right)}{a(\tilde{\phi })}\sum_{g\in 𝒢}\;\;g^{T}h\left(g,D_{i}\right)\right]\left\{f_{\theta }\left(y_{i}|x_{i}\right)\left[-\frac{y_{i}{\tilde{\eta }}_{i}-b\left({\tilde{\eta }}_{i}\right)}{[a(\tilde{\phi }){]}^{2}}a^{'}(\tilde{\phi })+{\left.\frac{\partial c\left(y_{i},\phi \right)}{\partial \phi }\right|}_{\tilde{\theta }}\right]\sum_{g\in 𝒢}\;\;h\left(g,D_{i}\right)\right\}\\ & \;+{\left\{f_{\theta }\left(y_{i}|x_{i}\right)\sum_{g\in 𝒢}\;\;h\left(g,D_{i}\right)\right\}}^{-1}f_{\theta }\left(y_{i}|x_{i}\right)\sum_{g\in 𝒢}\;\;\left(\left(\frac{y_{i}-b^{'}\left({\tilde{\eta }}_{i}\right)}{a(\tilde{\phi })}g^{T}\right)\left(-\frac{y_{i}{\tilde{\eta }}_{i}-b\left({\tilde{\eta }}_{i}\right)}{[a(\tilde{\phi }){]}^{2}}a^{'}(\tilde{\phi })+{\left.\frac{\partial c\left(y_{i},\phi \right)}{\partial \phi }\right|}_{\tilde{\theta }}\right)-\left(\frac{y_{i}-b^{'}\left({\tilde{\eta }}_{i}\right)}{[a(\tilde{\phi }){]}^{2}}a^{'}(\tilde{\phi })g^{T}\right)\right)h\left(g,D_{i}\right)\\ & \;=\sum_{i=1}^{N}\;\;\left(-{\left\{\sum_{g\in 𝒢}\;\;h\left(g,D_{i}\right)\right\}}^{-1}\right)\left[\frac{y_{i}-b^{'}\left({\tilde{\eta }}_{i}\right)}{a(\tilde{\phi })}\sum_{g\in 𝒢}\;\;g^{T}h\left(g,D_{i}\right)\right]\left\{\left[-\frac{y_{i}{\tilde{\eta }}_{i}-b\left({\tilde{\eta }}_{i}\right)}{[a(\tilde{\phi }){]}^{2}}a^{'}(\tilde{\phi })+{\left.\frac{\partial c\left(y_{i},\phi \right)}{\partial \phi }\right|}_{\tilde{\theta }}\right]\right\}\\ & \;+{\left\{\sum_{g\in 𝒢}\;\;h\left(g,D_{i}\right)\right\}}^{-1}\sum_{g\in 𝒢}\;\;\left(\left(\frac{y_{i}-b^{'}\left({\tilde{\eta }}_{i}\right)}{a(\tilde{\phi })}g^{T}\right)\left(-\frac{y_{i}{\tilde{\eta }}_{i}-b\left({\tilde{\eta }}_{i}\right)}{[a(\tilde{\phi }){]}^{2}}a^{'}(\tilde{\phi })+{\left.\frac{\partial c\left(y_{i},\phi \right)}{\partial \phi }\right|}_{\tilde{\theta }}\right)-\left(\frac{y_{i}-b^{'}\left({\tilde{\eta }}_{i}\right)}{[a(\tilde{\phi }){]}^{2}}a^{'}(\tilde{\phi })g^{T}\right)\right)h\left(g,D_{i}\right)\\ & \;=\sum_{i=1}^{N}\;\;\left(-{\left\{\sum_{g\in 𝒢}\;\;h\left(g,D_{i}\right)\right\}}^{-1}\right)\left[\frac{y_{i}-b^{'}\left({\tilde{\eta }}_{i}\right)}{a(\tilde{\phi })}\sum_{g\in 𝒢}\;\;g^{T}h\left(g,D_{i}\right)\right]\left\{\left[-\frac{y_{i}{\tilde{\eta }}_{i}-b\left({\tilde{\eta }}_{i}\right)}{[a(\tilde{\phi }){]}^{2}}a^{'}(\tilde{\phi })+{\left.\frac{\partial c\left(y_{i},\phi \right)}{\partial \phi }\right|}_{\tilde{\theta }}\right]\right\}\\ & \;+{\left\{\sum_{g\in 𝒢}\;\;h\left(g,D_{i}\right)\right\}}^{-1}\sum_{g\in 𝒢}\;\;\left(\left(\frac{y_{i}-b^{'}\left({\tilde{\eta }}_{i}\right)}{a(\tilde{\phi })}g^{T}\right)\left(-\frac{y_{i}{\tilde{\eta }}_{i}-b\left({\tilde{\eta }}_{i}\right)}{[a(\tilde{\phi }){]}^{2}}a^{'}(\tilde{\phi })+{\left.\frac{\partial c\left(y_{i},\phi \right)}{\partial \phi }\right|}_{\tilde{\theta }}\right)h\left(g,D_{i}\right)-{\left\{\sum_{g\in 𝒢}\;\;h\left(g,D_{i}\right)\right\}}^{-1}\sum_{g\in 𝒢}\;\;\left(\frac{y_{i}-b^{'}\left({\tilde{\eta }}_{i}\right)}{[a(\tilde{\phi }){]}^{'}}a^{'}(\tilde{\phi })g^{T}\right)\right)h\\ & \left.=-\sum_{i=1}^{N}\;\;{\left\{\sum_{g\in 𝒢}\;\;h\left(g,D_{i}\right)\right\}}^{-1}\sum_{g\in 𝒢}\;\;\left(\frac{y_{i}-b^{'}\left({\tilde{\eta }}_{i}\right)}{[a(\tilde{\phi }){]}^{2}}a^{'}(\tilde{\phi })g^{T}\right)\right)h\left(g,D_{i}\right)\\ & \;=-\sum_{i=1}^{N}\;\;\frac{y_{i}-b^{'}\left({\tilde{\eta }}_{i}\right)}{[a(\tilde{\phi }){]}^{2}}a^{'}(\tilde{\phi })\left({\left\{\sum_{g\in 𝒢}\;\;h\left(g,D_{i}\right)\right\}}^{-1}\sum_{g\in 𝒢}\;\;g^{T}h\left(g,D_{i}\right)\right)\\ & \;=-\frac{a^{'}(\tilde{\phi })}{[a(\tilde{\phi }){]}^{2}}\sum_{i=1}^{N}\;\;\left(y_{i}-b^{'}\left(\tilde{\alpha }x_{i}^{T}\right)\right)E\left(g^{T}|D_{i}\right) \end{array}$$

$$\begin{array}{ll} & {\left.\frac{\partial^{2}l_{y,D}(\alpha ,\beta ,\phi )}{\partial \phi^{2}}\right|}_{\tilde{\theta }}\\ = & \;\sum_{i=1}^{N}\;\;\left[-{\left\{p_{\theta }\left(y_{i},D_{i}|x_{i}\right)\right\}}^{-2}{\left(\sum_{g\in 𝒢}\;\;f_{\theta }\left(y_{i}|x_{i},g\right)\left[-\frac{y_{i}{\tilde{\eta }}_{i}-b\left({\tilde{\eta }}_{i}\right)}{[a(\tilde{\phi }){]}^{2}}a^{'}(\tilde{\phi })+{\left.\frac{\partial c\left(y_{i},\phi \right)}{\partial \phi }\right|}_{\tilde{\theta }}\right]h\left(g_{,},D_{i}\right)\right)}^{2}\right.\\ & \;+{\left\{p_{\theta }\left(y_{i},D_{i}|x_{i}\right)\right\}}^{-1}\sum_{g\in 𝒢}\;\;f_{\theta }\left(y_{i}|x_{i},g\right)\left\{{\left[-\frac{y_{i}{\tilde{\eta }}_{i}-b\left({\tilde{\eta }}_{i}\right)}{[a(\tilde{\phi }){]}^{2}}a^{'}(\tilde{\phi })+{\left.\frac{\partial c\left(y_{i},\phi \right)}{\partial \phi }\right|}_{\tilde{\theta }}\right]}^{2}+\left(y_{i}{\tilde{\eta }}_{i}-b\left({\tilde{\eta }}_{i}\right)\right)\left(\frac{2{\left[a^{'}(\tilde{\phi })\right]}^{2}}{[a(\tilde{\phi }){]}^{3}}-\frac{a^{''}(\phi )}{[a(\tilde{\phi }){]}^{2}}\right)+{\left.\frac{\partial^{2}c\left(y_{i},\phi \right)}{\partial \phi^{2}}\right|}_{\tilde{\theta }}\right\}h(g,D_{i}\\ = & \;\sum_{i=1}^{N}\;\;\left[-{\left\{f_{\theta }\left(y_{i}|x_{i}\right)\sum_{g\in 𝒢}\;\;h\left(g,D_{i}\right)\right\}}^{-2}{\left(f_{\theta }\left(y_{i}|x_{i}\right)\left[-\frac{y_{i}{\tilde{\eta }}_{i}-b\left({\tilde{\eta }}_{i}\right)}{[a(\tilde{\phi }){]}^{2}}a^{'}(\tilde{\phi })+{\left.\frac{\partial c\left(y_{i},\phi \right)}{\partial \phi }\right|}_{\tilde{\theta }}\right]\sum_{g\in 𝒢}\;\;h\left(g,D_{i}\right)\right)}^{2}\right.\\ & \;+{\left\{f_{\theta }\left(y_{i}|x_{i}\right)\sum_{g\in 𝒢}\;\;h\left(g^{'},D_{i}\right)\right\}}^{-1}f_{\theta }\left(y_{i}|x_{i}\right)\\ & \left.\left\{{\left[-\frac{y_{i}{\tilde{\eta }}_{i}-b\left({\tilde{\eta }}_{i}\right)}{[a(\tilde{\phi }){]}^{2}}a^{'}(\tilde{\phi })+{\left.\frac{\partial c\left(y_{i},\phi \right)}{\partial \phi }\right|}_{\tilde{\theta }}\right]}^{2}+\left(y_{i}{\tilde{\eta }}_{i}-b\left({\tilde{\eta }}_{i}\right)\right)\left(\frac{2{\left[a^{'}(\tilde{\phi })\right]}^{2}}{[a(\tilde{\phi }){]}^{3}}-\frac{a^{''}(\phi )}{[a(\tilde{\phi }){]}^{2}}\right)+{\left.\frac{\partial^{2}c\left(y_{i},\phi \right)}{\partial \phi^{2}}\right|}_{\tilde{\theta }}\right\}\sum_{g\in 𝒢}\;\;h\left(g,D_{i}\right)\right]\\ = & \;\sum_{i=1}^{N}\;\;\left[-{\left(\left[-\frac{y_{i}{\tilde{\eta }}_{i}-b\left({\tilde{\eta }}_{i}\right)}{[a(\tilde{\phi }){]}^{2}}a^{'}(\tilde{\phi })+{\left.\frac{\partial c\left(y_{i},\phi \right)}{\partial \phi }\right|}_{\tilde{\theta }}\right]\right)}^{2}\right.\\ & \left.+\left\{{\left[-\frac{y_{i}{\tilde{\eta }}_{i}-b\left({\tilde{\eta }}_{i}\right)}{[a(\tilde{\phi }){]}^{2}}a^{'}(\tilde{\phi })+{\left.\frac{\partial c\left(y_{i},\phi \right)}{\partial \phi }\right|}_{\tilde{\theta }}\right]}^{2}+\left(y_{i}{\tilde{\eta }}_{i}-b\left({\tilde{\eta }}_{i}\right)\right)\left(\frac{2{\left[a^{'}(\tilde{\phi })\right]}^{2}}{[a(\tilde{\phi }){]}^{3}}-\frac{a^{''}(\phi )}{[a(\tilde{\phi }){]}^{2}}\right)+{\left.\frac{\partial^{2}c\left(y_{i},\phi \right)}{\partial \phi^{2}}\right|}_{\tilde{\theta }}\right\}\right]\\ = & \;\sum_{i=1}^{N}\;\;\left[\left(y_{i}{\tilde{\eta }}_{i}-b\left({\tilde{\eta }}_{i}\right)\right)\left(\frac{2{\left[a^{'}(\tilde{\phi })\right]}^{2}}{[a(\tilde{\phi }){]}^{3}}-\frac{a^{''}(\phi )}{[a(\tilde{\phi }){]}^{2}}\right)+{\left.\frac{\partial^{2}c\left(y_{i},\phi \right)}{\partial \phi^{2}}\right|}_{\tilde{\theta }}\right]\\ = & \;\sum_{i=1}^{N}\;\;\left[\left(y_{i}\tilde{\alpha }x_{i}^{T}-b\left(\tilde{\alpha }x_{i}^{T}\right)\right)\left(\frac{2{\left[a^{'}(\tilde{\phi })\right]}^{2}}{[a(\tilde{\phi }){]}^{3}}-\frac{a^{''}(\phi )}{[a(\tilde{\phi }){]}^{2}}\right)+{\left.\frac{\partial^{2}c\left(y_{i},\phi \right)}{\partial \phi^{2}}\right|}_{\tilde{\theta }}\right] \end{array}$$

In summary, we have

$$\begin{array}{ll} & {\left.\frac{\partial^{2}l_{y,D}(\alpha ,\beta ,\phi )}{\partial \alpha^{T}\partial \alpha }\right|}_{\tilde{\theta }}=-\frac{1}{a(\tilde{\phi })}\sum_{i=1}^{N}\;\;b^{''}\left(\tilde{\alpha }x_{i}^{T}\right)x_{i}^{T}x_{i}\\ & {\left.\frac{\partial^{2}l_{y,D}(\alpha ,\beta ,\phi )}{\partial \alpha^{T}\partial \beta }\right|}_{\tilde{\theta }}=-\frac{1}{a(\tilde{\phi })}\sum_{i=1}^{N}\;\;b^{''}\left(\tilde{\alpha }x_{i}^{T}\right)x_{i}^{T}E\left(g|D_{i}\right)\\ & {\left.\frac{\partial^{2}l_{y,D}(\alpha ,\beta ,\phi )}{\partial \alpha^{T}\partial \phi }\right|}_{\tilde{\theta }}=-\frac{a^{'}(\tilde{\phi })}{[a(\tilde{\phi }){]}^{2}}\sum_{i=1}^{N}\;\;\left[\left(y_{i}-b^{'}\left(\tilde{\alpha }x^{T}\right)\right)x_{i}^{T}\right]=0\\ & {\left.\frac{\partial^{2}l_{y,D}(\alpha ,\beta ,\phi )}{\partial \beta^{T}\partial \beta }\right|}_{\tilde{\theta }}=\sum_{i=1}^{N}\;\;\left[\frac{{\left(y_{i}-b^{'}\left(\tilde{\alpha }x_{i}^{T}\right)\right)}^{2}}{[a(\tilde{\phi }){]}^{2}}\left(E\left(g^{T}g|D_{i}\right)-E\left(g^{T}|D_{i}\right)E\left(g|D_{i}\right)\right)-\frac{b^{''}\left(\tilde{\alpha }x_{i}^{T}\right)}{a(\tilde{\phi })}E\left(g^{T}g|D_{i}\right)\right]\text{. }\\ & {\left.\frac{\partial^{2}l_{y,D}(\alpha ,\beta ,\phi )}{\partial \beta^{T}\partial \phi }\right|}_{\tilde{\theta }}=-\frac{a^{'}(\tilde{\phi })}{[a(\tilde{\phi }){]}^{2}}\sum_{i=1}^{N}\;\;\left(y_{i}-b^{'}\left(\tilde{\alpha }x_{i}^{T}\right)\right)E\left(g^{T}|D_{i}\right)\\ & {\left.\frac{\partial^{2}l_{y,D}(\alpha ,\beta ,\phi )}{\partial \phi^{2}}\right|}_{\tilde{\theta }}=\sum_{i=1}^{N}\;\;\left[\left(y_{i}\tilde{\alpha }x_{i}^{T}-b\left(\tilde{\alpha }x_{i}^{T}\right)\right)\left(\frac{2{\left[a^{'}(\tilde{\phi })\right]}^{2}}{[a(\tilde{\phi }){]}^{3}}-\frac{a^{''}(\phi )}{[a(\tilde{\phi }){]}^{2}}\right)+{\left.\frac{\partial^{2}c\left(y_{i},\phi \right)}{\partial \phi^{2}}\right|}_{\tilde{\theta }}\right] \end{array}$$

In addition, we can obtain the following terms by matrix transposition.

$${\left.\frac{\partial^{2}l_{y,D}(\alpha ,\beta ,\phi )}{\partial \beta^{T}\partial \alpha }\right|}_{\tilde{\theta }}={\left({\left.\frac{\partial^{2}l_{y,D}(\alpha ,\beta ,\phi )}{\partial \alpha^{T}\partial \beta }\right|}_{\tilde{\theta }}\right)}^{T};{\left.\;\frac{\partial^{2}l_{y,D}(\alpha ,\beta ,\phi )}{\partial \phi \partial \alpha }\right|}_{\tilde{\theta }}={\left({\left.\frac{\partial^{2}l_{y,D}(\alpha ,\beta ,\phi )}{\partial \alpha^{T}\partial \phi }\right|}_{\tilde{\theta }}\right)}^{T};{\left.\;\frac{\partial^{2}l_{y,D}(\alpha ,\beta ,\phi )}{\partial \phi \partial \beta }\right|}_{\tilde{\theta }}={\left({\left.\frac{\partial^{2}l_{y,D}(\alpha ,\beta ,\phi )}{\partial \beta^{T}\partial \phi }\right|}_{\tilde{\theta }}\right)}^{T}$$

## Appendix F Analytical Formula of Score Function when Parameters are (α,β)

The score function is

$$s_{y,D}(\alpha ,\beta )=\left[\begin{array}{l} \frac{\partial l_{y,D}(\alpha ,\beta )}{\partial \alpha^{T}}\\ \frac{\partial l_{y,D}(\alpha ,\beta )}{\partial \beta^{T}} \end{array}\right]$$

We derived each element in the above vector as the following.

$$\begin{array}{ll} \frac{\partial l_{y,D}(\alpha ,\beta ,\phi )}{\partial \alpha^{T}} & \;=\frac{\partial \sum_{i=1}^{N}\;\;ln\left(p_{\theta }\left(y_{i},D_{i}|x_{i}\right)\right)}{\partial \alpha^{T}}=\sum_{i=1}^{N}\;\;\frac{\partial ln\left(p_{\theta }\left(y_{i},D_{i}|x_{i}\right)\right)}{\partial \alpha^{T}}\\ & \;=\sum_{i=1}^{N}\;\;\left[{\left\{p_{\theta }\left(y_{i},D_{i}|x_{i}\right)\right\}}^{-1}\frac{\partial p_{\theta }\left(y_{i},D_{i}|x_{i}\right)}{\partial \alpha^{T}}\right]\\ & \;=\sum_{i=1}^{N}\;\;\left[{\left\{p_{\theta }\left(y_{i},D_{i}|x_{i}\right)\right\}}^{-1}\frac{\partial \sum_{g\in 𝒢}\;\;f_{\theta }\left(y_{i}|x_{i},g\right)h\left(g,D_{i}\right)}{\partial \alpha^{T}}\right]\\ & \;=\sum_{i=1}^{N}\;\;\left[{\left\{p_{\theta }\left(y_{i},D_{i}|x_{i}\right)\right\}}^{-1}\sum_{g\in 𝒢}\;\;\frac{\partial \left\{exp\left(\frac{y_{i}\eta_{i}-b\left(\eta_{i}\right)}{a}+c\left(y_{i}\right)\right)\right\}}{\partial \alpha^{T}}h\left(g,D_{i}\right)\right]\\ & \;=\sum_{i=1}^{N}\;\;\left[{\left\{p_{\theta }\left(y_{i},D_{i}|x_{i}\right)\right\}}^{-1}\sum_{g\in 𝒢}\;\;exp\left\{\frac{y_{i}\eta_{i}-b\left(\eta_{i}\right)}{a}+c\left(y_{i}\right)\right\}\frac{y_{i}-b^{'}\left(\eta_{i}\right)}{a}x_{i}^{T}h\left(g,D_{i}\right)\right]\\ & \;=\sum_{i=1}^{N}\;\;\left[{\left\{p_{\theta }\left(y_{i},D_{i}|x_{i}\right)\right\}}^{-1}\sum_{g\in 𝒢}\;\;f_{\theta }\left(y_{i}|x_{i},g\right)\frac{y_{i}-b^{'}\left(\eta_{i}\right)}{a}x_{i}^{T}h\left(g,D_{i}\right)\right]. \end{array}$$

$$\begin{array}{ll} \frac{\partial l_{y,D}(\alpha ,\beta ,\phi )}{\partial \beta^{T}} & \;=\frac{\partial \sum_{i=1}^{N}\;\;log\left(p_{\theta }\left(y_{i},D_{i}|x_{i}\right)\right)}{\partial \beta^{T}}=\sum_{i=1}^{N}\;\;\frac{\partial log\left(p_{\theta }\left(y_{i},D_{i}|x_{i}\right)\right)}{\partial \beta^{T}}\\ & \;=\sum_{i=1}^{N}\;\;\left[{\left\{p_{\theta }\left(y_{i},D_{i}|x_{i}\right)\right\}}^{-1}\frac{\partial p_{\theta }\left(y_{i},D_{i}|x_{i}\right)}{\partial \beta^{T}}\right]\\ & \;=\sum_{i=1}^{N}\;\;\left[{\left\{p_{\theta }\left(y_{i},D_{i}|x_{i}\right)\right\}}^{-1}\frac{\partial \sum_{g\in 𝒢}\;\;f_{\theta }\left(y_{i}|x_{i},g\right)h\left(g,D_{i}\right)}{\partial \beta^{T}}\right]\\ & \;=\sum_{i=1}^{N}\;\;\left[{\left\{p_{\theta }\left(y_{i},D_{i}|x_{i}\right)\right\}}^{-1}\sum_{g\in 𝒢}\;\;\frac{\partial \left\{exp\left(\frac{y_{i}\eta_{i}-b\left(\eta_{i}\right)}{a}+c\left(y_{i}\right)\right)\right\}}{\partial \beta^{T}}h\left(g,D_{i}\right)\right]\\ & \;=\sum_{i=1}^{N}\;\;{\left\{p_{\theta }\left(y_{i},D_{i}|x_{i}\right)\right\}}^{-1}\sum_{g\in 𝒢}\;\;exp\left\{\frac{y_{i}\eta_{i}-b\left(\eta_{i}\right)}{a}+c\left(y_{i}\right)\right\}\frac{y_{i}-b^{'}\left(\eta_{i}\right)}{a}g^{T}h\left(g,D_{i}\right)\\ & \;=\sum_{i=1}^{N}\;\;{\left\{p_{\theta }\left(y_{i},D_{i}|x_{i}\right)\right\}}^{-1}\sum_{g\in 𝒢}\;\;f_{\theta }\left(y_{i}|x_{i},g\right)\frac{y_{i}-b^{'}\left(\eta_{i}\right)}{a}g^{T}h\left(g,D_{i}\right). \end{array}$$

So write compactly, we have

$$s_{y,D}\left(\alpha ,\beta \right)=\sum_{i=1}^{N}\;\left[{\left\{p_{\theta }\left(y_{i},D_{i}|x_{i}\right)\right\}}^{-1}\sum_{g\in 𝒢}\;\;f_{\theta }\left(y_{i}|x_{i},g\right)\left[\begin{array}{l} \frac{y_{i}-b^{'}\left(\eta_{i}\right)}{a}x_{i}^{T}\\ \frac{y_{i}-b^{'}\left(\eta_{i}\right)}{a}g^{T} \end{array}\right]h\left(g,D_{i}\right)\right],$$

where $\eta_{i}=\eta \left(x_{i},g\right)=\alpha x_{i}^{T}+\beta g^{T}$.

## Appendix G Analytical Formula of Observed Information Matrix when Parameters are (α,β)

The observed information is

$$o_{y,D}\left(\alpha ,\beta \right)=-\left[\begin{array}{ll} \frac{\partial^{2}l_{y,D}\left(\alpha ,\beta \right)}{\partial \alpha^{T}\partial \alpha } & \frac{\partial^{2}l_{y,D}\left(\alpha ,\beta \right)}{\partial \alpha^{T}\partial \beta }\\ \frac{\partial^{2}l_{y,D}\left(\alpha ,\beta \right)}{\partial \beta^{T}\partial \alpha } & \frac{\partial^{2}l_{y,D}\left(\alpha ,\beta \right)}{\partial \beta^{T}\partial \beta } \end{array}\right].$$

We derive each element in the matrix as the following.

We start from the score function, which is derived as

$$s_{y,D}\left(\alpha ,\beta \right)=\sum_{i=1}^{N}\;\left[{\left\{p_{\theta }\left(y_{i},D_{i}|x_{i}\right)\right\}}^{-1}\sum_{g\in 𝒢}\;\;f_{\theta }\left(y_{i}|x_{i},g\right)\left[\begin{array}{l} \frac{y_{i}-b^{'}\left(\eta_{i}\right)}{a}x_{i}^{T}\\ \frac{y_{i}-b^{'}\left(\eta_{i}\right)}{a}g^{T} \end{array}\right]h\left(g,D_{i}\right)\right],$$

where $\eta_{i}=\eta \left(x_{i},g\right)=\alpha x_{i}^{T}+\beta g^{T}$. Take the derivative of the score function.

$$\frac{\partial^{2}l_{y,D}(\alpha ,\beta )}{\partial \alpha^{⊤}\partial \alpha }=\frac{\frac{\partial l_{y,D}(\alpha ,\beta )}{\partial \alpha^{⊤}}}{\partial \alpha }\;\;=\frac{\partial \left[\sum_{i=1}^{N}\;\;{\left\{p_{\theta }\left(y_{i},D_{i}|x_{i}\right)\right\}}^{-1}\sum_{g\in 𝒢}\;\;f_{\theta }{\left(y_{i}|x_{i},g\right)}^{\frac{y_{i}-b^{'}\left(\eta_{i}i\right.}{a}}x_{i}^{T}h\left(g,D_{i}\right)\right]}{\partial \alpha }\;\;=\sum_{i=1}^{N}\;\;\frac{\partial \left[{\left\{p_{\theta }\left(y_{i},D_{i}|x_{i}\right)\right\}}^{-1}\sum_{g}\;\;\in 𝒢f_{\theta }\left(y_{i}|x_{i},g\right)\frac{y_{i}-b^{'}\left(\eta_{i}\right)}{a}x_{i}^{T}h\left(g,D_{i}\right)\right]}{\partial \alpha }\;\;=\sum_{i=1}^{N}\;\;\left[\sum_{g\in 𝒢}\;\;f_{\theta }\left(y_{i}|x_{i},g\right)\frac{y_{i}-b^{'}\left(\eta_{i}\right)}{a}x_{i}^{T}h\left(g,D_{i}\right)\right]\frac{\partial {\left\{p_{\theta }\left(y_{i},D_{i}|x_{i}\right)\right\}}^{-1}}{\partial \alpha }+{\left\{p_{\theta }\left(y_{i},D_{i}|x_{i}\right)\right\}}^{-1}\frac{\partial \sum_{g\in 𝒢}\;\;f_{\theta }{\left(y_{i}|x_{i},g\right)}^{\frac{y_{i}}{\;}b^{'}\left(\eta_{i}\right)}x_{i}^{T}h\left(g,D_{i}\right)}{\partial \alpha }\;\;=\sum_{i=1}^{N}\;\;\left[\sum_{g\in 𝒢}\;\;f_{\theta }\left(y_{i}|x_{i},g\right)\frac{y_{i}-b^{'}\left(\eta_{i}\right)}{a}x_{i}^{T}h\left(g,D_{i}\right)\right]\left[\left(-{\left\{p_{\theta }\left(y_{i},D_{i}|x_{i}\right)\right\}}^{-2}\right)\sum_{g\in 𝒢}\;\;f_{\theta }\left(y_{i}|x_{i},g\right)\frac{y_{i}-b^{'}\left(\eta_{i}\right)}{a}x_{i}h\left(g,D_{i}\right)\right]\;\;+{\left\{p_{\theta }\left(y_{i},D_{i}|x_{i}\right)\right\}}^{-1}\sum_{g\in 𝒢}\;\;\frac{\partial f_{\theta }\left(y_{i}|x_{i},g\right)\frac{y_{i}-b^{'}\left(\eta_{i}\right)}{a}x_{i}^{T}}{\partial \alpha }h\left(g,D_{i}\right)\;\;=\sum_{i=1}^{N}\;\;\left(-{\left\{p_{\theta }\left(y_{i},D_{i}|x_{i}\right)\right\}}^{-2}\right)\left[\sum_{g\in 𝒢}\;\;f_{\theta }\left(y_{i}|x_{i},g\right)\frac{y_{i}-b^{'}\left(\eta_{i}\right)}{a}x_{i}^{T}h\left(g,D_{i}\right)\right]\left[\sum_{g\in 𝒢}\;\;f_{\theta }\left(y_{i}|x_{i},g\right)\frac{y_{i}-b^{'}\left(\eta_{i}\right)}{a}x_{i}h\left(g,D_{i}\right)\right]\;\;+{\left\{p_{\theta }\left(y_{i},D_{i}|x_{i}\right)\right\}}^{-1}\sum_{g\in 𝒢}\;\;f_{\theta }\left(y_{i}|x_{i},g\right)\left[\left(\frac{y_{i}-b^{'}\left(\eta_{i}\right)}{a}x_{i}^{T}\right)\frac{\partial \left[\frac{y_{i}\eta_{i}-b\left(\eta_{i}\right)}{a}+c\left(y_{i}\right)\right]}{\partial \alpha }+\frac{\partial \left[\frac{y_{i}-b^{'}\left(\eta_{i}\right)}{\partial \alpha }x_{i}^{T}\right]}{\partial \alpha }\right]h\left(g,D_{i}\right)\;\;=\sum_{i=1}^{N}\;\;\left(-{\left\{p_{\theta }\left(y_{i},D_{i}|x_{i}\right)\right\}}^{-2}\right)\left[\sum_{g\in 𝒢}\;\;f_{\theta }\left(y_{i}|x_{i},g\right)\frac{y_{i}-b^{'}\left(\eta_{i}\right)}{a}x_{i}^{T}h\left(g,D_{i}\right)\right]\left[\sum_{g\in 𝒢}\;\;f_{\theta }\left(y_{i}|x_{i},g\right)\frac{y_{i}-b^{'}\left(\eta_{i}\right)}{a}x_{i}h\left(g,D_{i}\right)\right]\;\;+{\left\{p_{\theta }\left(y_{i},D_{i}|x_{i}\right)\right\}}^{-1}\sum_{g\in 𝒢}\;\;f_{\theta }\left(y_{i}|x_{i},g\right)\left[\left(\frac{y_{i}-b^{'}\left(\eta_{i}\right)}{a}x_{i}^{T}\right)\left(\frac{y_{i}-b^{'}\left(\eta_{i}\right)}{a}x_{i}\right)-\frac{b^{''}\left(\eta_{i}\right)}{a}x_{i}^{T}x_{i}\right]h\left(g,D_{i}\right)\;\;=\sum_{i=1}^{N}\;\;\left(-{\left\{p_{\theta }\left(y_{i},D_{i}|x_{i}\right)\right\}}^{-2}\right)\left[\sum_{g\in 𝒢}\;\;f_{\theta }\left(y_{i}|x_{i},g\right)\frac{y_{i}-b^{'}\left(\eta_{i}\right)}{a}x_{i}^{T}h\left(g,D_{i}\right)\right]\left[\sum_{\varepsilon \in 𝒢}\;\;f_{\theta }\left(y_{i}|x_{i},g\right)\frac{y_{i}-b^{'}\left(\eta_{i}\right)}{a}x_{i}h\left(g,D_{i}\right)\right]\;\;+{\left\{p_{\theta }\left(y_{i},D_{i}|x_{i}\right)\right\}}^{-1}\sum_{g\in 𝒢}\;\;f_{\theta }\left(y_{i}|x_{i},g\right)\left[\frac{{\left(y_{i}-b^{'}\left(\eta_{i}\right)\right)}^{2}}{a^{2}}-\frac{b^{''}\left(\eta_{i}\right)}{a}\right]x_{i}^{T}x_{i}h\left(g,D_{i}\right)$$

$$\frac{\partial^{2}l_{y,D}(\alpha ,\beta )}{\partial \alpha^{T}\partial \beta }=\frac{\partial \frac{\partial l_{y,D}(\alpha ,\beta )}{\partial \alpha^{T}}}{\partial \beta }\;\;=\frac{\partial \left[\sum_{i=1}^{N}\;\;{\left\{p_{\theta }\left(y_{i},D_{i}|x_{i}\right)\right\}}^{-1}\sum_{g\in 𝒢}\;\;f_{\theta }\left(y_{i}|x_{i},g\right)\frac{y_{i}-b^{'}\left(\eta_{i}\right)}{a}x_{i}^{T}h\left(g,D_{i}\right)\right]}{\partial \beta }\;\;=\sum_{i=1}^{N}\;\;\frac{\partial \left[{\left\{p_{\theta }\left(y_{i},D_{i}|x_{i}\right)\right\}}^{-1}\sum_{g\in 𝒢}\;\;f_{\theta }\left(y_{i}|x_{i},g\right)\frac{y_{i}-b^{'}\left(\eta_{i}\right)}{a}x_{i}^{T}h\left(g,D_{i}\right)\right]}{\partial \beta }\;\;=\sum_{i=1}^{N}\;\;\left[\sum_{g\in 𝒢}\;\;f_{\theta }\left(y_{i}|x_{i},g\right)\frac{y_{i}-b^{'}\left(\eta_{i}\right)}{a}x_{i}^{T}h\left(g,D_{i}\right)\right]\frac{\partial {\left\{p_{\theta }\left(y_{i},D_{i}|x_{i}\right)\right\}}^{-1}}{\partial \beta }+{\left\{p_{\theta }\left(y_{i},D_{i}|x_{i}\right)\right\}}^{-1}\frac{\partial \left[\sum_{g\in 𝒢}\;\;f_{\theta }\left(y_{i}|x_{i},g\right)\frac{y_{i}-b^{'}\left(\eta_{i}\right)}{a}x_{i}^{T}h\left(g,D_{i}\right)\right.}{\partial \beta }\;\;=\sum_{i=1}^{N}\;\;\left[\sum_{g\in 𝒢}\;\;f_{\theta }\left(y_{i}|x_{i},g\right)\frac{y_{i}-b^{'}\left(\eta_{i}\right)}{a}x_{i}^{T}h\left(g,D_{i}\right)\right]\left[\left(-{\left\{p_{\theta }\left(y_{i},D_{i}|x_{i}\right)\right\}}^{-2}\right)\sum_{g\in 𝒢}\;\;f_{\theta }\left(y_{i}|x_{i},g\right)\frac{y_{i}-b^{'}\left(\eta_{i}\right)}{a}gh\left(g,D_{i}\right)\right]\;\;+{\left\{p_{\theta }\left(y_{i},D_{i}|x_{i}\right)\right\}}^{-1}\sum_{g\in 𝒢}\;\;\frac{\partial \left[f_{\theta }\left(y_{i}|x_{i},g\right)\frac{y_{i}-b^{'}\left(\eta_{i}\right)}{a}x_{i}^{T}\right]}{\partial \beta }h\left(g,D_{i}\right)\;\;=\sum_{i=1}^{N}\;\;\left(-{\left\{p_{\theta }\left(y_{i},D_{i}|x_{i}\right)\right\}}^{-2}\right)\left[\sum_{g\in 𝒢}\;\;f_{\theta }\left(y_{i}|x_{i},g\right)\frac{y_{i}-b^{'}\left(\eta_{i}\right)}{a}x_{i}^{T}h\left(g,D_{i}\right)\right]\left[\sum_{g\in 𝒢}\;\;f_{\theta }\left(y_{i}|x_{i},g\right)\frac{y_{i}-b^{'}\left(\eta_{i}\right)}{a}gh\left(g,D_{i}\right)\right]\;\;+{\left\{p_{\theta }\left(y_{i},D_{i}|x_{i}\right)\right\}}^{-1}\sum_{g\in 𝒢}\;\;f_{\theta }\left(y_{i}|x_{i},g\right)\left[\left(\frac{y_{i}-b^{'}\left(\eta_{i}\right)}{a}x_{i}^{T}\right)\frac{\partial \left[\frac{y_{i}\eta_{i}-b\left(\eta_{i}\right)}{a}+c\left(y_{i}\right)\right]}{\partial \beta }+\frac{\partial \left[\frac{y_{i}-b^{'}\left(\eta_{i}\right)}{a}x_{i}^{T}\right]}{\partial \beta }\right]h\left(g,D_{i}\right)\;\;=\sum_{i=1}^{N}\;\;\left(-{\left\{p_{\theta }\left(y_{i},D_{i}|x_{i}\right)\right\}}^{-2}\right)\left[\sum_{g\in 𝒢}\;\;f_{\theta }\left(y_{i}|x_{i},g\right)\frac{y_{i}-b^{'}\left(\eta_{i}\right)}{a}x_{i}^{T}h\left(g,D_{i}\right)\right]\left[\sum_{g\in 𝒢}\;\;f_{\theta }\left(y_{i}|x_{i},g\right)\frac{y_{i}-b^{'}\left(\eta_{i}\right)}{a}gh\left(g,D_{i}\right)\right]\;\;+{\left\{p_{\theta }\left(y_{i},D_{i}|x_{i}\right)\right\}}^{-1}\sum_{g\in 𝒢}\;\;f_{\theta }\left(y_{i}|x_{i},g\right)\left[\left(\frac{y_{i}-b^{'}\left(\eta_{i}\right)}{a}x_{i}^{T}\right)\left(\frac{y_{i}-b^{'}\left(\eta_{i}\right)}{a}g\right)-\frac{b^{''}\left(\eta_{i}\right)}{a}x_{i}^{T}g\right]h\left(g,D_{i}\right)\;\;=\sum_{i=1}^{N}\;\;\left(-{\left\{p_{\theta }\left(y_{i},D_{i}|x_{i}\right)\right\}}^{-2}\right)\left[\sum_{g\in 𝒢}\;\;f_{\theta }\left(y_{i}|x_{i},g\right)\frac{y_{i}-b^{'}\left(\eta_{i}\right)}{a}x_{i}^{T}h\left(g,D_{i}\right)\right]\left[\sum_{g\in 𝒢}\;\;f_{\theta }\left(y_{i}|x_{i},g\right)\frac{y_{i}-b^{'}\left(\eta_{i}\right)}{a}gh\left(g,D_{i}\right)\right]\;\;+{\left\{p_{\theta }\left(y_{i},D_{i}|x_{i}\right)\right\}}^{-1}\sum_{g\in 𝒢}\;\;f_{\theta }\left(y_{i}|x_{i},g\right)\left[\frac{{\left(y_{i}-b^{'}\left(\eta_{i}\right)\right)}^{2}}{a^{2}}-\frac{b^{''}\left(\eta_{i}\right)}{a}\right]x_{i}^{T}gh\left(g,D_{i}\right)$$

$$\frac{\partial^{2}l_{y,D}(\alpha ,\beta )}{\partial \beta^{T}\partial \beta }=\frac{\partial \frac{\partial l_{y,D}(\alpha ,\beta )}{\partial \beta^{T}}}{\partial \beta }\;\;=\frac{\partial \left[\sum_{i=1}^{N}\;\;{\left\{p_{\theta }\left(y_{i},D_{i}|x_{i}\right)\right\}}^{-1}\sum_{g\in 𝒢}\;\;f_{\theta }\left(y_{i}|x_{i},g\right)\frac{y_{i}-b^{'}\left(\eta_{i}\right)}{a}g^{T}h\left(g,D_{i}\right)\right]}{\partial \beta }\;\;=\sum_{i=1}^{N}\;\;\frac{\partial \left[{\left\{p_{\theta }\left(y_{i},D_{i}|x_{i}\right)\right\}}^{-1}\sum_{g\in 𝒢}\;\;f_{\theta }\left(y_{i}|x_{i},g\right)\frac{y_{i}-b^{'}\left(\eta_{i}\right)}{a}g^{T}h\left(g,D_{i}\right)\right]}{\partial \beta }\;\;=\sum_{i=1}^{N}\;\;\left[\sum_{g\in 𝒢}\;\;f_{\theta }\left(y_{i}|x_{i},g\right)\frac{y_{i}-b^{'}\left(\eta_{i}\right)}{a}g^{T}h\left(g,D_{i}\right)\right]\frac{\partial {\left\{p_{\theta }\left(y_{i},D_{i}|x_{i}\right)\right\}}^{-1}}{\partial \beta }+{\left\{p_{\theta }\left(y_{i},D_{i}|x_{i}\right)\right\}}^{-1}\frac{\partial \left[\sum_{g\in 𝒢}\;\;f_{\theta }\left(y_{i}|x_{i},g\right)\frac{y_{i}-b^{'}\left(\eta_{i}\right)}{a}g^{T}h\left(g,D_{i}\right)\right.}{\partial \beta }\;\;=\sum_{i=1}^{N}\;\;\left[\sum_{g\in 𝒢}\;\;f_{\theta }\left(y_{i}|x_{i},g\right)\frac{y_{i}-b^{'}\left(\eta_{i}\right)}{a}g^{T}h\left(g,D_{i}\right)\right]\left[\left(-{\left\{p_{\theta }\left(y_{i},D_{i}|x_{i}\right)\right\}}^{-2}\right)\sum_{g\in 𝒢}\;\;f_{\theta }\left(y_{i}|x_{i},g\right)\frac{y_{i}-b^{'}\left(\eta_{i}\right)}{a}gh\left(g,D_{i}\right)\right]\;\;+{\left\{p_{\theta }\left(y_{i},D_{i}|x_{i}\right)\right\}}^{-1}\sum_{g\in 𝒢}\;\;\frac{\partial \left[f_{\theta }\left(y_{i}|x_{i},g\right)\frac{y_{i}-b^{'}\left(\eta_{i}\right)}{a}g^{T}\right]}{\partial \beta }h\left(g,D_{i}\right)\;\;=\sum_{i=1}^{N}\;\;\left(-{\left\{p_{\theta }\left(y_{i},D_{i}|x_{i}\right)\right\}}^{-2}\right)\left[\sum_{g\in 𝒢}\;\;f_{\theta }\left(y_{i}|x_{i},g\right)\frac{y_{i}-b^{'}\left(\eta_{i}\right)}{a}g^{T}h\left(g,D_{i}\right)\right]\left[\sum_{g\in 𝒢}\;\;f_{\theta }\left(y_{i}|x_{i},g\right)\frac{y_{i}-b^{'}\left(\eta_{i}\right)}{a}gh\left(g,D_{i}\right)\right]\;\;+{\left\{p_{\theta }\left(y_{i},D_{i}|x_{i}\right)\right\}}^{-1}\sum_{g\in 𝒢}\;\;f_{\theta }\left(y_{i}|x_{i},g\right)\left[\left(\frac{y_{i}-b^{'}\left(\eta_{i}\right)}{a}g^{T}\right)\frac{\partial \left[\frac{y_{i}\eta_{i}-b\left(\eta_{i}\right)}{a}+c\left(y_{i}\right)\right]}{\partial \beta }+\frac{\partial \left[\frac{y_{i}-b^{'}\left(\eta_{i}\right)}{a}g^{T}\right]}{\partial \beta }\right]h\left(g,D_{i}\right)\;\;=\sum_{i=1}^{N}\;\;\left(-{\left\{p_{\theta }\left(y_{i},D_{i}|x_{i}\right)\right\}}^{-2}\right)\left[\sum_{g\in 𝒢}\;\;f_{\theta }\left(y_{i}|x_{i},g\right)\frac{y_{i}-b^{'}\left(\eta_{i}\right)}{a}g^{T}h\left(g,D_{i}\right)\right]\left[\sum_{g\in 𝒢}\;\;f_{\theta }\left(y_{i}|x_{i},g\right)\frac{y_{i}-b^{'}\left(\eta_{i}\right)}{a}gh\left(g,D_{i}\right)\right]\;\;+{\left\{p_{\theta }\left(y_{i},D_{i}|x_{i}\right)\right\}}^{-1}\sum_{g\in 𝒢}\;\;f_{\theta }\left(y_{i}|x_{i},g\right)\left[\left(\frac{y_{i}-b^{'}\left(\eta_{i}\right)}{a}g^{T}\right)\left(\frac{y_{i}-b^{'}\left(\eta_{i}\right)}{a}g\right)-\frac{b^{''}\left(\eta_{i}\right)}{a}g^{T}g\right]h\left(g,D_{i}\right)\;\;=\sum_{i=1}^{N}\;\;\left(-{\left\{p_{\theta }\left(y_{i},D_{i}|x_{i}\right)\right\}}^{-2}\right)\left[\sum_{g\in 𝒢}\;\;f_{\theta }\left(y_{i}|x_{i},g\right)\frac{y_{i}-b^{'}\left(\eta_{i}\right)}{a}g^{T}h\left(g,D_{i}\right)\right]\left[\sum_{g\in 𝒢}\;\;f_{\theta }\left(y_{i}|x_{i},g\right)\frac{y_{i}-b^{'}\left(\eta_{i}\right)}{a}gh\left(g,D_{i}\right)\right]\;\;+{\left\{p_{\theta }\left(y_{i},D_{i}|x_{i}\right)\right\}}^{-1}\sum_{g\in 𝒢}\;\;f_{\theta }\left(y_{i}|x_{i},g\right)\left[\frac{{\left(y_{i}-b^{'}\left(\eta_{i}\right)\right)}^{2}}{a^{2}}-\frac{b^{''}\left(\eta_{i}\right)}{a}\right]g^{T}gh\left(g,D_{i}\right)$$

By matrix transposition, we can calculate the following term:

$$\frac{\partial^{2}l_{y,D}(\alpha ,\beta )}{\partial \beta^{T}\partial \alpha }={\left(\frac{\partial^{2}l_{y,D}(\alpha ,\beta )}{\partial \alpha^{T}\partial \beta }\right)}^{T}.$$

## Appendix H Evaluation of Score Function at Constrained MLE when Parameters are (α,β)

We evaluated the score function at the constrained MLE under the null hypothesis, i.e. $\tilde{\theta }=(\tilde{\alpha },0)$. Under the null hypothesis, We have

$$p_{\tilde{\theta }}\left(y_{i},D_{i}|x_{i}\right)=\sum_{g\in 𝒢}\;f_{\tilde{\theta }}\left(y_{i}|x_{i},g\right)h\left(g,D_{i}\right)=\sum_{g\in 𝒢}\;f_{\tilde{\theta }}\left(y_{i}|x_{i}\right)h\left(g,D_{i}\right)=f_{\tilde{\theta }}\left(y_{i}|x_{i}\right)\sum_{g\in 𝒢}\;h\left(g,D_{i}\right)$$

and ${\tilde{\eta }}_{i}=\tilde{\eta }\left(x_{i},g\right)=\tilde{\eta }\left(x_{i}\right)=\tilde{\alpha }x_{i}^{T}$. Note that under the null hypothesis, the term ${\tilde{\eta }}_{i}=\tilde{\alpha }x_{i}^{T}$ does not include *g*. Thus

$$\begin{array}{l} s_{y,D}(\tilde{\alpha },0)=\sum_{i=1}^{N}\left[{\left\{p_{\tilde{\theta }}\left(y_{i},D_{i}|x_{i}\right)\right\}}^{-1}\sum_{g\in 𝒢}f_{\tilde{\theta }}\left(y_{i}|x_{i},g\right)\left[\begin{array}{c} \frac{y_{i}-b^{\prime }\left({\hat{\eta }}_{i}\right)}{a}x_{i}^{T}\\ \frac{y_{i}-b^{\prime }\left({\hat{\eta }}_{i}\right)}{a}g^{T} \end{array}\right]h\left(g,D_{i}\right)\right]\\ =\sum_{i=1}^{N}\left[{\left\{f_{\tilde{\theta }}\left(y_{i}|x_{i}\right)\sum_{g\in 𝒢}h\left(g,D_{i}\right)\right\}}^{-1}f_{\tilde{\theta }}\left(y_{i}|x_{i}\right)\sum_{g\in 𝒢}\left[\begin{array}{c} \frac{y_{i}-b^{\prime }\left({\tilde{\eta }}_{i}\right)}{a}x_{i}^{T}\\ \frac{y_{i}-b^{\prime }\left({\tilde{\eta }}_{i}\right)}{a}g^{T} \end{array}\right]h\left(g,D_{i}\right)\right]\\ \sum_{i=1}^{N}\left[{\left\{\sum_{g\in 𝒢}h\left(g,D_{i}\right)\right\}}^{-1}\sum_{g\in 𝒢}\left[\begin{array}{l} \frac{y_{i}-b^{\prime }\left({\hat{\eta }}_{i}\right)}{a}\\ \frac{y_{i}-b^{\prime }\left({\hat{\eta }}_{i}\right)}{a}g_{i}^{T} \end{array}\right]h\left(g,D_{i}\right)\right]\\ =\sum_{i=1}^{N}\left[\left[\begin{array}{l} {\left\{\sum_{g\in 𝒢}h\left(g,D_{i}\right)\right\}}^{-1}\sum_{g\in 𝒢}\frac{y_{i}-b^{\prime }\left({\hat{\eta }}_{i}\right)}{a}x_{i}^{T}\left\{h\left(g,D_{i}\right)\right\}\\ {\left\{\sum_{g\in 𝒢}h\left(g,D_{i}\right)\right\}}^{-1}\sum_{g\in 𝒢}\frac{y_{i}-b^{\prime }\left({\hat{\eta }}_{i}\right)}{a}g^{T}\left\{h\left(g,D_{i}\right)\right\} \end{array}\right]\right]\\ =\sum_{i=1}^{N}\left[\left[\begin{array}{l} {\left\{\sum_{g\in 𝒢}h\left(g,D_{i}\right)\right\}}^{-1}\frac{y_{i}-b^{\prime }\left({\hat{\eta }}_{i}\right)}{a}x_{i}^{T}\left\{\sum_{g\in 𝒢}h\left(g,D_{i}\right)\right\}\\ {\left\{\sum_{g\in 𝒢}h\left(g,D_{i}\right)\right\}}^{-1}\sum_{g\in 𝒢}\frac{y_{i}-b^{\prime }\left({\tilde{\eta }}_{i}\right)}{a}g^{T}\left\{h\left(g,D_{i}\right)\right\} \end{array}\right]\right]\\ =\left[\begin{array}{c} \sum_{i=1}^{N}\frac{y_{i}-b^{\prime }\left({\hat{\eta }}_{i}\right)}{a}x_{i}^{T}\\ \sum_{i=1}^{N}\left[\frac{y_{i}-b^{\prime }\left({\hat{\eta }}_{i}\right)}{a}{\left\{\sum_{g\in 𝒢}h\left(g,D_{i}\right)\right\}}^{-1}\sum_{g\in 𝒢}g^{T}\left\{h\left(g,D_{i}\right)\right\}\right] \end{array}\right]\\ =\left[\begin{array}{c} 0\\ \sum_{i=1}^{N}\frac{y_{i}-b^{\prime }\left(\hat{\alpha }x_{i}^{T}\right)}{a}E\left(g^{T}|D_{i}\right) \end{array}\right], \end{array}$$

where $E\left(g^{T}|D_{i}\right)={\left\{\sum_{g\in 𝒢}\;h\left(g,D_{i}\right)\right\}}^{-1}\sum_{g\in 𝒢}\;g^{T}h\left(g,D_{i}\right)=\left(\sum_{g\in 𝒢}\;g^{T}h\left(g,D_{i}\right)\right)/\left(\sum_{g\in 𝒢}\;h\left(g,D_{i}\right)\right)$ is the posterior expectation of the genotype of individual i given sequencing data $D_{i}$.

## Appendix I Evaluation of Observed Information Matrix at Constrained MLE when Parameters are (α,β)

$$\begin{array}{ll} & {\left.\frac{\partial^{2}l_{y,D}(\alpha ,\beta )}{\partial \alpha^{T}\partial \alpha }\right|}_{\tilde{\theta }}\\ = & \;\sum_{i=1}^{N}\;\;\left(-{\left\{p_{\theta }\left(y_{i},D_{i}|x_{i}\right)\right\}}^{-2}\right)\left[\sum_{g\in 𝒢}\;\;f_{\theta }\left(y_{i}|x_{i},g\right)\frac{y_{i}-b^{'}\left({\tilde{\eta }}_{i}\right)}{a}x_{i}^{T}h\left(g,D_{i}\right)\right]\left[\sum_{g\in 𝒢}\;\;f_{\theta }\left(y_{i}|x_{i},g\right)\frac{y_{i}-b^{'}\left({\tilde{\eta }}_{i}\right)}{a}x_{i}h\left(g,D_{i}\right)\right]\\ & \;+{\left\{p_{\theta }\left(y_{i},D_{i}|x_{i}\right)\right\}}^{-1}\sum_{g\in 𝒢}\;\;f_{\theta }\left(y_{i}|x_{i},g\right)\left[\frac{{\left(y_{i}-b^{'}\left({\tilde{\eta }}_{i}\right)\right)}^{2}}{a^{2}}-\frac{b^{''}\left({\tilde{\eta }}_{i}\right)}{a}\right]x_{i}^{T}x_{i}h\left(g,D_{i}\right)\\ = & \;\sum_{i=1}^{N}\;\;\left(-{\left\{f_{\theta }\left(y_{i}|x_{i}\right)\sum_{g\in 𝒢}\;\;h\left(g,D_{i}\right)\right\}}^{-2}\right)\left[f_{\theta }\left(y_{i}|x_{i}\right)\frac{y_{i}-b^{'}\left({\tilde{\eta }}_{i}\right)}{a}x_{i}^{T}\sum_{g\in 𝒢}\;\;h\left(g,D_{i}\right)\right]\left[f_{\theta }\left(y_{i}|x_{i}\right)\frac{y_{i}-b^{'}\left({\tilde{\eta }}_{i}\right)}{a}x_{i}\sum_{g\in 𝒢}\;\;h\left(g,D_{i}\right)\right]\\ & \;+{\left\{f_{\theta }\left(y_{i}|x_{i}\right)\sum_{g\in 𝒢}\;\;h\left(g,D_{i}\right)\right\}}^{-1}f_{\theta }\left(y_{i}|x_{i}\right)\left[\frac{{\left(y_{i}-b^{'}\left({\tilde{\eta }}_{i}\right)\right)}^{2}}{a^{2}}-\frac{b^{''}\left({\tilde{\eta }}_{i}\right)}{a}\right]x_{i}^{T}x_{i}\sum_{g\in 𝒢}\;\;h\left(g,D_{i}\right)\\ = & \;\sum_{i=1}^{N}\;\;(-1)\left[\frac{y_{i}-b^{'}\left({\tilde{\eta }}_{i}\right)}{a}x_{i}^{T}\right]\left[\frac{y_{i}-b^{'}\left({\tilde{\eta }}_{i}\right)}{a}x_{i}\right]+\left[\frac{{\left(y_{i}-b^{'}\left({\tilde{\eta }}_{i}\right)\right)}^{2}}{a^{2}}-\frac{b^{''}\left({\tilde{\eta }}_{i}\right)}{a}\right]x_{i}^{T}x_{i}\\ = & \;-\frac{1}{a}\sum_{i=1}^{N}\;\;b^{''}\left(\tilde{\alpha }x_{i}^{T}\right)x_{i}^{T}x_{i}. \end{array}$$

$$\begin{array}{ll} & {\left.\frac{\partial^{2}l_{y,D}(\alpha ,\beta )}{\partial \alpha^{T}\partial \beta }\right|}_{\tilde{\theta }}\\ = & \;\sum_{i=1}^{N}\;\;\left(-{\left\{p_{\theta }\left(y_{i},D_{i}|x_{i}\right)\right\}}^{-2}\right)\left[\sum_{g\in 𝒢}\;\;f_{\theta }\left(y_{i}|x_{i},g\right)\frac{y_{i}-b^{'}\left({\tilde{\eta }}_{i}\right)}{a}x_{i}^{T}h\left(g,D_{i}\right)\right]\left[\sum_{g\in 𝒢}\;\;f_{\theta }\left(y_{i}|x_{i},g\right)\frac{y_{i}-b^{'}\left({\tilde{\eta }}_{i}\right)}{a}gh\left(g,D_{i}\right)\right]\\ & \;+{\left\{p_{\theta }\left(y_{i},D_{i}|x_{i}\right)\right\}}^{-1}\sum_{g\in 𝒢}\;\;f_{\theta }\left(y_{i}|x_{i},g\right)\left[\frac{{\left(y_{i}-b^{'}\left({\tilde{\eta }}_{i}\right)\right)}^{2}}{a^{2}}-\frac{b^{''}\left({\tilde{\eta }}_{i}\right)}{a}\right]x_{i}^{T}gh\left(g_{,}D_{i}\right)\\ = & \;\sum_{i=1}^{N}\;\;\left(-{\left\{f_{\theta }\left(y_{i}|x_{i}\right)\sum_{g\in 𝒢}\;\;h\left(g,D_{i}\right)\right\}}^{-2}\right)\left[f_{\theta }\left(y_{i}|x_{i}\right)\frac{y_{i}-b^{'}\left({\tilde{\eta }}_{i}\right)}{a}x_{i}^{T}\sum_{g\in 𝒢}\;\;h\left(g,D_{i}\right)\right]\left[f_{\theta }\left(y_{i}|x_{i}\right)\frac{y_{i}-b^{'}\left({\tilde{\eta }}_{i}\right)}{a}\sum_{g\in 𝒢}\;\;gh\left(g,D_{i}\right)\right]\\ & \;+{\left\{f_{\theta }\left(y_{i}|x_{i}\right)\sum_{g\in 𝒢}\;\;h\left(g,D_{i}\right)\right\}}^{-1}f_{\theta }\left(y_{i}|x_{i}\right)\left[\frac{{\left(y_{i}-b^{'}\left({\tilde{\eta }}_{i}\right)\right)}^{2}}{a^{2}}-\frac{b^{''}\left({\tilde{\eta }}_{i}\right)}{a}\right]x_{i}^{T}\sum_{g\in 𝒢}\;\;gh\left(g,D_{i}\right)\\ = & \;\sum_{i=1}^{N}\;\;\left(-{\left\{\sum_{g\in 𝒢}\;\;h\left(g,D_{i}\right)\right\}}^{-2}\right)\left[\frac{y_{i}-b^{'}\left({\tilde{\eta }}_{i}\right)}{a}x_{i}^{T}\sum_{g\in 𝒢}\;\;h\left(g,D_{i}\right)\right]\left[\frac{y_{i}-b^{'}\left({\tilde{\eta }}_{i}\right)}{a}\sum_{g\in 𝒢}\;\;gh\left(g,D_{i}\right)\right]\\ & \;+{\left\{\sum_{g\in 𝒢}\;\;h\left(g,D_{i}\right)\right\}}^{-1}\left[\frac{{\left(y_{i}-b^{'}\left({\tilde{\eta }}_{i}\right)\right)}^{2}}{a^{2}}-\frac{b^{''}\left({\tilde{\eta }}_{i}\right)}{a}\right]x_{i}^{T}\sum_{g\in 𝒢}\;\;gh\left(g,D_{i}\right)\\ = & \;\sum_{i=1}^{N}\;\;\left(-{\left\{\sum_{g\in 𝒢}\;\;h\left(g,D_{i}\right)\right\}}^{-1}\right)\left[\frac{y_{i}-b^{'}\left({\tilde{\eta }}_{i}\right)}{a}x_{i}^{T}\right]\left[\frac{y_{i}-b^{'}\left({\tilde{\eta }}_{i}\right)}{a}\sum_{g\in 𝒢}\;\;gh\left(g,D_{i}\right)\right]\\ & \;+{\left\{\sum_{g\in 𝒢}\;\;h\left(g,D_{i}\right)\right\}}^{-1}\left[\frac{{\left(y_{i}-b^{'}\left({\tilde{\eta }}_{i}\right)\right)}^{2}}{a^{2}}-\frac{b^{''}\left({\tilde{\eta }}_{i}\right)}{a}\right]x_{i}^{T}\sum_{g\in 𝒢}\;\;gh\left(g,D_{i}\right)\\ = & \;-\sum_{i=1}^{N}\;\;{\left\{\sum_{g\in 𝒢}\;\;h\left(g,D_{i}\right)\right\}}^{-1}\frac{b^{''}\left({\tilde{\eta }}_{i}\right)}{a}x_{i}^{T}\sum_{g\in 𝒢}\;\;gh\left(g,D_{i}\right)=-\sum_{i=1}^{N}\;\;\frac{b^{''}\left({\tilde{\eta }}_{i}\right)}{a}x_{i}^{T}{\left\{\sum_{g\in 𝒢}\;\;h\left(g,D_{i}\right)\right\}}^{-1}\sum_{g\in 𝒢}\;\;gh\left(g,D_{i}\right)\\ = & \;-\frac{1}{a}\sum_{i=1}^{N}\;\;b^{''}\left(\tilde{\alpha }x_{i}^{T}\right)x_{i}^{T}E\left(g|D_{i}\right) \end{array}$$

$$\begin{array}{ll} & {\left.\frac{\partial^{2}l_{y,D}(\alpha ,\beta )}{\partial \beta^{T}\partial \beta }\right|}_{\tilde{\theta }}\\ & \;=\sum_{i=1}^{N}\;\;\left(-{\left\{p_{\theta }\left(y_{i},D_{i}|x_{i}\right)\right\}}^{-2}\right)\left[\sum_{g\in 𝒢}\;\;f_{\theta }\left(y_{i}|x_{i},g\right)\frac{y_{i}-b^{'}\left({\tilde{\eta }}_{i}\right)}{a}g^{T}h\left(g,D_{i}\right)\right]\left[\sum_{g\in 𝒢}\;\;f_{\theta }\left(y_{i}|x_{i},g\right)\frac{y_{i}-b^{'}\left({\tilde{\eta }}_{i}\right)}{a}gh\left(g,D_{i}\right)\right]\\ & \;+{\left\{p_{\theta }\left(y_{i},D_{i}|x_{i}\right)\right\}}^{-1}\sum_{g\in 𝒢}\;\;f_{\theta }\left(y_{i}|x_{i},g\right)\left[\frac{{\left(y_{i}-b^{'}\left({\tilde{\eta }}_{i}\right)\right)}^{2}}{a^{2}}-\frac{b^{''}\left({\tilde{\eta }}_{i}\right)}{a}\right]g^{T}gh\left(g,D_{i}\right)\\ & \;=\sum_{i=1}^{N}\;\;\left(-{\left\{f_{\theta }\left(y_{i}|x_{i}\right)\sum_{g\in 𝒢}\;\;h\left(g,D_{i}\right)\right\}}^{-2}\right)\left[\sum_{g\in 𝒢}\;\;f_{\theta }\left(y_{i}|x_{i},g\right)\frac{y_{i}-b^{'}\left({\tilde{\eta }}_{i}\right)}{a}g^{T}h\left(g,D_{i}\right)\right]\left[\sum_{g\in 𝒢}\;\;f_{\theta }\left(y_{i}|x_{i},g\right)\frac{y_{i}-b^{'}\left({\tilde{\eta }}_{i}\right)}{a}gh\left(g,D_{i}\right)\right]\\ & \;+{\left\{f_{\theta }\left(y_{i}|x_{i}\right)\sum_{g\in 𝒢}\;\;h\left(g,D_{i}\right)\right\}}^{-1}\sum_{g\in 𝒢}\;\;f_{\theta }\left(y_{i}|x_{i},g\right)\left[\frac{{\left(y_{i}-b^{'}\left({\tilde{\eta }}_{i}\right)\right)}^{2}}{a^{2}}-\frac{b^{''}\left({\tilde{\eta }}_{i}\right)}{a}\right]g^{T}gh\left(g,D_{i}\right)\\ & \;=\sum_{i=1}^{N}\;\;\left(-{\left\{f_{\theta }\left(y_{i}|x_{i}\right)\sum_{g\in 𝒢}\;\;h\left(g,D_{i}\right)\right\}}^{-2}\right)\left[f_{\theta }\left(y_{i}|x_{i}\right)\frac{y_{i}-b^{'}\left({\tilde{\eta }}_{i}\right)}{a}\sum_{g\in 𝒢}\;\;g^{T}h\left(g,D_{i}\right)\right]\left[f_{\theta }\left(y_{i}|x_{i}\right)\frac{y_{i}-b^{'}\left({\tilde{\eta }}_{i}\right)}{a}\sum_{g\in 𝒢}\;\;gh\left(g,D_{i}\right)\right]\\ & \;+{\left\{f_{\theta }\left(y_{i}|x_{i}\right)\sum_{g\in 𝒢}\;\;h\left(g,D_{i}\right)\right\}}^{-1}f_{\theta }\left(y_{i}|x_{i}\right)\left[\frac{{\left(y_{i}-b^{'}\left({\tilde{\eta }}_{i}\right)\right)}^{2}}{a^{2}}-\frac{b^{''}\left({\tilde{\eta }}_{i}\right)}{a}\right]\sum_{g\in 𝒢}\;\;g^{T}gh\left(g,D_{i}\right)\\ & \;=\sum_{i=1}^{N}\;\;\left(-{\left\{\sum_{g\in 𝒢}\;\;h\left(g,D_{i}\right)\right\}}^{-2}\right)\left[\frac{y_{i}-b^{'}\left({\tilde{\eta }}_{i}\right)}{a}\sum_{g\in 𝒢}\;\;g^{T}h\left(g,D_{i}\right)\right]\left[\frac{y_{i}-b^{'}\left({\tilde{\eta }}_{i}\right)}{a}\sum_{g\in 𝒢}\;\;gh\left(g,D_{i}\right)\right]\\ & \;+{\left\{\sum_{g\in 𝒢}\;\;h\left(g,D_{i}\right)\right\}}^{-1}\left[\frac{{\left(y_{i}-b^{'}\left({\tilde{\eta }}_{i}\right)\right)}^{2}}{a^{2}}-\frac{b^{''}\left({\tilde{\eta }}_{i}\right)}{a}\right]\sum_{g\in 𝒢}\;\;g^{T}gh\left(g,D_{i}\right)\\ & \;=\sum_{i=1}^{N}\;\;(-1)\left[\frac{y_{i}-b^{'}\left({\tilde{\eta }}_{i}\right)}{a}{\left\{\sum_{g\in 𝒢}\;\;h\left(g,D_{i}\right)\right\}}^{-1}\sum_{g\in 𝒢}\;\;g^{T}h\left(g,D_{i}\right)\right]\left[\frac{y_{i}-b^{'}\left({\tilde{\eta }}_{i}\right)}{a}{\left\{\sum_{g\in 𝒢}\;\;h\left(g,D_{i}\right)\right\}}^{-1}\sum_{g\in 𝒢}\;\;gh\left(g,D_{i}\right)\right]\\ & \;+\left[\frac{{\left(y_{i}-b^{'}\left({\tilde{\eta }}_{i}\right)\right)}^{2}}{a^{2}}-\frac{b^{''}\left({\tilde{\eta }}_{i}\right)}{a}\right]{\left\{\sum_{g\in 𝒢}\;\;h\left(g,D_{i}\right)\right\}}^{-1}\sum_{g\in 𝒢}\;\;g^{T}gh\left(g,D_{i}\right)\\ & \;=\sum_{i=1}^{N}\;\;\left[\frac{{\left(y_{i}-b^{'}\left(\tilde{\alpha }x_{i}^{T}\right)\right)}^{2}}{a^{2}}\left\{E\left(g^{T}g|D_{i}\right)-E\left(g^{T}|D_{i}\right)E\left(g|D_{i}\right)\right\}-\frac{b^{''}\left(\tilde{\alpha }x_{i}^{T}\right)}{a}E\left(g^{T}g|D_{i}\right)\right] \end{array}$$

In summary, we have

$$\begin{array}{c} {\left.\frac{\partial^{2}l_{y,D}(\alpha ,\beta )}{\partial \alpha^{T}\partial \alpha }\right|}_{\tilde{\theta }}=-\frac{1}{a}\sum_{i=1}^{N}\;\;b^{''}\left(\tilde{\alpha }x_{i}^{T}\right)x_{i}^{T}x_{i}\\ {\left.\frac{\partial^{2}l_{y,D}(\alpha ,\beta )}{\partial \alpha^{T}\partial \beta }\right|}_{\tilde{\theta }}=-\frac{1}{a}\sum_{i=1}^{N}\;\;b^{''}\left(\tilde{\alpha }x_{i}^{T}\right)x_{i}^{T}E\left(g|D_{i}\right)\\ {\left.\frac{\partial^{2}l_{y,D}(\alpha ,\beta )}{\partial \beta^{T}\partial \beta }\right|}_{\tilde{\theta }}=\sum_{i=1}^{N}\;\;\left[\frac{{\left(y_{i}-b^{'}\left(\tilde{\alpha }x_{i}^{T}\right)\right)}^{2}}{a^{2}}\left(E\left(g^{T}g|D_{i}\right)-E\left(g^{T}|D_{i}\right)E\left(g|D_{i}\right)\right)-\frac{b^{''}\left(\tilde{\alpha }x_{i}^{T}\right)}{a}E\left(g^{T}g|D_{i}\right)\right]. \end{array}$$

By matrix transposition, we can obtain the following term:

$${\left.\frac{\partial^{2}l_{y,D}(\alpha ,\beta )}{\partial \beta^{T}\partial \alpha }\right|}_{\tilde{\theta }}={\left({\left.\frac{\partial^{2}l_{y,D}(\alpha ,\beta )}{\partial \alpha^{T}\partial \beta }\right|}_{\tilde{\theta }}\right)}^{T}.$$

## Abbreviations

AF: Allele frequency
GC: Genotype calling
GLF: Genotype likelihood function
GLM: Generalized linear model
JS: Joint significance
LM: Linear model
LRT: Likelihood ratio test
MAD: Mean absolute deviation
MSE: Mean squared error
NGS: Next-generation sequencing
SKAT: Sequence kernel association test
VC: Variable collapse
